# Survey of genome sequences in a wild sweet potato, *Ipomoea trifida* (H. B. K.) G. Don

**DOI:** 10.1093/dnares/dsv002

**Published:** 2015-03-24

**Authors:** Hideki Hirakawa, Yoshihiro Okada, Hiroaki Tabuchi, Kenta Shirasawa, Akiko Watanabe, Hisano Tsuruoka, Chiharu Minami, Shinobu Nakayama, Shigemi Sasamoto, Mitsuyo Kohara, Yoshie Kishida, Tsunakazu Fujishiro, Midori Kato, Keiko Nanri, Akiko Komaki, Masaru Yoshinaga, Yasuhiro Takahata, Masaru Tanaka, Satoshi Tabata, Sachiko N. Isobe

**Affiliations:** 1 Kazusa DNA Research Institute, Kisarazu, Chiba 292-0818, Japan; 2 Crop and Agribusiness Research Division, Kyushu Okinawa Agricultural Research Center, National Agriculture and Food Research Organization (NARO/KARC), Itoman, Okinawa 901-0336, Japan; 3 Upland Farming Research Division, Kyushu Okinawa Agricultural Research Center, National Agriculture and Food Research Organization (NARO/KARC), Miyakonojo, Miyazaki 885-0091, Japan

**Keywords:** *Ipomoea trifida*, genome sequence assembly, core- and line-specific sequences, SNPs, CNVs

## Abstract

*Ipomoea trifida* (H. B. K.) G. Don. is the most likely diploid ancestor of the hexaploid sweet potato, *I. batatas* (L.) Lam. To assist in analysis of the sweet potato genome, *de novo* whole-genome sequencing was performed with two lines of *I. trifida*, namely the selfed line Mx23Hm and the highly heterozygous line 0431-1, using the Illumina HiSeq platform. We classified the sequences thus obtained as either ‘core candidates’ (common to the two lines) or ‘line specific’. The total lengths of the assembled sequences of Mx23Hm (ITR_r1.0) was 513 Mb, while that of 0431-1 (ITRk_r1.0) was 712 Mb. Of the assembled sequences, 240 Mb (Mx23Hm) and 353 Mb (0431-1) were classified into core candidate sequences. A total of 62,407 (62.4 Mb) and 109,449 (87.2 Mb) putative genes were identified, respectively, in the genomes of Mx23Hm and 0431-1, of which 11,823 were derived from core sequences of Mx23Hm, while 28,831 were from the core candidate sequence of 0431-1. There were a total of 1,464,173 single-nucleotide polymorphisms and 16,682 copy number variations (CNVs) in the two assembled genomic sequences (under the condition of log_2_ ratio of >1 and CNV size >1,000 bases). The results presented here are expected to contribute to the progress of genomic and genetic studies of *I. trifida*, as well as studies of the sweet potato and the genus *Ipomoea* in general.

## 1. Introduction

*Ipomoea* is the largest genus in the family Convolvulaceae, which consists of 600–700 species.^1^ Sweet potato (*Ipomoea batatas* (L.) Lam) is the only species in the genus *Ipomoea* that is widely cultivated and consumed as a crop around the world. It has a complex genome structure, with hexaploidy (2*N* = 6*x* = 90), and a large genome size (4.8–5.3 pg/2C nucleus).^2^ The complex nature of the sweet potato genome has obstructed genetic studies on agronomically important characteristics such as self- and cross-incompatibility,^3^ and thus, the progress in genetics and genomics in this species lags far behind that in other important crop species.

*I. trifida* (H. B. K.) G. Don., a wild relative of sweet potato distributed around the Caribbean Sea, forms a polyploidy complex ranging from diploid (2*N* = 2*x* = 30) to hexaploid (2*N* = 6*x* = 90).^4^ *I. trifida* and sweet potato are closely related, because they are cross-compatible.^5–7^ Molecular genetic^8–11^ and cytogenetic^12^ data also support the close relationship of these two species. The discussion of polyploidization process in sweet potato is not complete; however, Shiotani and Kawase^5^ suggested that sweet potato is an autohexaploid derived from diploid *I. trifida* based on cytogenetic analysis of a series of interspecific hybrids between sweet potato and *I. trifida*. In addition, molecular genetic data from simple sequence repeats (SSRs), non-coding chloroplast and nuclear ITS sequences implied an autohexaploid origin of sweet potato from an ancestor it shares with *I. trifida*.^11^

Because of its cross-compatibility, status as the closest wild relative, and varied polyploidy, *I. trifida* is considered as a model species of sweet potato and is therefore used for genetic, physiological, and cytological analyses. In particular, the self-incompatibility system has often been studied in *I. trifida,* with the goal of achieving random crossing in sweet potato breeding in the future.^13–16^ However, limited genetic and genomic resources have been developed in *I. trifida*. An amplified fragment length polymorphism (AFLP)-based linkage map for this species was first developed by Nakayama *et al.*^17^ The nucleotide sequences available to date (November 2014) in GenBank include 1,407 expressed sequence tags (ESTs) and 642 nucleotide or genome survey sequences.

With the advances in next-generation sequencing (NGS) technology, *de novo* whole-genome sequencing is no longer limited to a few plant species: to date, the whole-genome sequences of >50 plant species have been published.^18^ Under these circumstances, plant scientists are further focussing on variations in genomes, with the goal of understanding the overall genome structure of a variety of germplasms with different characteristics of individual species. Whole-genome re-sequencing of multiple lines has been performed in several plant species, including *Arabidopsis thaliana*,^19^ rice,^20^ and maize.^21^

The accumulating information on the genome structures of plant germplasms has led to our interest in the ‘pan-genome concept’,^22^ which was first proposed by Tettelin *et al.*^23^ in reference to the *Streptococcus agalactiae* genome. The pan-genome consists of a core genome that is present in all strains, and a dispensable genome composed of partially shared and strain-specific DNA sequences. Analyses of plant genomes based on a pan-genome perspective have already been performed in a few plant species to better understand the process of evolution and to accelerate the breeding process.^24,25^ In addition, investigation of structural variations (SVs), defined as genomic variations in the size range above 1 kb, using the NGS technology has also become more widespread in plant genomics.^26^ Genome sequencing by NGS can be straightforwardly adapted to validation of SVs, especially copy number variations (CNVs) and presence–absence variations. Detection of SVs throughout the genome, along with base-level variations such as single-nucleotide polymorphisms (SNPs), is expected to contribute to our understanding of phenotypic variation in species.

*I. trifida* generally exhibits severe self-incompatibility and maintains heterozygosity within an accession. However, self-fertile lines were recently discovered by Kowyama *et al.*^13^ and Nakayama *et al.*^17^ Using one such self-fertile line, we developed a single descendant selfed line (S_11_), named Mx23Hm, which is the progeny of a paternal line (Mx23-4) of a population used for construction of the first linkage map in *I. trifida*.^17^ In this study, we performed *de novo* whole-genome sequencing for Mx23Hm using the Illumina sequencing platform. Whole-genome sequencing was also carried out for another *I. trifida* line, 0431-1, which exhibits heterozygosity and was used as the maternal line for the first linkage map. The independently assembled genomic sequences of both lines were classified as either ‘core candidates' (common to the two lines) or ‘line specific’. CNVs and SNPs in the two assembled sequences were also investigated to understand genome-wide variation in *I. trifida*. This is the first report of *de novo* whole-genome sequencing in the genus *Ipomoea*, and the results are expected to contribute to genomic and genetic analysis of *I. trifida*, as well as of sweet potato and the genus *Ipomoea* in general.

## 2. Materials and methods

### 2.1. Plant materials

Two lines of diploid *I. trifida*, Mx23Hm and 0431-1, were subjected to genome sequencing. Mx23Hm is a single descendant selfed line (S_11_) derived from the self-compatible experimental line Mx23-4, which was introduced from Mexico to Japan in 1961 and deposited in the *Ipomoea* collection of NARO/KARC. 0431-1 is a self-incompatible experimental line obtained by crosses between several diploid *I. trifida* lines introduced from Mexico and Colombia in 1973 and 1980, respectively. Genomic DNA was extracted from young leaves using the DNeasy Plant Mini Kit (Qiagen, Valencia, CA, USA) or a modified CTAB method.^27^ DNA quantitation and quality checks were performed using a NanoDrop ND1000 spectrophotometer (NanoDrop Technologies, Wilmington, DE, USA) and 0.8% agarose gel electrophoresis, respectively.

Reduction of heterozygosity in the selfed descendants of Mx23-4 (S_1_, S_7_, and S_10_ generation) was monitored using 14 SSR markers that identified heterozygous alleles in the S_1_ plants. The 14 SSR markers were selected from 85 sweet potato EST-derived SSR markers developed at the Kazusa DNA Research Institute (unpublished). The primer sequences of the 14 SSR markers are listed in Supplementary Table S1. Genomic DNA was extracted from eight descent lines of each generation. Amplification of SSR markers was performed using a modified touchdown PCR protocol^28^ in 20 μl reaction mixtures containing 20 ng of DNA, 200 μM dNTPs, 1 μM of each primer, 0.5 units of *Taq* DNA polymerase, and 1× *Taq* polymerase buffer. Five microlitres of each PCR product was subjected to electrophoresis on a MultiNA MCE-202 system (SHIMADZU Biotech, Tokyo).

### 2.2. Genome sequencing and genome size estimation

Paired-end (PE) and mate-pair (MP) libraries were prepared using total cellular DNA from Mx23Hm and 0431-1. The PE libraries of both lines and the MP library of Mx23Hm were constructed according to the instruction provided by Illumina (San Diego, CA, USA). A modified protocol proposed by Nieuwerburgh *et al.*^29^ was employed for MP library preparation for 0431-1. The read length was 101 bases, and the expected insert size ranged from 0.3 to 20 kb (Supplementary Table S2). Sequence analyses were carried out on an Illumina HiSeq 2000 platform.

The obtained reads were subjected to quality control as follows. Bases with quality scores <10 were filtered out by PRINSEQ 0.20.4.^30^ Adaptor sequences in the reads were trimmed using fastx_clipper of the FASTX-Toolkit 0.0.13 (http://hannonlab.cshl.edu/fastx_toolkit). After trimming, reads including *N* nucleotides, or with lengths <100 bases, were excluded. The genome size of *I. trifida* was estimated based on the k-mer frequency of the sequence reads (*k* = 17) using Jellyfish ver. 2.1.1.^31^

### 2.3. Sequence assembly

Two programs, SOAPdenovo2 r223^32^ and Platanus ver.1.2.1,^33^ were adopted for assembly of the Illumina PE reads. For SOAPdenovo2 r223, k-mer sizes from 41 to 95 were examined using default parameters, and the optimal k-mer size was selected from the N50 length in each k-mer size. The gaps on the scaffolds in each line were closed with the PE reads using GapCloser 1.10 (*P* = 31) (http://soap.genomics.org.cn/soapdenovo.html). The resultant sequences were then subjected to further scaffolding with the MP reads using SSPACE2.0 with the parameters (−k 3 −x 0).^34^ Potential contaminating sequences in the assembled scaffolds were detected and removed using BLASTN^35^ searches against the chloroplast genome sequence of potato (*Solanum tuberosum*; accession number: NC_008096.2), the mitochondrial sequence of *A. thaliana* (accession number: NC_001284.2), bacteria, fungi, and human genome sequences registered in NCBI (http://www.ncbi.nlm.nih.gov) and vector sequences from UniVec (http://www.ncbi.nlm.nih.gov/tools/vecscreen/univec/) with an *E*-value cut-off of 1E−10 and length coverage of ≥10%. The resultant scaffolds longer than 300 bases in length were selected and designated ITR_r1.0 for Mx23Hm and ITRk_r1.0 for 0431-1.

Assembly of the Illumina reads using Platanus was carried out for construction of the contigs and scaffolds. The gaps on the scaffolds were closed using a gap-closing program in Platanus. Authenticity of the assembled sequences was confirmed by comparing with the BAC (Bacterial Artificial Chromosome) contigs derived from haplotype S1 self-incompatibility (S-) locus of *I. trifida*^36^ registered in NCBI using a NUCmer program^37^ followed by a two-dimensional dot plot analysis. The accession numbers of these BAC contigs were AY448010.1 (82,054 bases), AY448011.1 (9,363 bases), AY448012.1 (31,590 bases), AY448013.1 (87,724 bases), AY448014.1 (7,191 bases), AY448015.1 (39,512 bases), and AY448016.1 (55,205 bases).^36^

Core candidate and line-specific sequences were classified using LAST^38^ ver. 490 with a score of 272 corresponding to an *E*-value cut-off of 1E−100. Repetitive sequences were detected using RepeatScount 1.0.5^39^ and RepeatMasker 3.30 (http://www.repeatmasker.org) as described by Hirakawa *et al.*^40^ SSR motifs were identified using the SciRoKo software^41^ in the ‘MISA’ mode with default parameters. The minimum numbers of SSR repeats for mono-, di-, tri-, tetra-, penta-, and hexa-nucleotides adopted for identification were 14, 7, 5, 4, 4, and 4, respectively.

### 2.4. Gene prediction and annotation

Transfer RNA genes were predicted using tRNAscan-SE ver. 1.23^42^ with default parameters, and ribosomal RNA (rRNA) genes were predicted by BLAST searches with an E-value cut-off of 1*E*-10. The *A. thaliana* 5.8S and 25S rRNAs (accession number: X52320.1) and 18S rRNA (accession number: X16077.1) were used as query sequences.

Protein-encoding sequences in the assembled genomic sequences were predicted by Augustus 2.7^43^ and geneid^44^ programs using the *A. thaliana* training set under the default parameters. The training set of tomato genes provided by Augustus 2.7 was also examined for comparison. Reciprocal best-hit analysis was performed to compare the results of the prediction by using *A. thaliana* and tomato training sets. Genes related to transposable elements (TEs) were detected by BLASTP searches against the NCBI non-redundant (nr) protein database (http://www.ncbi.nlm.nih.gov) with an *E*-value cut-off of 1E−10, and InterProScan^45^ searches against the InterPro database^46^ with an *E*-value cut-off of 1.0.

The putative genes of *I. trifida* were clustered by using the CD-hit program^47^ with the unigene sets of potato (http://potato.plantbiology.msu.edu/index.shtml) (*S. tuberosum*, PGSC DM v3.4, 56,218 genes), cassava (http://www.phytozome.net/cassava.php) (*Manihot esculenta*, v4.1, 34,151 genes), and *A. thaliana* (http://www.arabidopsis.org) (TAIR10, 35,386 genes) with the parameters *c* = 0.4 and aS = 0.4. Genes in the plant species described above were classified into the plant gene ontology (GO) slim categories,^48^ and the ‘euKaryotic clusters of Orthologous Groups' (KOG) categories^49^ and then mapped onto the Kyoto Encyclopedia of Genes and Genomes (KEGG) reference pathways^50^ as described by Hirakawa *et al.*^39^ Multiple alignment of amino acid sequences was performed for starch synthase homologues predicted in ITR_r1.0 using ClustalX,^51^ and a genetic tree was constructed using the neighbour-joining algorithm of MEGA6.^52^

### 2.5. SNP discovery and CNVs analysis

The trimmed PE reads for Mx23Hm and 0431-1 described above were independently mapped onto ITR_r1.0 using Bowtie 2 2.2.34 (parameter maxins = 1000).^53^ To eliminate possible alignment errors, the results with the following SAM Flags were employed: 83, 99, 147, and 163 for PE reads that were mapped on the same contig with correct read orientation and insert size, and 65, 81, 97, 113, 129, 145, 161, and 177 for PE reads that were mapped on unique positions of different contigs.

SNP candidates were called using samtools mpileup ver. 11.19 (parameters: -Duf -d 1000000),^54^ followed by filtration using VCFtools ver. 1.1.19^55^ and in-house Perl scripts according to the following criteria: (i) Bases at the SNP loci in Mx23Hm should exactly match the bases of ITR_r1.0 (to avoid false-positive SNP detection caused by sequencing or assembling error); (ii) all non-insertion or deletion mutations should not be included; (iii) quality scores of the SNP sites should be >200; and (iv) SNPs should not be located in repetitive sequences. SNP effects on the functions of putative genes in ITR_r1.0 were predicted using SnpEff ver. 4.0e (parameters: -no-downstream, -no-upstream).^56^ The *I. trifida* database for SNP annotation in SnpEff was constructed using the gff file generated by Augustus 3.0.2. CNVs were analysed using CNV-seq ver. 0.2.7 (parameters: -genome-size 239146348).^57^

## 3. Results

### 3.1. Establishment of a selfed line, Mx23Hm

Mx23-4, the parental line of Mx23Hm, exhibited less heterozygosity than 0431-1 in a previous study of linkage map construction.^17^ To obtain a highly homozygous descendant of Mx23-4, we performed successive selfing by the single seed descent method until generation S_11_. No heterozygous alleles were observed in any of the S_7_ and S_10_ descendants at 14 SSR loci that exhibited polymorphism in S_1_ generation (Supplementary Table S1 and Supplementary Fig. S1). Significant differences were not observed among the various generations in regard to morphology or fertility, except slightly smaller corolla size and seed weight in generations S_7_–S_10_ (data not shown). One of the S_11_ descendants was named Mx23Hm and subjected to genome sequencing, along with the heterozygous line 0431-1.

### 3.2. Genome sequencing and genome size estimation

The total numbers of Illumina reads obtained from Mx23Hm was 1,322,328,000, while that from 0431-1 was 2,698,276,042 (Supplementary Table S2). After trimming, 9–11% of PE reads and 38–45% of MP reads were excluded from further analysis. The total lengths of trimmed PE and MP reads in Mx23Hm were 64.5 and 33.9 Gb, respectively, and those in 0431-1 were 80.3 and 108.9 Gb, respectively.

Distributions of the number of distinct k-mers (*k* = 17) with the given multiplicity values reflected differences in heterozygosity between the two lines (Supplementary Fig. S2). One large and one very small peak were observed in Mx23Hm; the large peak with multiplicity of 105 was employed for genome size estimation. In contrast, two distinct peaks were observed in 0431-1; the higher peak with multiplicity of 62 was considered to represent heterozygous sequences, and the lower peak with multiplicity of 125 was employed for genome size estimation. The estimated genome sizes were 515.8 Mb (Mx23Hm) and 539.9 Mb (0431-1).

### 3.3. Assembly of the Mx23Hm and 0431-1 genomes

Assembly of the Illumina reads described in the Section 3.2 was carried out using the two computer programs based on different algorithms, SOAPdenovo2 r223 and Platanus ver. 1.2.1, as described in Materials and Methods. The results are summarized in Supplementary Table S3. The total lengths of the assembled sequences generated by SOAPdenovo2 r223 were longer than those generated by Platanus after gap closing (columns G, K versus O, S in Supplementary Table S3) and were closer to the estimated genome sizes described above (columns G, K, O and S in Supplementary Table S3). Considering that the purpose of this study is to survey as much genomic regions as possible to assign core candidates and line-specific sequences in the *I. trifida* genome, SOAPdenovo2 r223 was chosen for the following analyses.

The Mx23Hm PE reads, which had a total length of 64.5 Gb, were assembled by using SOAPdenovo2 r223 and GapCloser 1.10. The 740,762 generated scaffolds were subjected to further scaffolding by using SSPACE2.0 with the MP reads having expected insert sizes of 3 and 10 kb, followed by the exclusion of contaminated DNA sequences such as those derived from bacterial, fungal, and human genomes. The resultant number of scaffolds was 559,634, totalling 584.7 Mb in length. The sequences shorter than 300 bases were considered insignificant, because they were likely to be derived from repetitive or low-quality sequences, and therefore, such sequences were excluded from further analysis. The remaining 77,400 sequences were designated as ITR_r1.0, the total length of which was 512,990,885 bases, including 175,412,753 Ns. The GC% and N50 length were 35.6% and 42,586 bases, respectively (Table 1; Supplementary Table S3).

**Table 1.** Statistics of the assembled genome sequences for Mx23Hm and 0431-1

Sequenced line	Mx23Hm	0431-1
	(ITR_r1.0)	(ITRk_r1.0)
Number of sequences	77,400	181,194
Total length (bases)	512,990,885	712,155,587
Average length (bases)	6,628	3,930
Max length (bases)	910,847	1,352,076
Min length (bases)	300	300
N50 length (bases)	42,586	36,283
A	108,919,552	155,339,270
T	108,380,339	154,432,148
G	60,024,339	86,821,603
C	60,253,902	87,276,414
*N*	175,412,753	228,286,152
Total (ATGC)	337,578,132	483,869,435
GC% (GC/ATGC)	35.6	36.0

In 0431-1, a total of 1,578,945 scaffolds generated by the assembly of 80.3 Gb PE reads were subjected to further scaffolding with MP reads, generating 1,578,945 scaffolds (Supplementary Table S3). After the removal of contaminated sequences and sequences shorter than 300 bases, a total of 181,194 sequences comprising 712,155,587 bases with 228,286,152 Ns were finally obtained, which were collectively designated as ITRk_r1.0 (Table 1). The GC% and N50 length were 36.0% and 36,283 bases, respectively.

**Figure 1.**
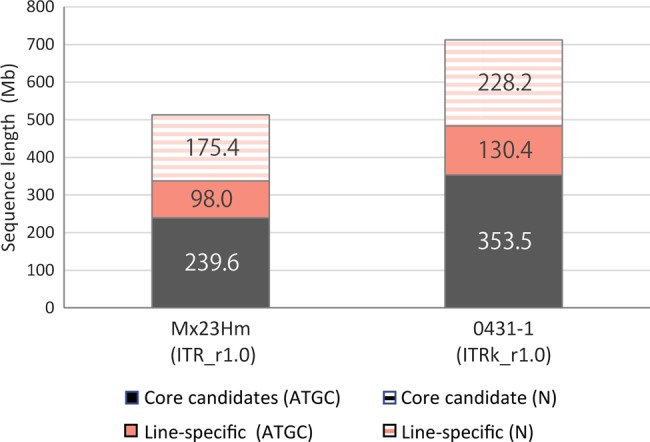
Total lengths of core candidates and line-specific sequences of ITR_r1.0 (Mx23Hm) and ITRk_r1.0 (0431-1).

The assembled sequences were then classified into ‘core candidates' and ‘line-specific’ sequences using the LAST program. The total lengths of core candidates were 239.6 Mb (Mx23Hm) and 353.5 Mb (0431-1), whereas those of line-specific sequences were 273.4 Mb (Mx23Hm) and 358.6 Mb (0431-1) (Fig. 1 and Supplementary Table S4). In both assembled sequences, most of the *N* bases were classified as line-specific sequences: the *N*% of Mx23Hm and 0431-1 was 64.2 and 63.6%, respectively.

Authenticity of the assembled genomic sequences was examined by comparison with the registered BAC contig sequences in NCBI derived from the genome of *I. trifida*. Six out of seven BAC contigs tested showed sequence similarity to the assembled Mx23Hm and 0431-1 genomic sequences (Supplementary Fig. S3). Despite some inconsistency possibly due to improper assembly or structural changes of the genomes among varieties, long stretches of linear dot plots were observed for these regions, indicating high degree of reliability of the assembled sequences. Multiple linear plots were often observed in 0431-1, which may reflect higher heterozygosity of the genome of this line.

### 3.4. Repetitive sequences

The total lengths of repetitive sequences were 142.7 Mb (Mx23Hm) and 230.8 Mb (0431-1) (Supplementary Table S5). Unique repeats were abundant in both genomes, constituting 76.0% (Mx23Hm) and 78.9% (0431-1) of all repeats. Of the known types of repeats, Class I LTR elements were observed most frequently, followed by low-complexity repeats. Percentages of the repetitive in the assembled sequences (excluding *N*) sequences accounted for 27.7 and 30.9% of the core candidate, and 80.6 and 96.9% of the line-specific sequences in Mx23Hm and 0431-1, respectively. The repeat masked sequences of the assembled genomic sequences were constructed and designated as ITR_r1.0_masked (Mx23Hm) and ITRk_r1.0_masked (0431-1). The numbers of total bases (A, T, G, and C) of the constructed repeat masked sequences were 192,898,192 bases (Mx23Hm) and 254,160,527 bases (0431-1, Supplementary Table S6). The numbers of total bases in core candidates were 155,039,354 bases (Mx23Hm) and 205,173,148 bases, while those in line specific were 37,858,838 bases (Mx23Hm) and 48,987,379 bases (0431-1).

The total numbers of SSRs identified in the assembled genome sequences were 108,397 (Mx23Hm) and 95,138 (0431-1) (Supplementary Table S7). The average frequency of SSRs was 20.9/100 kb in Mx23Hm, while that in 0431-1 was 13.1/100 kb. SSRs were more frequently observed in introns than in exons. Di-nucleotide motifs were more frequently observed than tri-nucleotide motifs in Mx23Hm (54,631 di-nucleotides and 34,619 tri-nucleotides), whereas a small difference was observed in the numbers of di- and tri-nucleotide motifs in 0431-1 (36,941 di-nucleotide and 35,489 tri-nucleotide).

### 3.5. RNA-encoding genes

A total of 2,089 and 3,168 putative genes for transfer RNAs (tRNAs) were identified in the assembled genomic sequences of Mx23Hm and 0431-1, respectively (Supplementary Table S8). Larger numbers of tRNA-encoding genes were identified in the core candidate sequences (1,335 in Mx23Hm and 2,006 in 0431-1) than in the line-specific sequences (754 in Mx23Hm and 1,162 in 0431-1). The total numbers of partial rRNA-encoding genes predicted in Mx23Hm and 0431-1 were 476 (5.8S: 12; 18S: 223; and 25S: 241) and 299 (5.8S: 16; 18S: 148; and 25S: 135), respectively (Supplementary Table S9). The numbers of rRNA-encoding genes identified in the core candidates (65 in Mx23Hm and 76 in 0431-1) were less than those in line-specific sequences (411 in Mx23Hm and 223 in 0431-1).

### 3.6. Prediction of protein-encoding genes and annotation

Two computer programs, Augustus and geneid, and two gene training sets, one derived from *A. thaliana* and the other from tomato, were used for *de novo* gene prediction. Augustus predicted 62,403 protein-encoding genes in the assembled genomic sequences of Mx23Hm using the *A. thaliana* training set, while geneid predicted 104,524 genes with the same training set (Supplementary Table S10). If only genes capable of encoding proteins of equal to or longer than 100 amino acid residues were taken into account, however, Augustus and geneid predicted similar number of genes, 50,689 and 50,515, respectively. When these two gene sets were compared, 26,364 genes (52.1% of the total predicted genes) were common to the both programs, and 1,274 (2.5%) and 1,956 (3.9%) genes were specific to Augustus and geneid, respectively, indicating that the two programs predicted similar sets of genes (Supplementary Fig. S4). Gene prediction by Augustus using the two different gene training sets resulted in generation of similar number of genes, 62,403 (*A. thaliana*) and 59,182 (tomato) (Supplementary Table S11). Putting these results together, we adopted the gene sets predicted by Augustus using the *A. thaliana* training set for further analyses.

An N50 length of the 62,403 protein-encoding genes predicted in Mx23Hm was 1,536 bases, and totalled 62,468,431 bases in length (Supplementary Table S12). A larger number of genes (109,449) was predicted in 0431-1 with a total length of 82,228,566 bases, and an N50 length was 1,230 bases. The predicted genes were classified as intrinsic genes or TEs (Supplementary Table S13); the percentages of intrinsic and partial genes (76%) and TEs (18–19%) were almost identical between the two assembled genomic sequences. The ratio of full-length intrinsic genes was higher in Mx23Hm, reflecting the higher quality of the assembled sequences. The numbers of the sum of intrinsic and partial genes were 47,511 with a total length of 44,591,152 bases for Mx23Hm, while that for 0431-1 was 83,366 with a total length of 58,192,632 bases (Supplementary Table S12). Sets of intrinsic and partial genes were designated as ITR_r1.0_cds_ip (Mx23Hm) and ITRk_r1.0_cds_ip (0431-1). The total numbers of predicted genes in core candidate and line-specific sequences were 11,823 and 50,580, respectively, in Mx23Hm, and 28,831 and 80,618, respectively, in 0431-1 (Supplementary Table S12). The percentages of intrinsic and partial genes in core candidate sequences (82–83%) were slightly higher than that in line-specific sequences (74–75%) (Supplementary Table S13).

**Figure 2.**
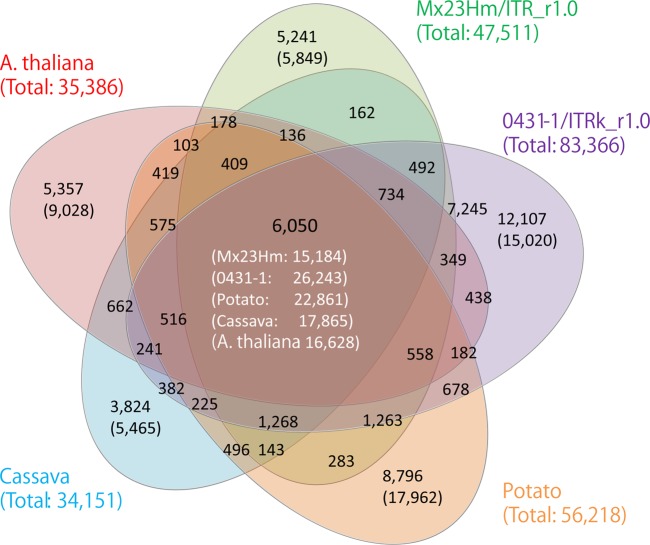
Venn diagram showing the numbers of gene clusters in *Ipomoea trifida* and other plant species, i.e. *Arabidopsis thaliana*, potato (*Solanum tuberosum*), and cassava (*Manihot esculenta*). The black and white numbers in parenthesis represent the numbers of non-clustered sequences and sequences clustered with other species, respectively. The green, purple, orange, aqua, and red numbers in parenthesis represent the total numbers of putative genes subjected to clustering.

The intrinsic and partial genes were subjected to clustering with a total of 57,354 predicted genes containing 35,386 genes in *A. thaliana* as a dicot model and 56,218 genes in potato and 34,151 genes in cassava as tuber crops by similarity searches using the CD-hit program. Of the putative *I. trifida* genes, 15,184 (Mx23Hm) and 26,243 (0431-1) could be clustered with predicted genes identified in other species, whereas the remaining 5,849 (Mx23Hm) and 15,020 (0431-1) were not clustered and therefore considered as *I. trifida*-specific genes (Fig. 2; Supplementary Tables S14 and S15).

The intrinsic genes were classified based on GO slim analysis together with those of *A. thaliana*, potato, and cassava. The numbers of genes classified into the molecular function (MF), cellular component (CC), and biological process (BP) categories are summarized in Supplementary Fig. S5. In parallel, a total of 28,208 (Mx23Hm) and 49,610 (0431-1) putative genes were classified into KOG functional categories along with the predicted genes of *A. thaliana* (33,630 genes), potato (41,952 genes), and cassava (31,668 genes) (Supplementary Fig. S6). In the ‘Metabolism’ category, the number of classified genes in 0431-1 was higher than that in Mx23Hm.

Mapping of the genes for intrinsic proteins in *I. trifida* onto the KEGG metabolic pathways was carried out along with mapping of the genes in other plant species, including *A. thaliana* (13,154 genes), potato (14,318 genes), and cassava (14,251 genes). In the pathways categorized ‘1. Metabolism’ in the KEGG database, a total of 2,794 (Mx23Hm) and 2,444 (0431-1) putative genes were mapped onto 1,750 and 1,547 enzymes listed in the KEGG GENES database, which were involved in 138 and 137 metabolic pathways, respectively (Supplementary Table S16). The pathways present only in Mx23Hm in *I. trifida* were ‘mucin type O-glycan biosynthesis' and ‘non-ribosomal peptide structures', whereas ‘biosynthesis of unsaturated fatty acids' was present only in 0431-1.

### 3.7. Genes involved in starch biosynthesis

Starch biosynthesis is catalyzed by several enzymes including ADP-glucose pyrophosphorylase (AGPase), starch synthase (SS), branching enzyme (BE), and isoamylase (ISA). Similarity searches and phylogenetic analyses of genes encoding these enzymes in ITR_r1.0 (Mx23Hm) identified several potential homologues, which are summarized in Supplementary Table S17. For AGPase, seven and three homologues potentially encoding the large and the small subunits, respectively, were detected in Mx23Hm. Two homologues for the large subunit were located at the ends of scaffolds and were likely to be partial. For SS, nine putative homologues were found in Mx23Hm. Phylogenetic analysis using the known isoforms of plant SS suggested that Itr_sc000002.1_g00061.1 and Itr_sc000727.1_g00009.1 are homologues of the genes for soluble *starch synthase II* (*SS II*) (Supplementary Fig. S7). For BE and ISA, three homologues of each were detected in Mx23Hm.

### 3.8. SNP discovery and CNV analysis

Of the 596 M (Mx23Hm) and 484 M (0431-1) trimmed PE reads, 88.8 and 65.7%, respectively, were successfully mapped onto the assembled genome of Mx23Hm (ITR_r1.0). The mean depth of the mapped reads was 103.2 in Mx23Hm, covering 66.1% of the assembled genome, while the read depth in 0431-1 was 62.0, covering 60.8% of the assembled genome. Inspection of the sequence alignments in both lines detected a total of 7,970,056 variant candidates among the two lines. Of these candidates, 5,086,488 loci were selected according to Criteria 1 and 2 in the Materials and Methods. Further selection by application of Criterion 3 resulted in 2,089,076 candidates as a high-quality SNP set. Finally, 1,464,173 loci, including 596,211 heterozygotes and 867,962 homozygotes, were selected as highly confident SNP candidates by removing the repetitive sequences (Criterion 4). The SNP density was 1 SNP/231 bases in ITR_r1.0 (Ns were excluded), and the transition/transversion ratio was 1.45 (865,368 transitions and 598,805 transversions).

The effects of SNPs on gene functions were predicted using SnpEff, which groups SNPs into four categories (high-impact, moderate, modifier, and low) based on the positions of SNPs in the genome sequences. Among the 1,464,173 SNP candidates, 3,894 (0.3%) were classified as high-impact SNPs, including ‘splice acceptor and donor variants', ‘loss of the start codon’, or ‘gain/loss of the stop codon’. Another 137,786 loci (9.4%) were classified as ‘moderate effects' (missense variants), 1,037,197 (70.8%) as ‘modifiers' (e.g. variants in intergenic regions and intron), and 257,795 (17.6%) as ‘low-impact’ (e.g. synonymous variants) (Supplementary Fig. S8). The remaining 27,501 loci (1.9%) were not assigned to any categories.

**Figure 3.**
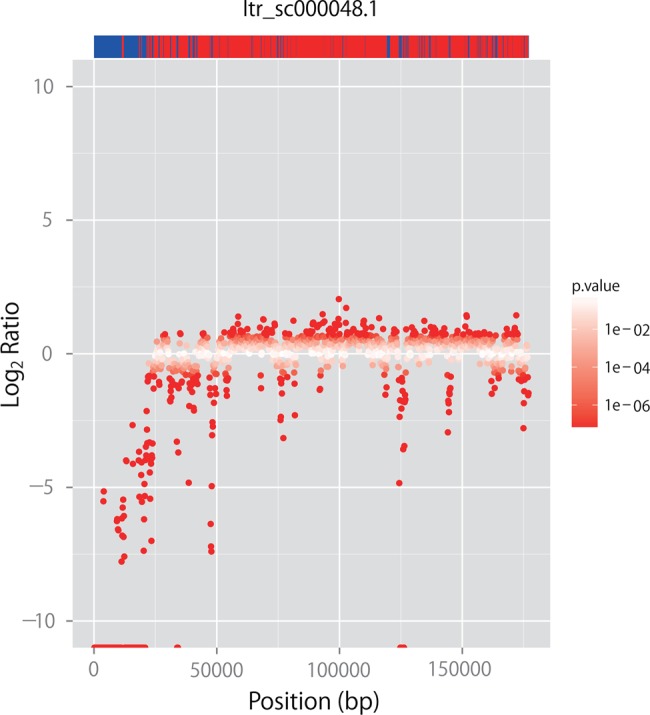
CNV distributions and corresponding positions of core candidates and line-specific sequences of scaffold Itr_sc000048.1 (1,177,009 bases in length). Red dots show the log_2_ ratios of CNVs between Mx23Hm and 0431-1. The upper bar represents core candidate (red) and line-specific (blue) sequences in their corresponding positions.

CNVs in the genomes of Mx23Hm and 0431-1 were detected using the CNV-seq program. CNVs between Mx23Hm and 0431-1 longer than 50 kb in length were identified on 2,095 scaffolds. The numbers of detected CNVs varied in accordance with the threshold values of the log_2_ ratio (log-transformed ratio of the number of mapped reads in 0431-1 to the number in Mx23Hm) and CNV sizes. For example, 54 CNVs with a mean length of 12,444 bases were identified under the stringent condition (log_2_ ratio >10 and CNV size >10,000 bases) (Supplementary Fig. S9). Under a more relaxed condition (i.e. log_2_ ratio of >1 and CNV size >1,000 bases), 16,682 CNVs with a mean length of 1,918 bases were detected. Under both stringent and relaxed conditions, the CNVs occupied 671,974 bases (0.3%) and 44,892,563 bases (18.8%), respectively, of the total length of the 2,095 contigs (239,146,348 bases). The mean absolute values of log_2_ ratios in the core candidate and line-specific sequences were 0.63 and 1.72, respectively, indicating that CNVs were significantly enriched in the line-specific regions relative to the core candidate regions of the genomes (*P* < 0.001) (Fig. 3; Supplementary Fig. S10).

## 4. Discussion

The genome size of *I. trifida* was estimated to be 515.8–539.9 Mb by analysis of the multiplicity of k-mers of the Illumina sequencing reads. This size was smaller than the predicted values of 737–814 Mb, which were based on a previous report of DNA content in the hexaploid sweet potato (4.8–5.3 pg/2C).^2^ The k-mer analysis also supported that 0431-1 is highly heterozygous, whereas Mx23Hm is homozygous, which is consistent with the results of polymorphic analysis using 14 SSR markers.

The total length of ITR_r1.0 (513 Mb) for Mx23Hm corresponded well to the genome size estimated by multiplicity of k-mers, whereas the total length of ITRk_r1.0 (712 Mb) for 0431-1 was larger than the estimated value, probably due to unassembled heterozygous sequences. Moreover, the N50 of ITR_r1.0 was longer than that of ITRk_r1.0 despite the fact that fewer MP reads were used. These results may reflect the difficulty in sequence assembly of heterozygous genomes. The N50 value of 36.3–42.6 kb of the assembled sequences in this study is long enough to serve as a basis for a primary survey of the *I. trifida* genome. However, additional effort would be necessary to reduce the proportion of the *N* base, which currently occupies 34% (Mx23Hm) and 32% (0431-1) of the assembled genomic sequences.

When the *N* bases were excluded from the assembled sequences, the content of the repetitive sequences was calculated to be 42.3–47.7% of the entire *I. trifida* genomes, which is lower than the contents in other plant species such as *Lotus japonicus* (56.8%),^58^ potato (64.2%),^59^ and tomato (68.3%).^60^ While the length of the assembled sequences of 0431-1 (712 Mb) was longer than that of Mx23Hm (513 Mb), the number of di-nucleotide SSRs of 0431-1 (36,941) was, for example, fewer than that of Mx23Hm (54,631). The distribution of numbers of repetitions indicated that the lengths of the SSRs identified in 0431-1 were shorter than those in Mx23Hm (Supplementary Fig. S11). It is likely that the shorter lengths of the scaffolds in 0431-1 hampered the success of detection of SSRs with long lengths.

The assembled sequences were classified into two categories, ‘core candidate’ and ‘line-specific’ sequences. In this study, we expected to investigate the genome structures of three haplotypes of *I. trifida*, one from Mx23Hm and two from the highly heterozygous 0431-1, to identify sequence variations. We adopted the term ‘core candidate’ rather than ‘core’ to refer to the conserved regions in the genomes, because three haplotypes may not be sufficient. The total length of core candidate sequences in 0431-1 was longer than those in Mx23Hm. Multiple core sequences in 0431-1 corresponded to single sequences in Mx23Hm (Supplementary Fig. S12), suggesting that the longer core candidate sequences in 0431-1 reflected the high heterozygosity in the 0431-1 genome. The total lengths of line-specific sequences were 273.4 Mb in Mx23Hm and 358.7 Mb in 0431-1, and 64% of these sequences were occupied by the *N* bases. When these Ns were excluded from the calculation, the ratios of the line-specific sequences to the whole-genome sequences fell to 29% in Mx23Hm and 27% in 0431-1, being rich in repetitive sequences and rRNA-encoding genes (Supplementary Tables S5 and S9).

A total of 62,403 and 109,449 protein-encoding genes were predicted in the assembled genomic sequences of Mx23Hm and 0431-1, respectively. It is likely that the larger number of predicted genes in 0431-1 might reflect the high heterozygosity of this line. We focussed on genes involved in starch biosynthesis, namely AGPase, BE, ISA, and SS that catalyze starch biosynthesis, because starch is a nutritionally and industrially important component in the storage roots of sweet potato. AGPase catalyzes the conversion of glucose 1-phosphate to ADP-glucose, which is subsequently used by SS as a substrate for elongation of the α-1,4 glucan chain. BE and ISA play roles in amylopectin biosynthesis though the generation (BE) or degradation (ISA) of α-1,6 branches. For AGPase, a total of 10 homologues were identified in ITR_r1.0. Of these homologues, two of the large subunit were located on the ends of scaffolds and were likely to be partial. For BE and ISA, three homologues of each were detected in ITR_r1.0. These include the sequences for isoforms that have not been reported from sweet potato so far. Phylogenetic analysis suggested that two homologues (Itr_sc000002.1_g00061.1 and Itr_sc000727.1_g00009.1) of *SSII* genes are present in *I. trifida*, in contrast to potato and *Arabidopsis*, in which only a single *SSII* gene is present. Of these, Itr_sc000727.1_g00009.1 is likely to be an orthologue of the previously identified *SSII* gene of sweet potato.^61^ In addition, a highly similar sequence of Itr_sc000002.1_g00061.1 was found in the NCBI Transcriptome Shotgun Assembly (TSA) of sweet potato, suggesting the presence of an orthologue in the sweet potato genome. The three *SSII* isoforms in rice have different tissue specificity.^62^ Therefore, it is possible that the two homologues of *SSII* in *I. trifida* and sweet potato have distinct functions in different tissues.

Genomic variation can be surveyed by SNP and CNV analyses. Among three haplotypes in the two assembled genomes, we discovered a total of 1,464,173 SNP candidates. Of these SNPs, 596,211 were heterozygotes while 867,962 were homozygotes in the 0431-1 genome, suggesting that 59.3% (867,962/1,464,173) of SNP alleles were homozygotes in the heterozygous line. When the Ns were excluded from the estimation, the mean frequencies of SNPs between the two assembled genomes were 1 SNP per 231 bases (Mx23Hm) and 331 bases (0431-1). Of these SNPs, 3,894 were predicted to have high impacts on gene function. In particular, one, two, and one high-impact SNPs were predicted in the putative genes encoding AGPase, SS, and BE, respectively (Supplementary Table S17). CNVs were discovered in from 0.3 to 18.8% of the genome regions, depending on the different thresholds. Though the number of CNVs was less than that of SNPs, the discovered CNVs may have greater potential to disrupt gene functions due to gene deletions, frame-shift mutations, and alternating gene expressions.

This is the first report of a whole-genome survey for a plant belonging to the genus *Ipomoea*. Within this genus, sweet potato is the most important species in terms of industrial applicability. However, genome analysis of this species has been hindered by its hexaploid characteristics. The sequence information of the diploid genome would therefore be a useful resource for investigation of the genomes of related polyploid species. Whole-genome sequences of the closest wild relatives have been performed prior to genomic characterization of several important polyploid crop species such as cotton,^63^ banana,^64^ and strawberry.^65^ These studies suggested that the construction of pseudomolecules along individual chromosomes of the diploid genomes would be necessary to fully utilize the obtained information. For this purpose, development of high-density linkage maps of the *I. trifida* genome is the next crucial step.

## 5. Data availability

The genome assembly data, annotations, gene models, and SNPs of *I. trifida* are available at the Sweetpotato GARDEN (http://sweetpotato-garden.kazusa.or.jp). The BioProject accession number of *I. trifida* is PRJDB3230. The WGS (CON) accession numbers of assembled sequences in Mx23Hm and 0431-1 are BBOG01000001-BBOG01163047 (DF850533-DF884990) and BBOH01000001-BBOH01377770 (DF884991-DF933566), respectively. The genome sequence reads obtained by Illumina HiSeq 2000 are available from the DDBJ Sequence Read Archive (DRA) under the accession numbers DRR023905-DRR023907 (Mx23Hm) and DRR023898-DRR023904 (0431-1).

## Supplementary data

Supplementary data are available at www.dnaresearch.oxfordjournals.org.

## Funding

The work was supported by the Kazusa DNA Research Institute Foundation and National Agriculture and Food Research Organization. Funding to pay the Open Access publication charges for this article was provided by the Kazusa DNA Research Institute.

## Supplementary Material

Supplementary Data
