# Supplementary material for: Survey of genome sequences in a wild sweet potato, Ipomoea trifida (H. B. K.) G. Don
Source: DNA Res. 2015 Mar 24;22(2):171–9. doi: 10.1093/dnares/dsv002 (PMC4401327; doi:10.1093/dnares/dsv002)
Supplement: Supplementary Data [file supp_22_2_171__index.html]

Survey of genome sequences in a wild sweet potato, Ipomoea trifida (H. B. K.) G. Don — Survey of genome sequences in a wild sweet potato, Ipomoea trifida (H. B. K.) G. Don — Supplementary Data 

# Survey of genome sequences in a wild sweet potato, *Ipomoea trifida* (H. B. K.) G. Don

## Supplementary Data

Supplementary Data

**Files in this Data Supplement:**

- Supplementary Figure 10 - pdf file
- Supplementary Figures - pptx file
- Supplementary Tables - xlsx file
