# Supplementary material for: Survey of genome sequences in a wild sweet potato, Ipomoea trifida (H. B. K.) G. Don
Source: DNA Res. 2015 Mar 24;22(2):171–9. doi: 10.1093/dnares/dsv002 (PMC4401327; doi:10.1093/dnares/dsv002)
Supplement: Supplementary Data [file supp_dsv002_dsv002supp_fig10.pdf]

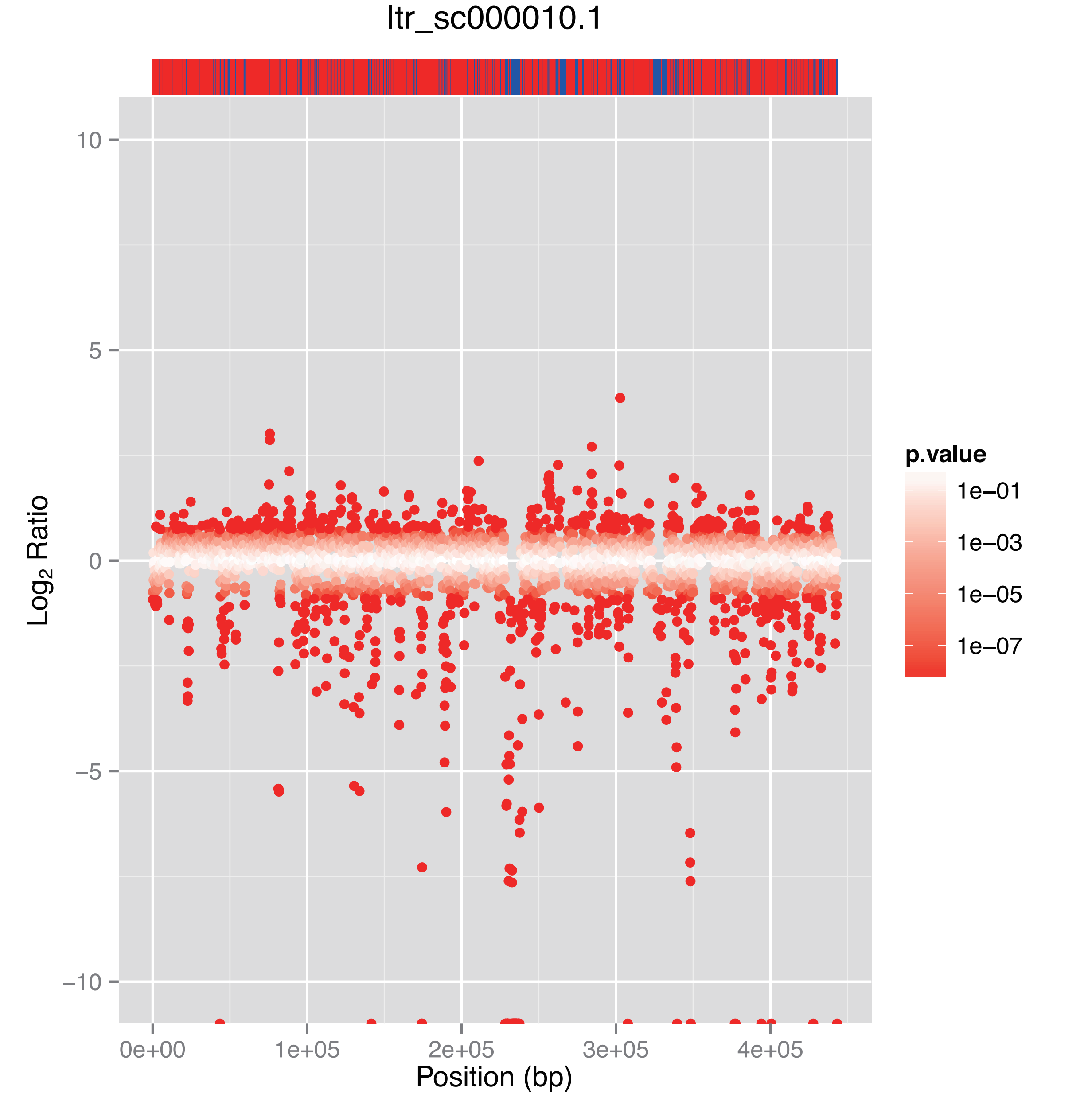

ltr_sc000010.1
10
5
0
−5
−10
Log2 Ratio
0e+00
1e+05
2e+05
3e+05
4e+05
Position (bp)
p.value
1e−01
1e−03
1e−05
1e−07

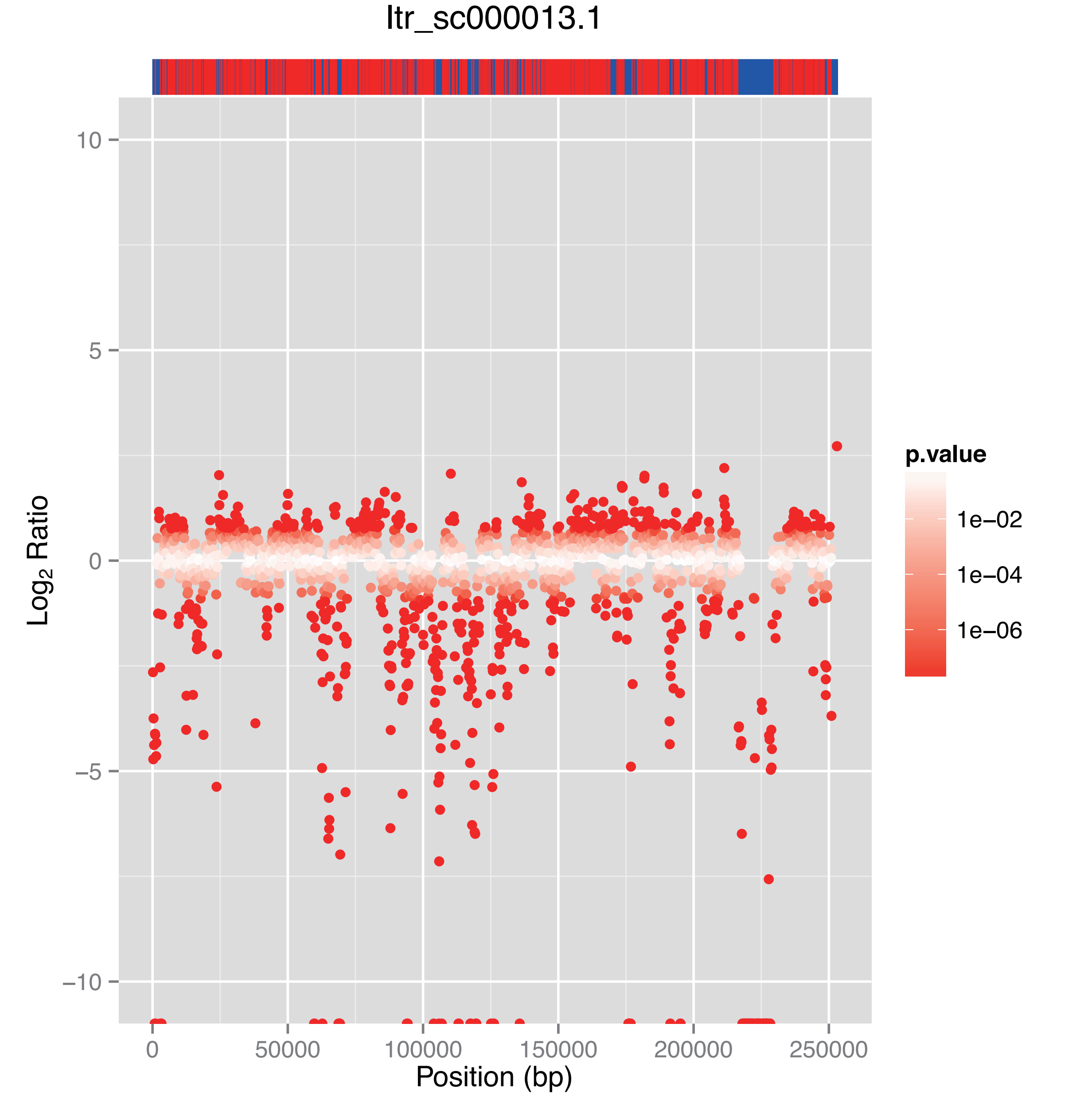

ltr_sc000013.1
10
5
0
−5
−10
Log2 Ratio
0
50000
100000
150000
200000
250000
Position (bp)
p.value
1e−02
1e−04
1e−06

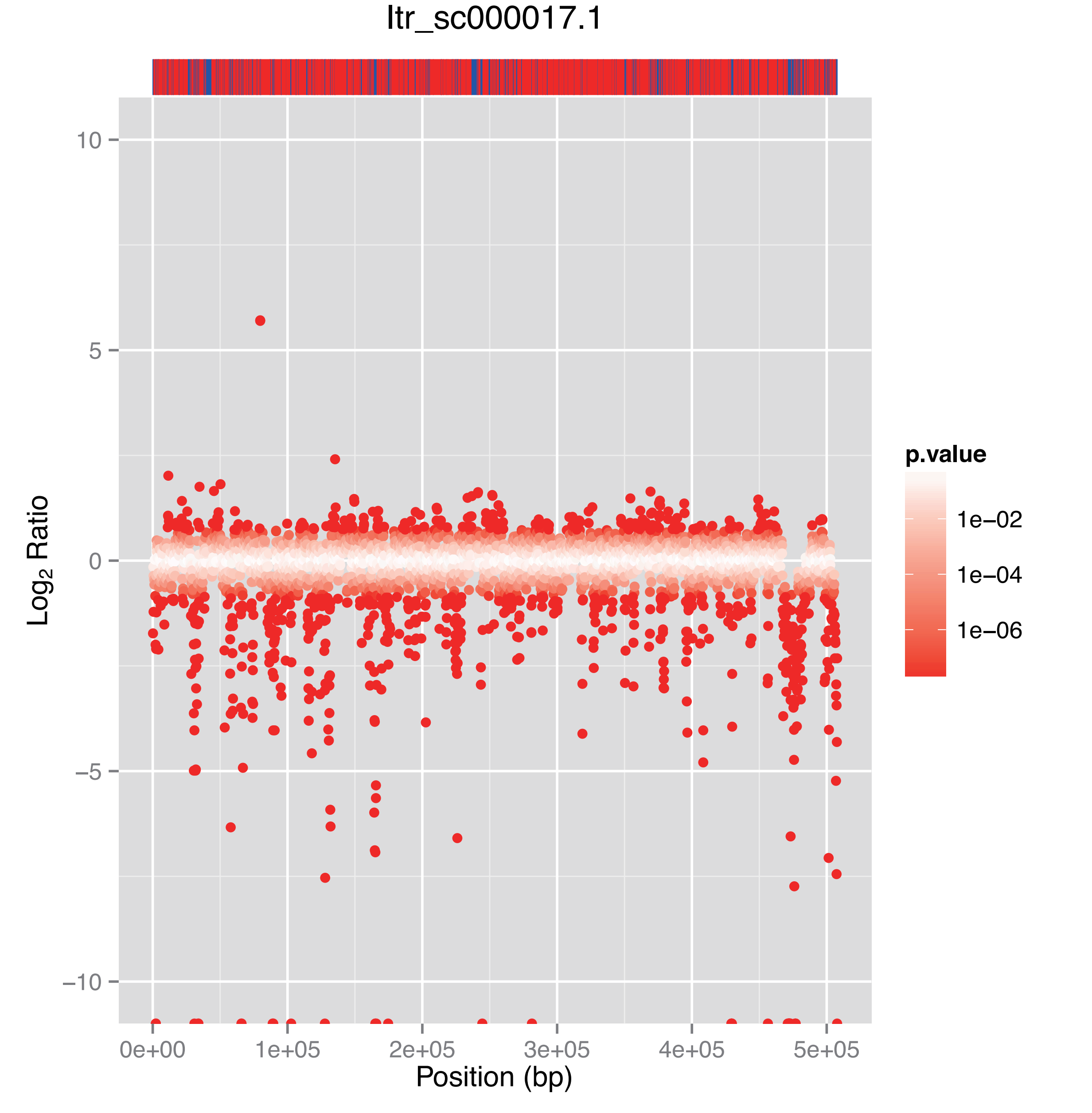

ltr_sc000017.1
10
5
0
−5
−10
Log2 Ratio
0e+00
1e+05
2e+05
3e+05
4e+05
5e+05
Position (bp)
p.value
1e−02
1e−04
1e−06

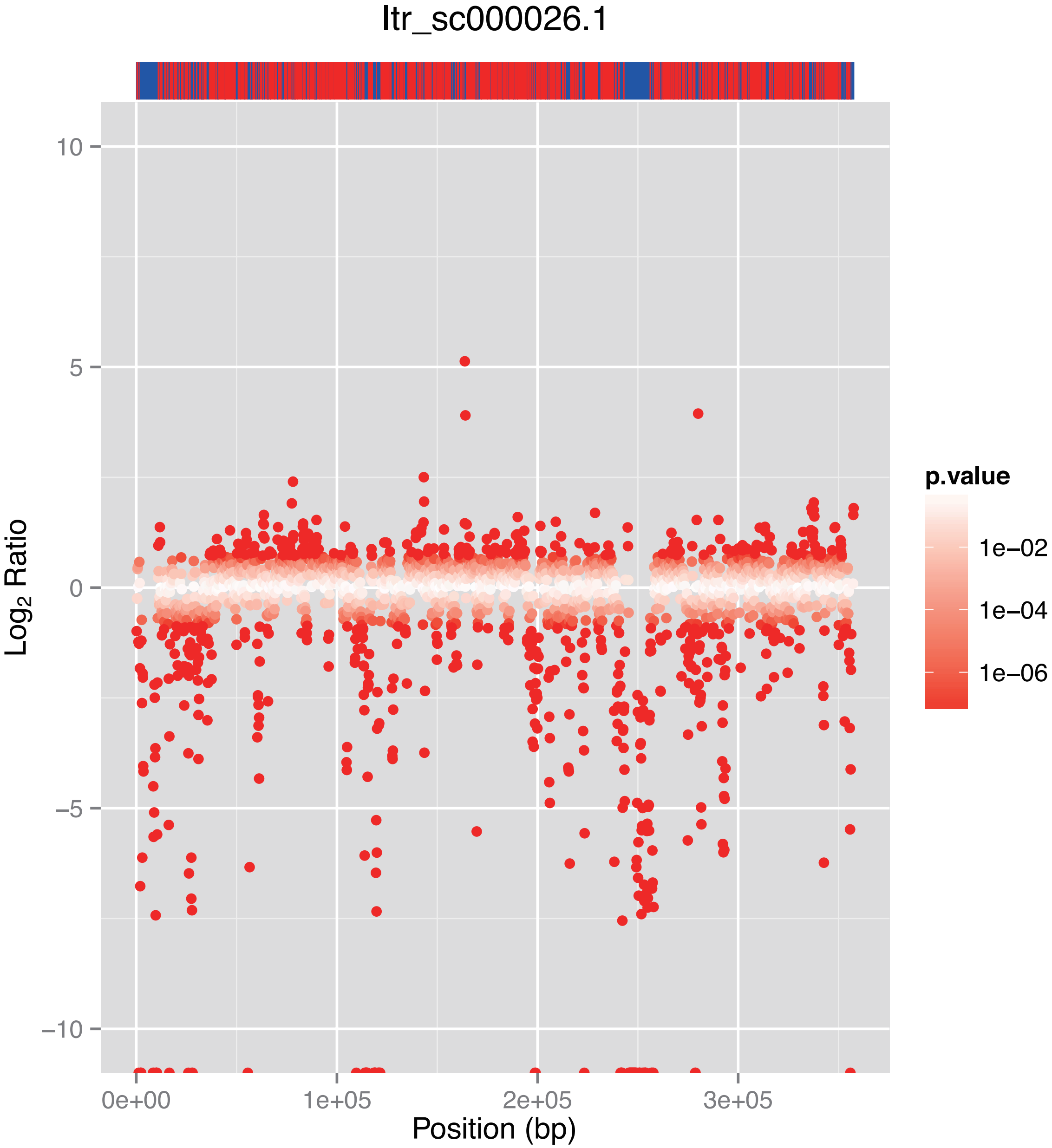

ltr_sc000026.1
10
5
0
−5
−10
Log2 Ratio
0e+00
1e+05
2e+05
3e+05
Position (bp)
p.value
1e−02
1e−04
1e−06

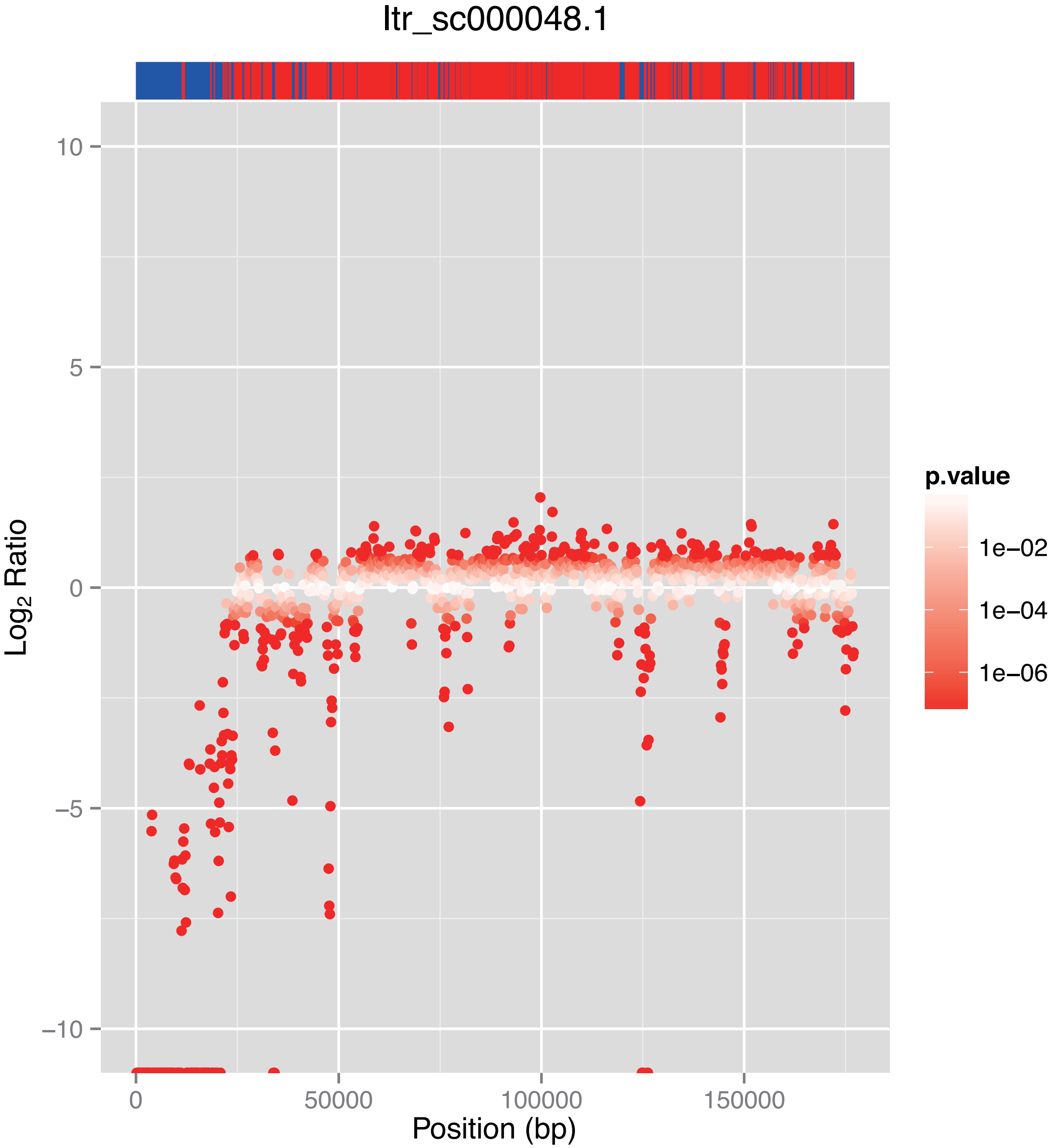
ltr_sc000048.1
10
5
0
−5
−10
Log2 Ratio
0
50000
100000
150000
Position (bp)
p.value
1e−02
1e−04
1e−06

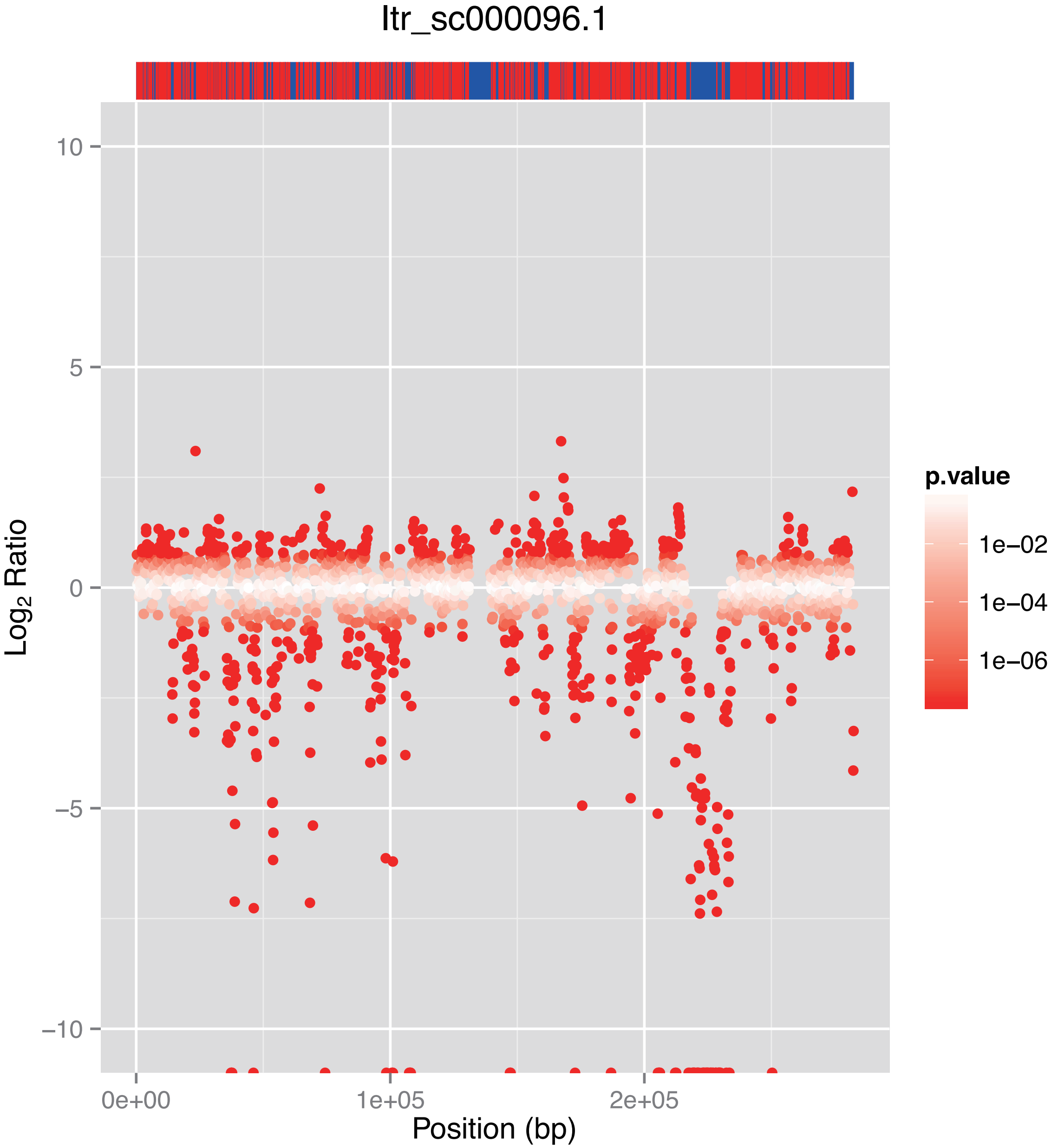

ltr_sc000096.1
10
5
0
−5
−10
Log2 Ratio
0e+00
1e+05
2e+05
Position (bp)
p.value
1e−02
1e−04
1e−06

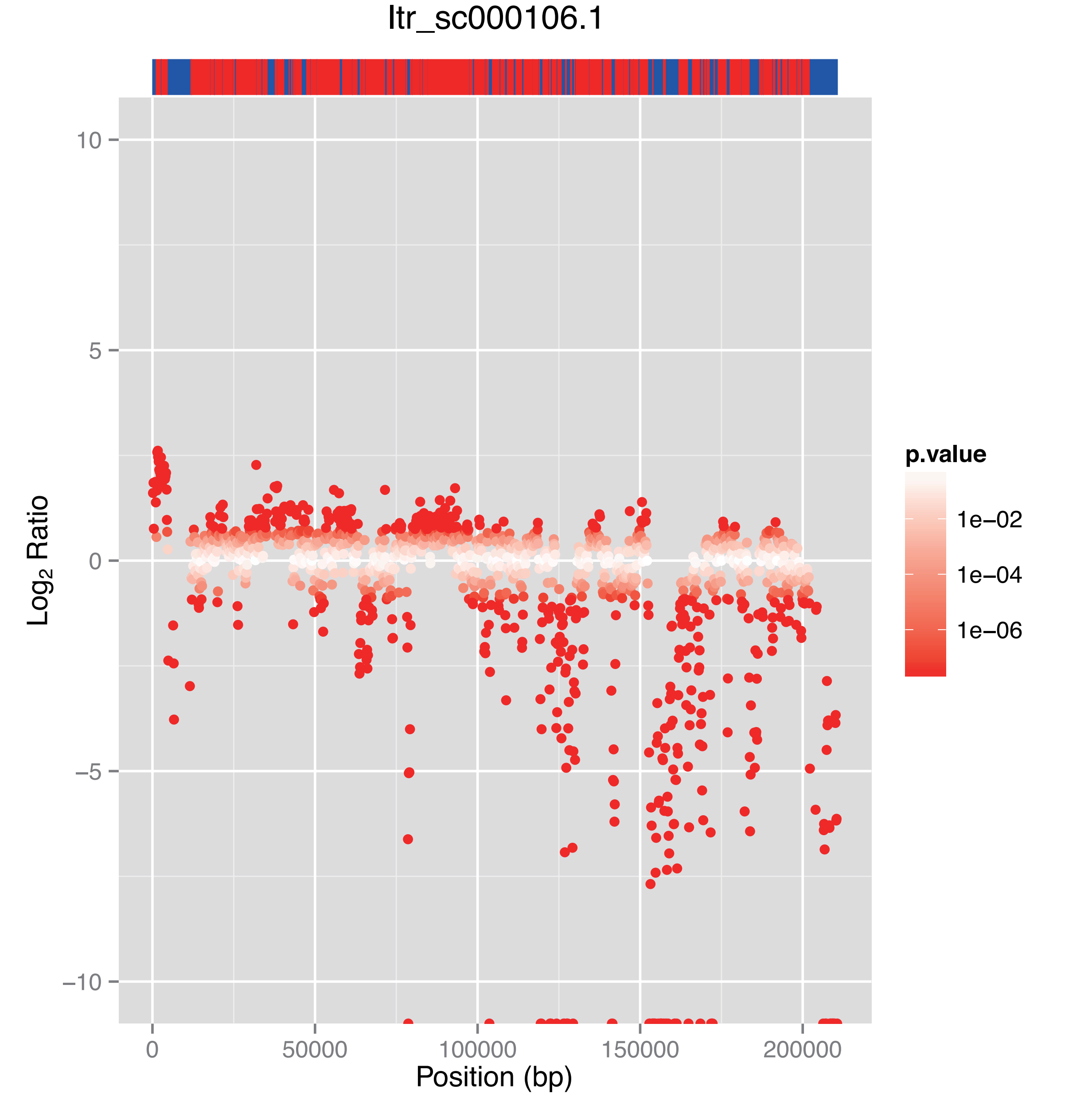

ltr_sc000106.1
Log2 Ratio
10
5
0
−5
−10
0
50000
100000
150000
200000
Position (bp)
p.value
1e−02
1e−04
1e−06

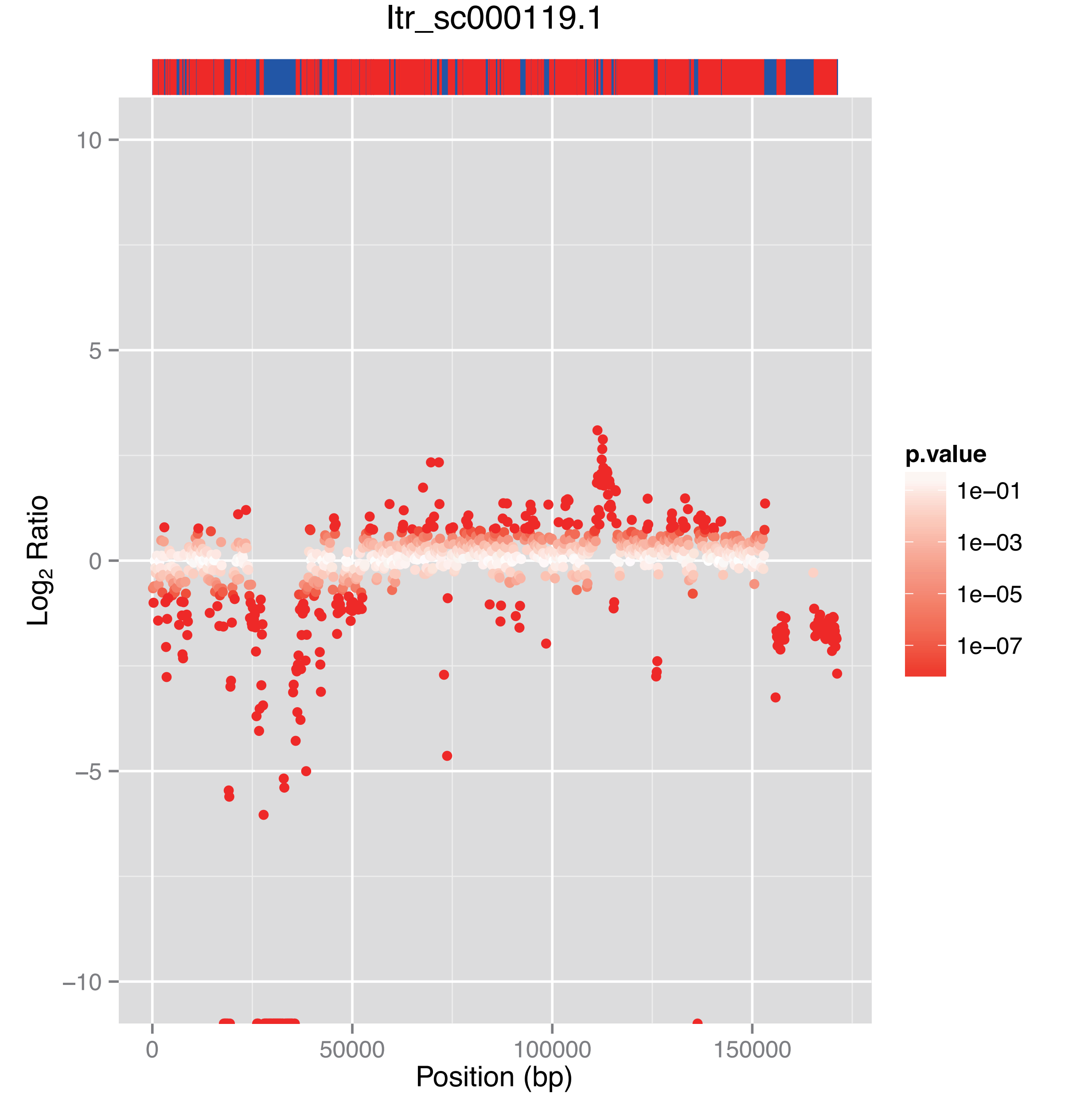

ltr_sc000119.1
Log2 Ratio
10
5
0
−5
−10
0
50000
100000
150000
Position (bp)
p.value
1e−01
1e−03
1e−05
1e−07

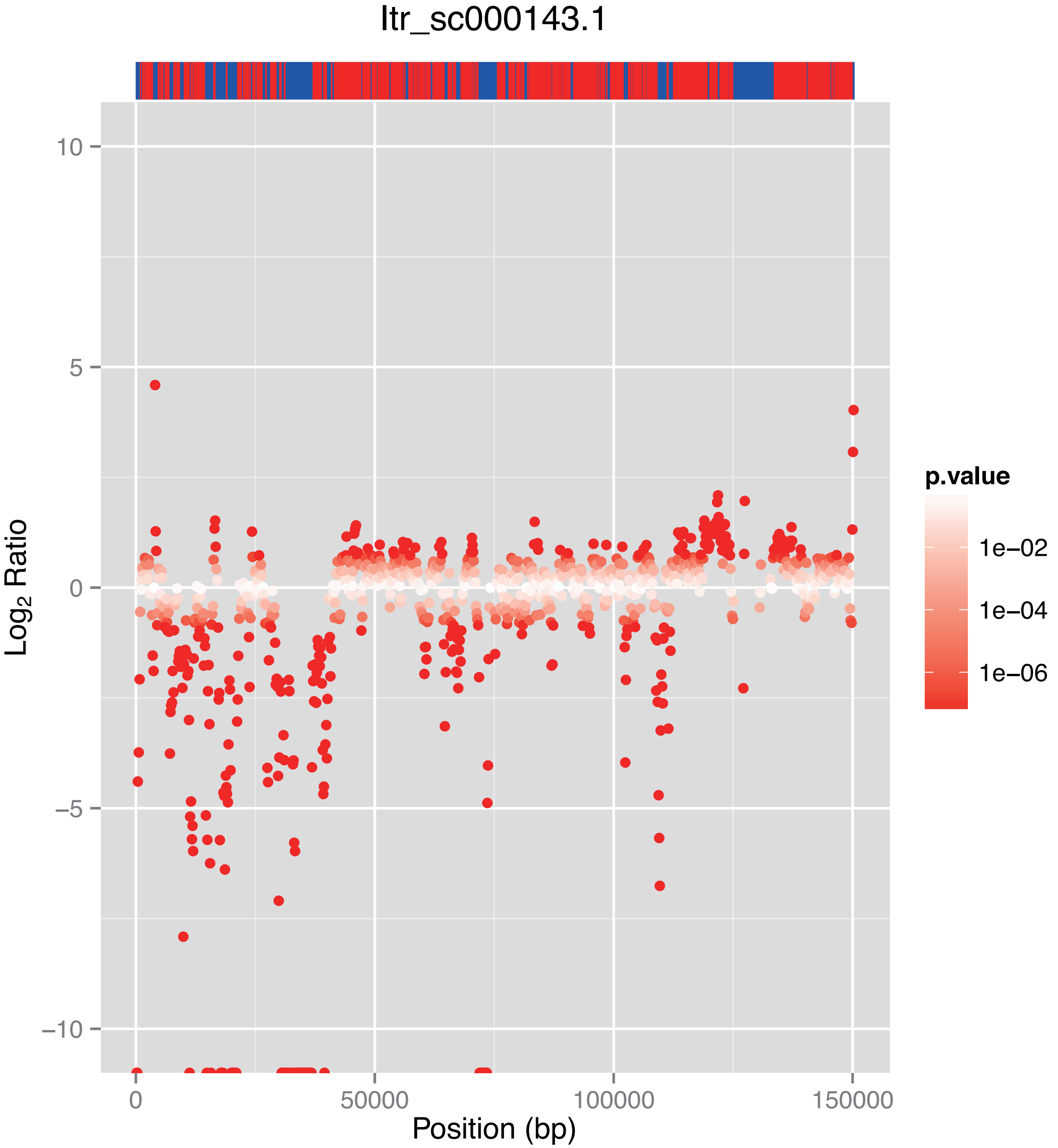

ltr_sc000143.1
Log2 Ratio
10
5
0
−5
−10
0
50000
100000
150000
Position (bp)
p.value
1e−02
1e−04
1e−06

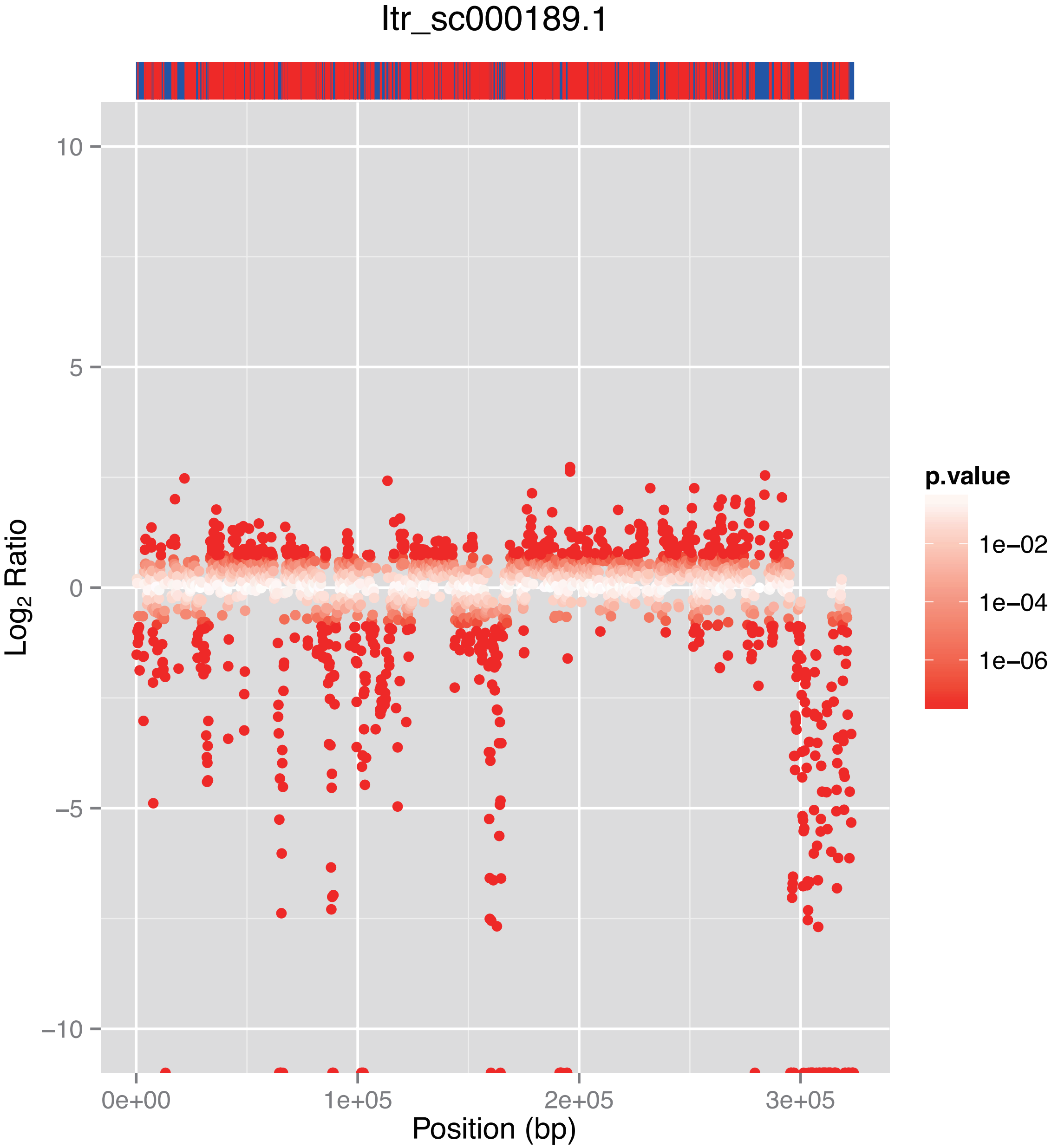
ltr_sc000189.1
10
5
0
−5
−10
Log2 Ratio
0e+00
1e+05
2e+05
3e+05
Position (bp)
p.value
1e−02
1e−04
1e−06

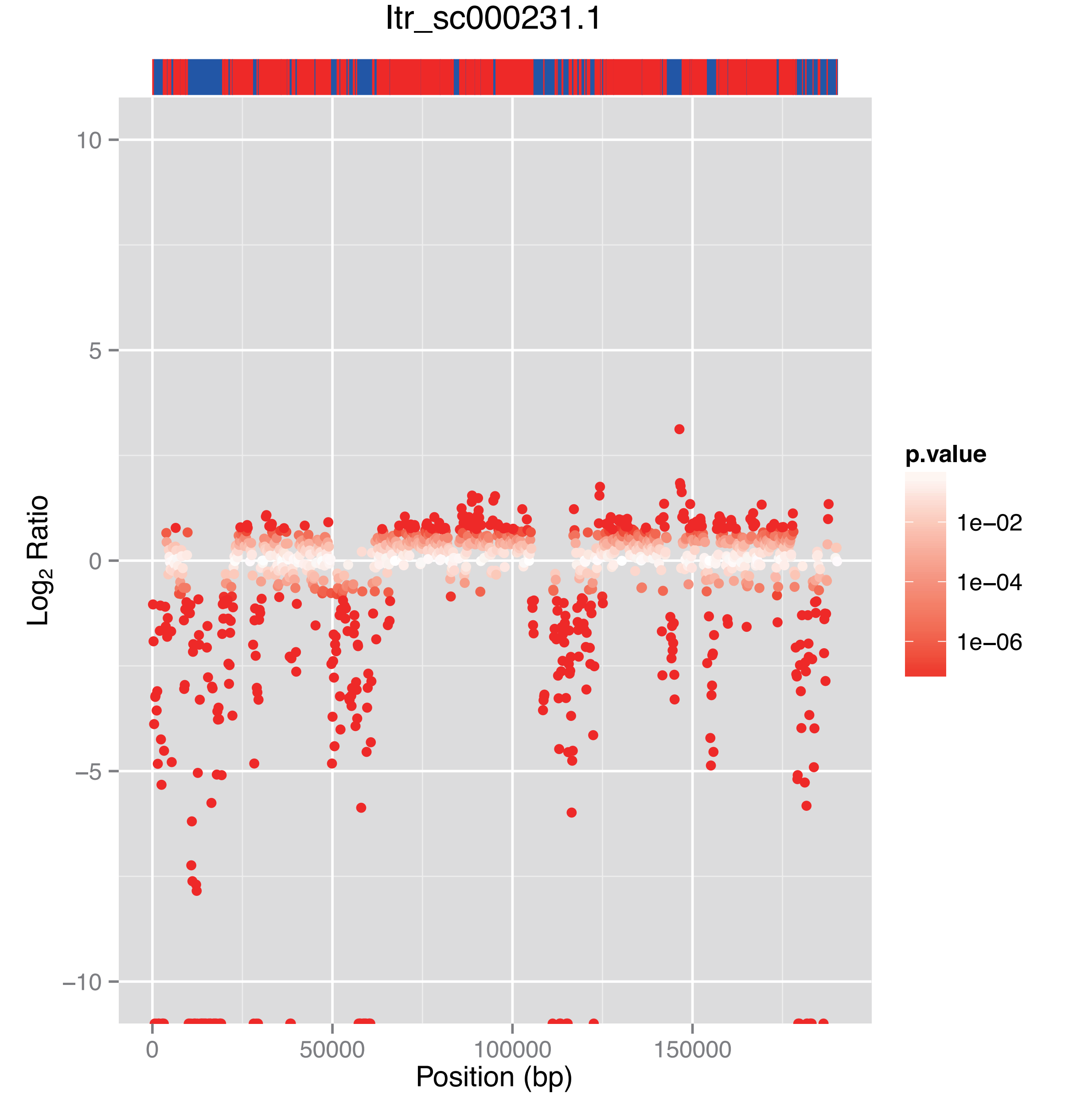
ltr_sc000231.1
10
5
0
−5
−10
Log2 Ratio
0
50000
100000
150000
Position (bp)
p.value
1e−02
1e−04
1e−06

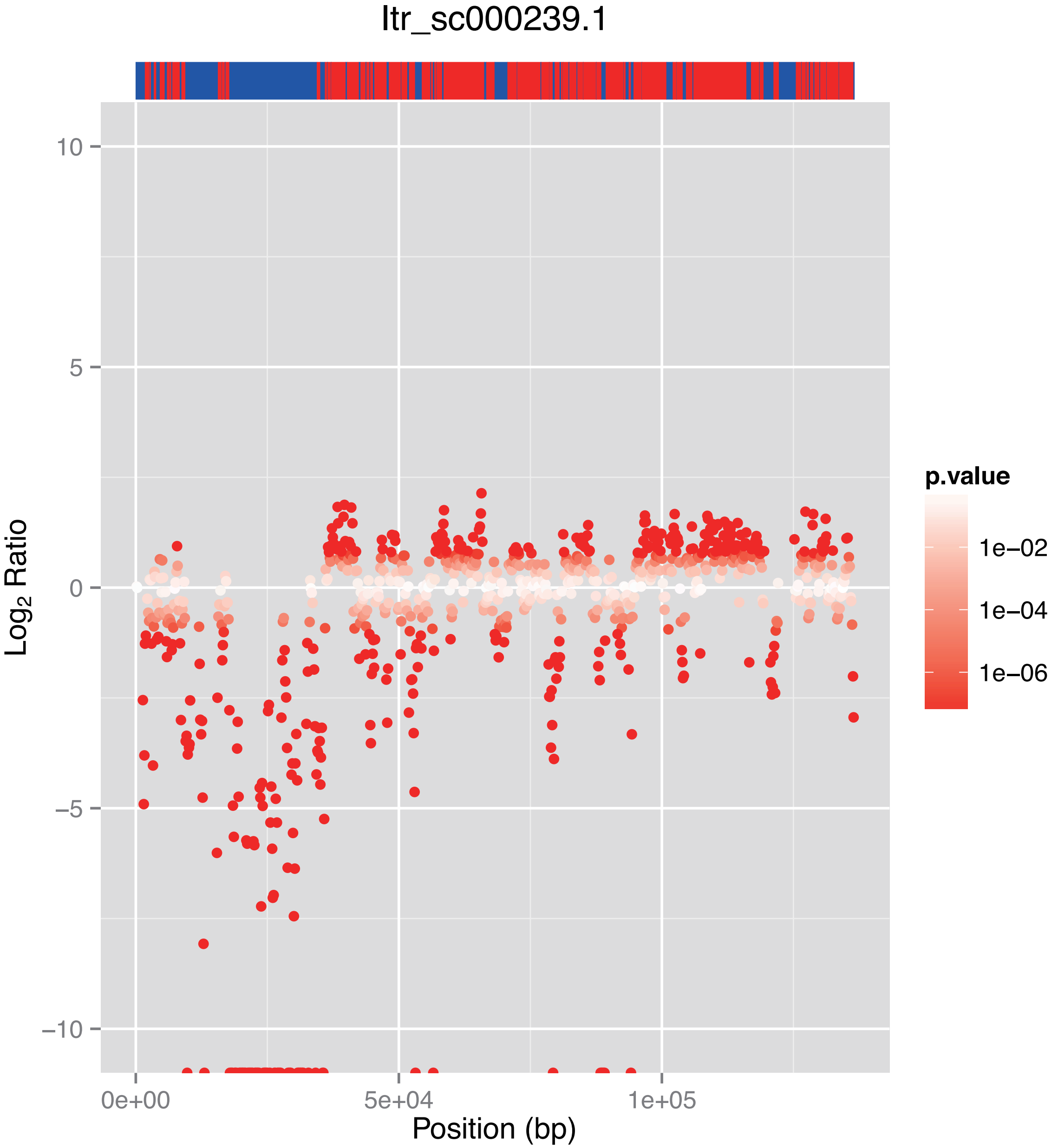
ltr_sc000239.1
10
5
0
−5
−10
Log2 Ratio
0e+00
5e+04
1e+05
Position (bp)
p.value
1e−02
1e−04
1e−06

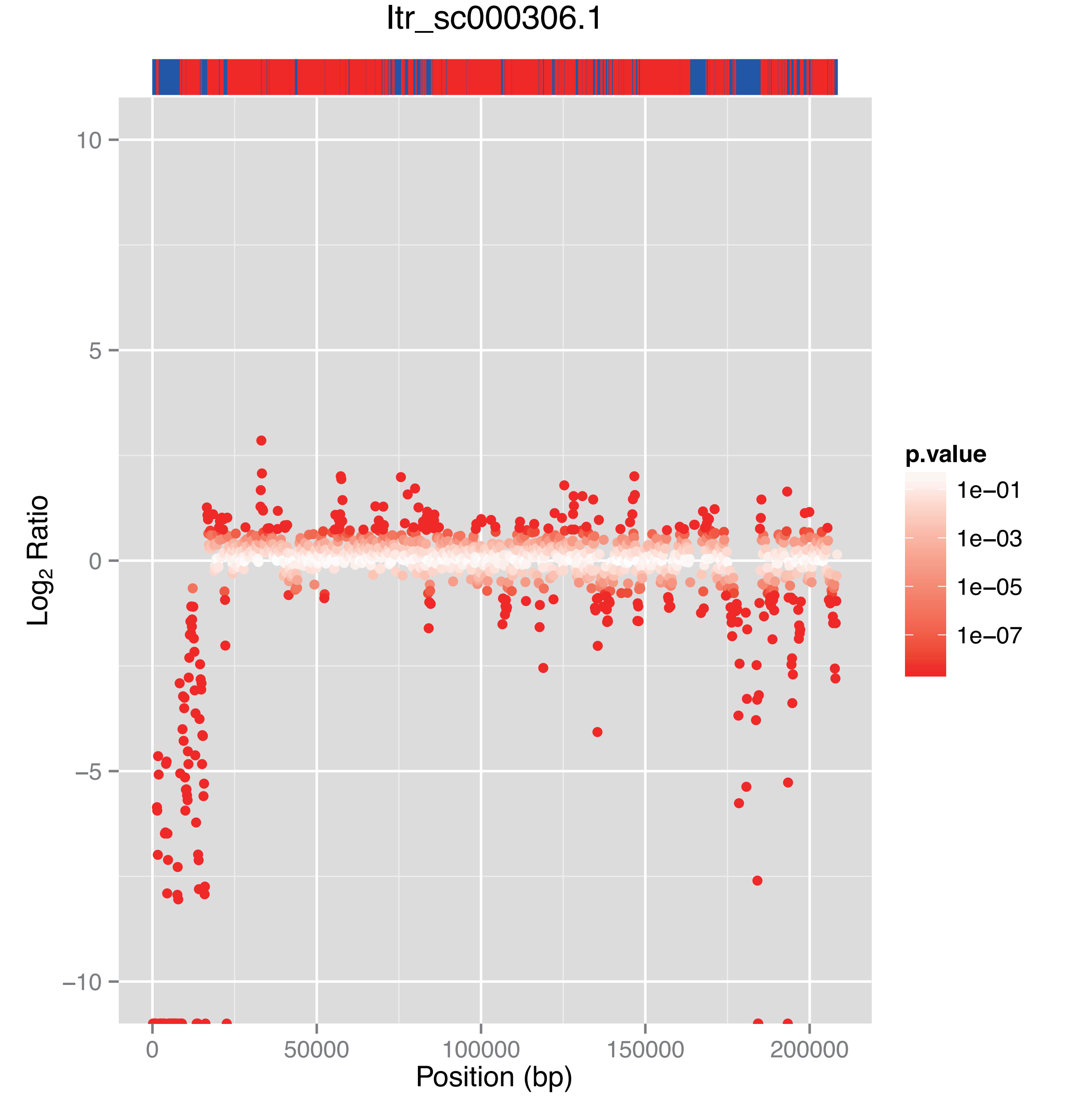
ltr_sc000306.1
10
5
0
−5
−10
Log2 Ratio
0
50000
100000
150000
200000
Position (bp)
p.value
1e−01
1e−03
1e−05
1e−07

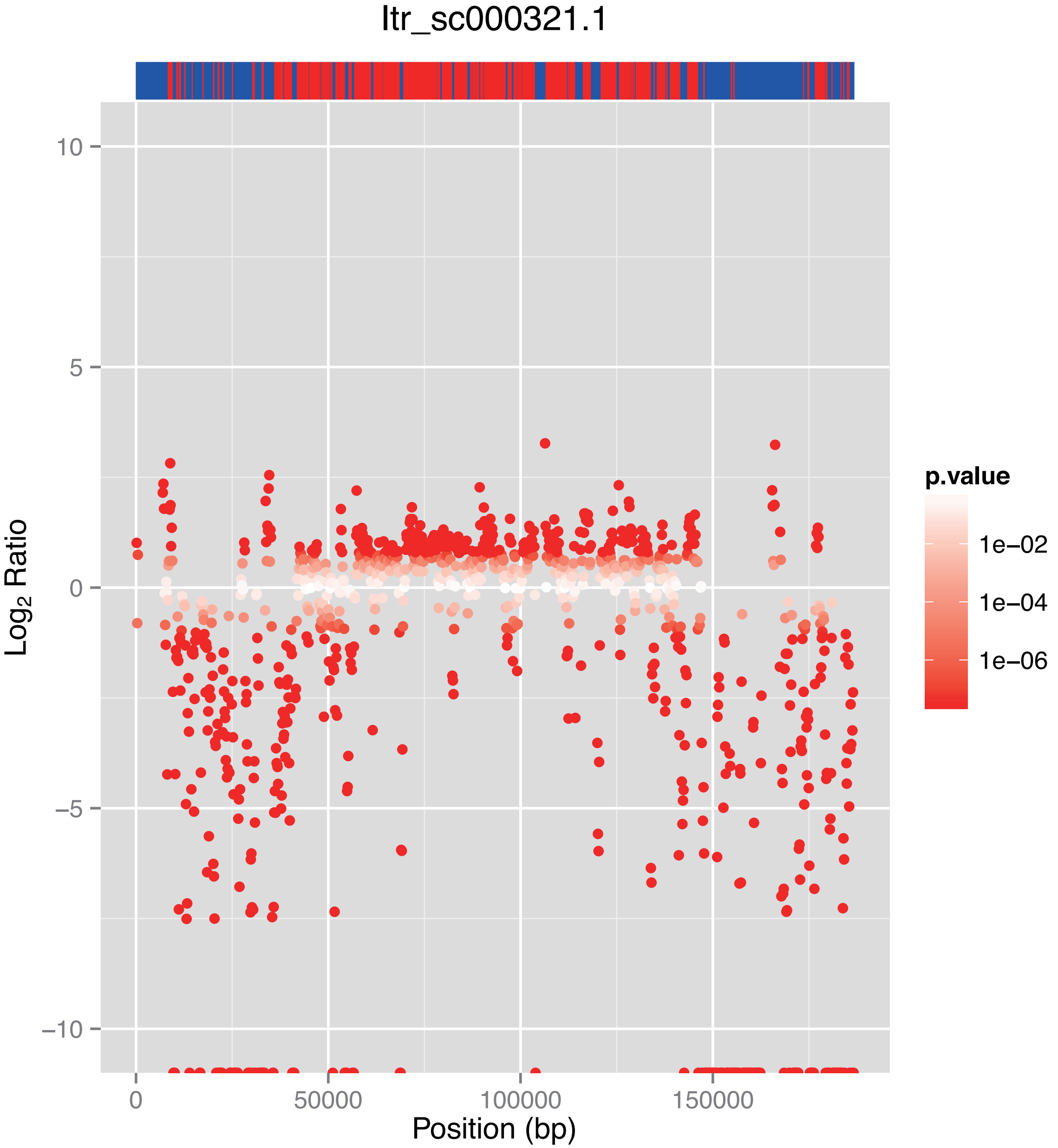
ltr_sc000321.1
Log2 Ratio
10
5
0
−5
−10
0
50000
100000
150000
Position (bp)
p.value
1e−02
1e−04
1e−06

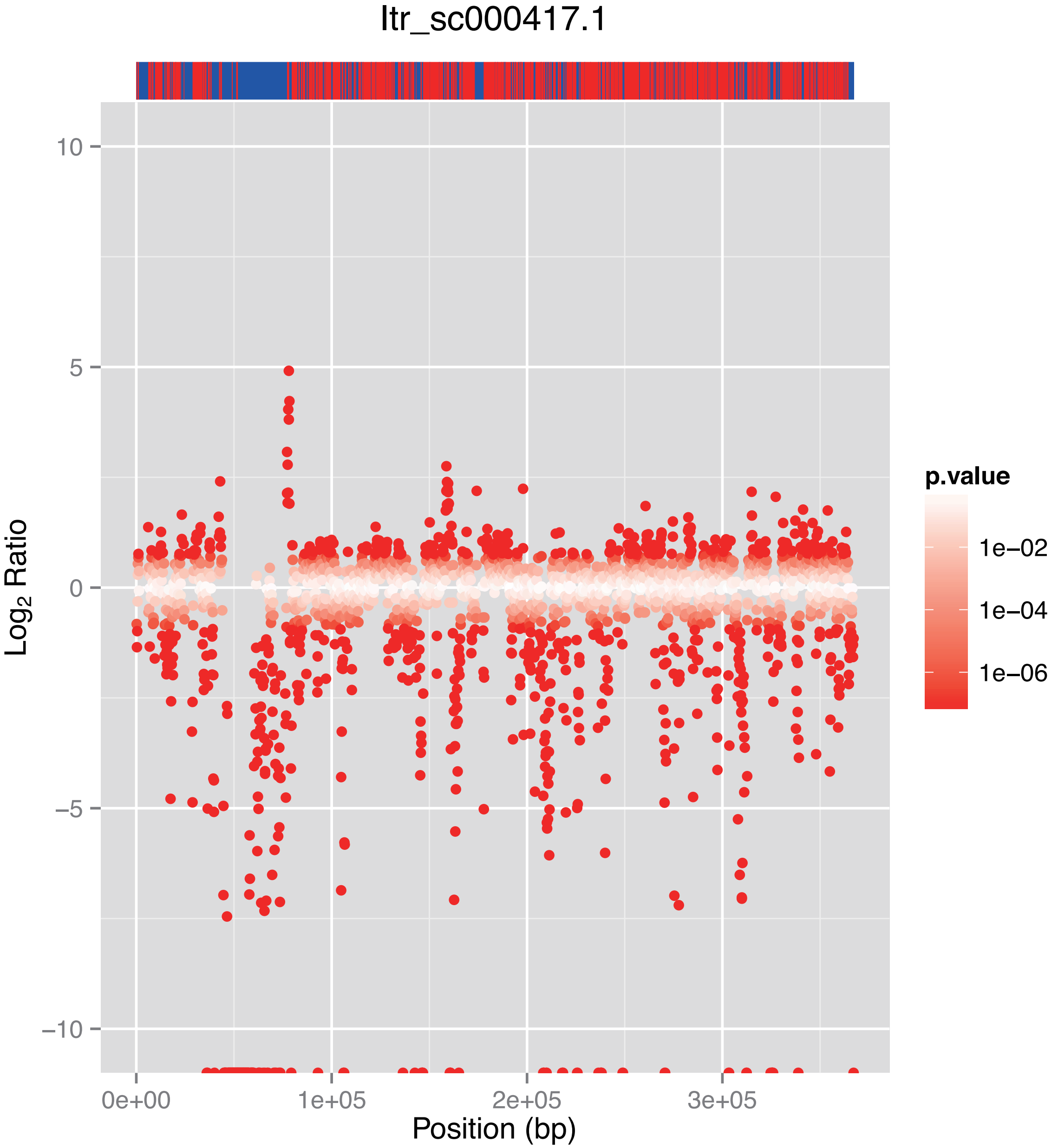

ltr_sc000417.1
10
5
0
−5
−10
Log2 Ratio
0e+00
1e+05
2e+05
3e+05
Position (bp)
p.value
1e−02
1e−04
1e−06

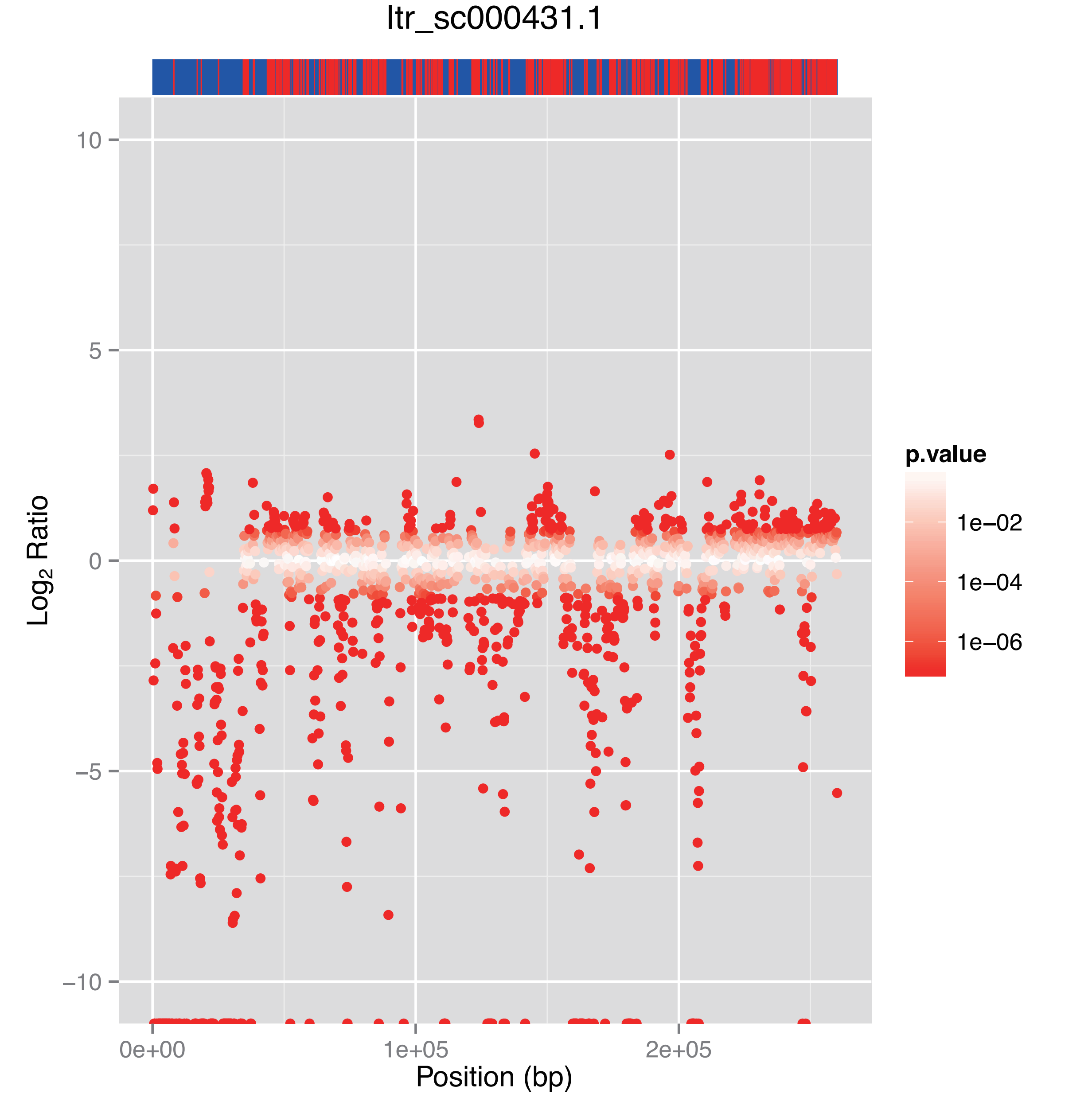
ltr_sc000431.1
10
5
0
−5
−10
Log2 Ratio
0e+00
1e+05
2e+05
Position (bp)
p.value
1e−02
1e−04
1e−06

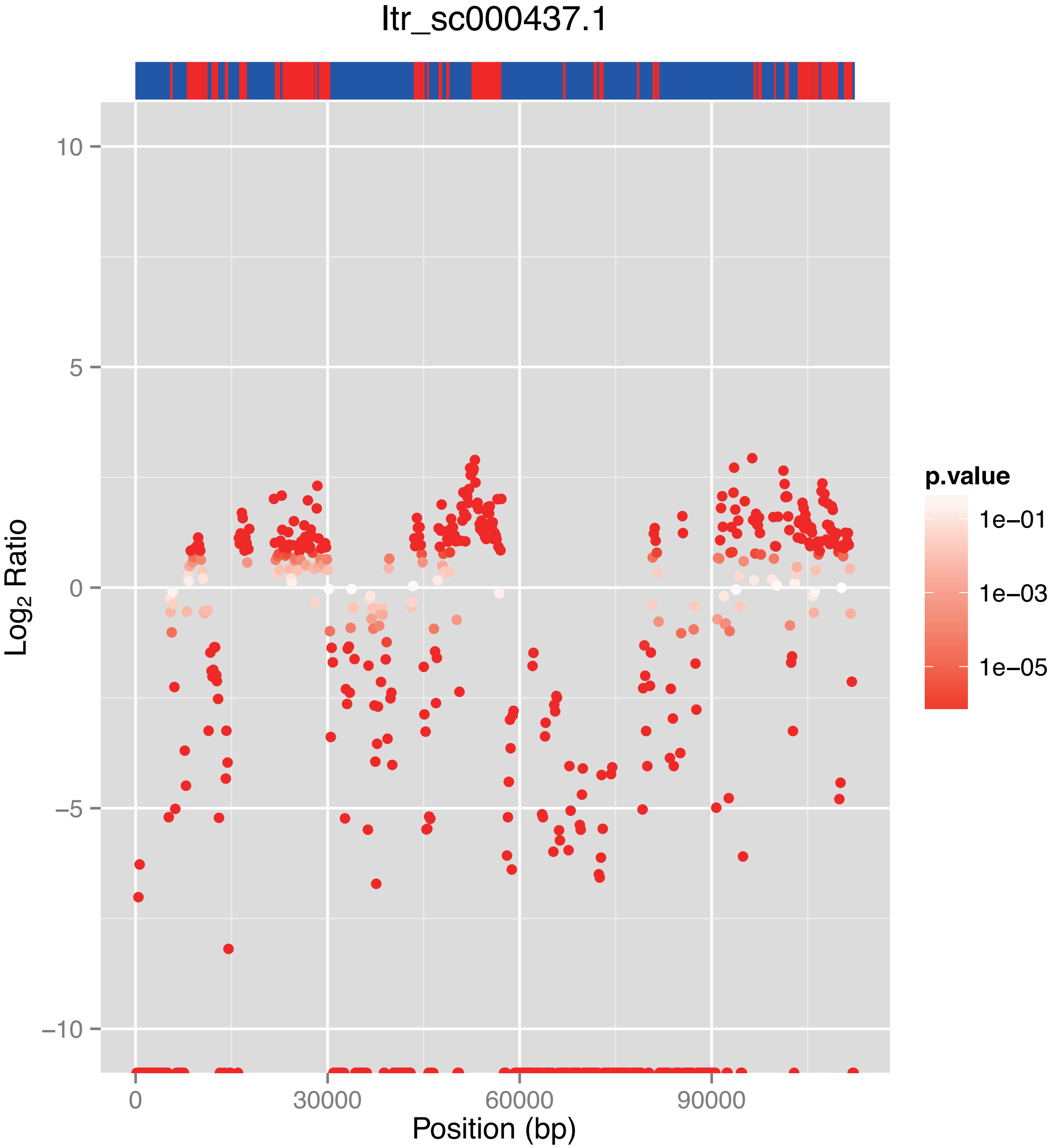

ltr_sc000437.1
10
5
0
−5
−10
Log2 Ratio
0
30000
60000
90000
Position (bp)
p.value
1e−01
1e−03
1e−05

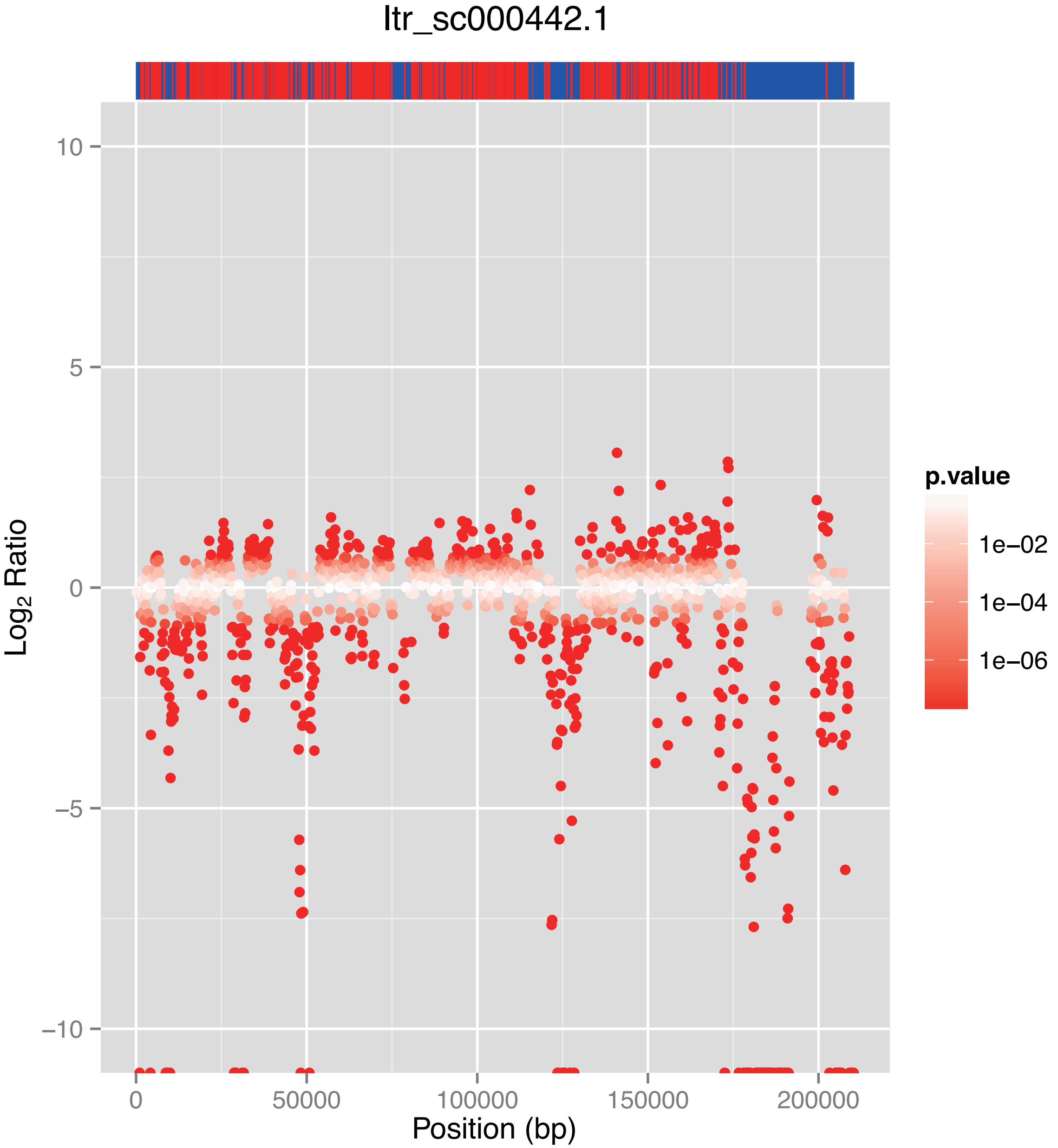

ltr_sc000442.1
p.value
1e−02
1e−04
1e−06
Log2 Ratio
10
5
0
−5
−10
0
50000
100000
150000
200000
Position (bp)

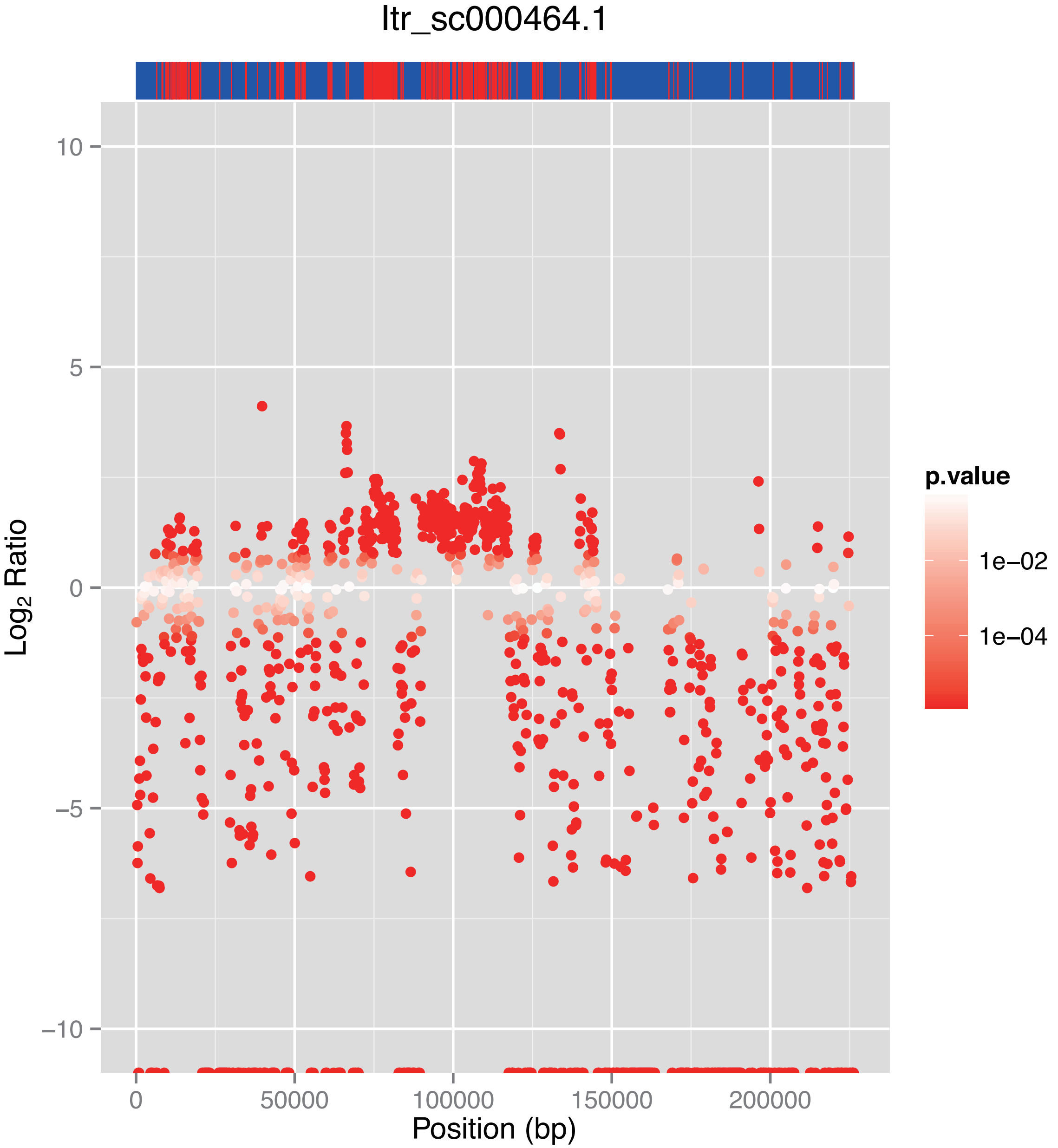

ltr_sc000464.1
10
5
0
−5
−10
Log_2 Ratio
0
50000
100000
150000
200000
Position (bp)
p.value
1e−02
1e−04

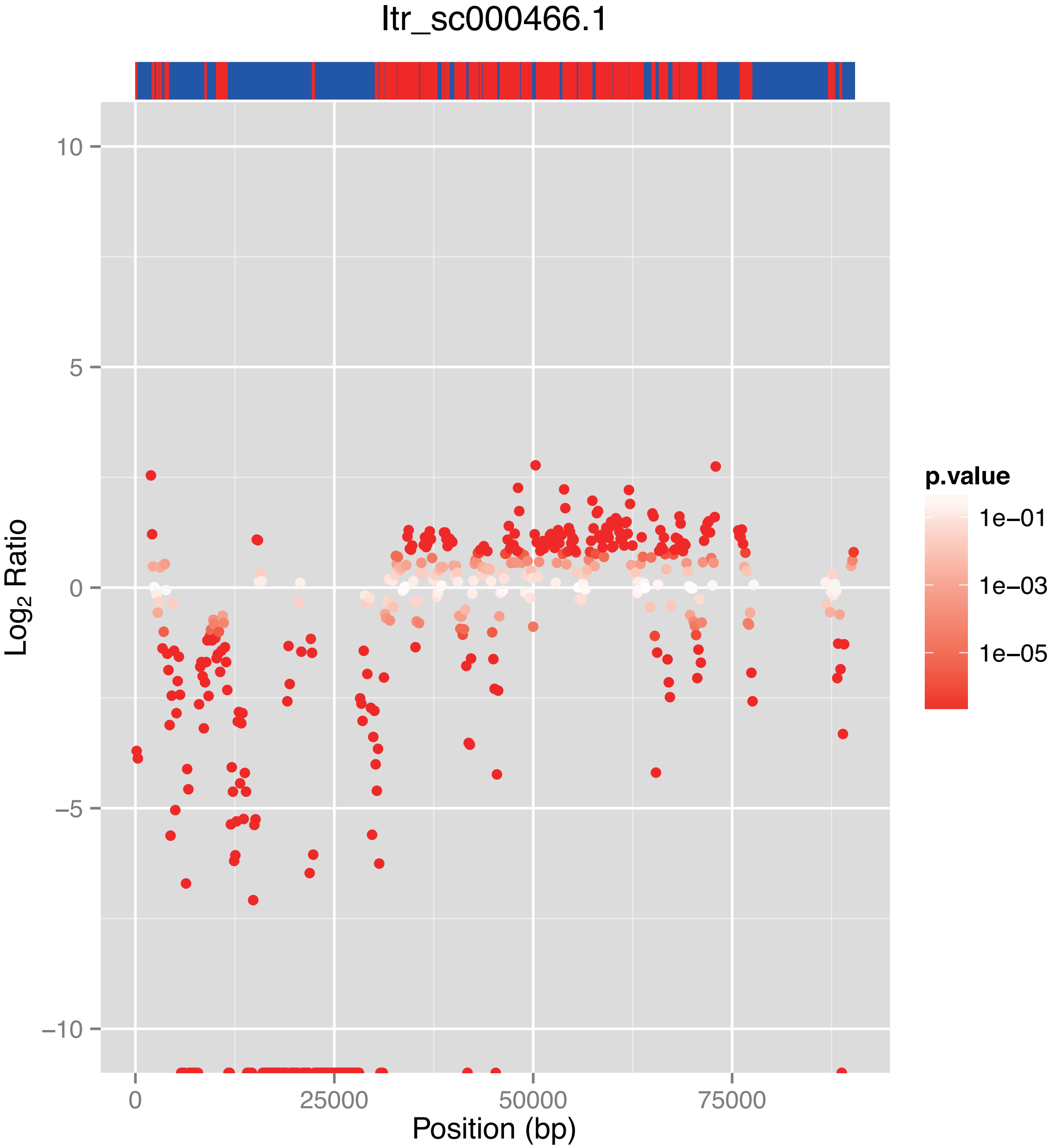
ltr_sc000466.1
Log₂ Ratio
10
5
0
−5
−10
0
25000
50000
75000
Position (bp)
p.value
1e−01
1e−03
1e−05

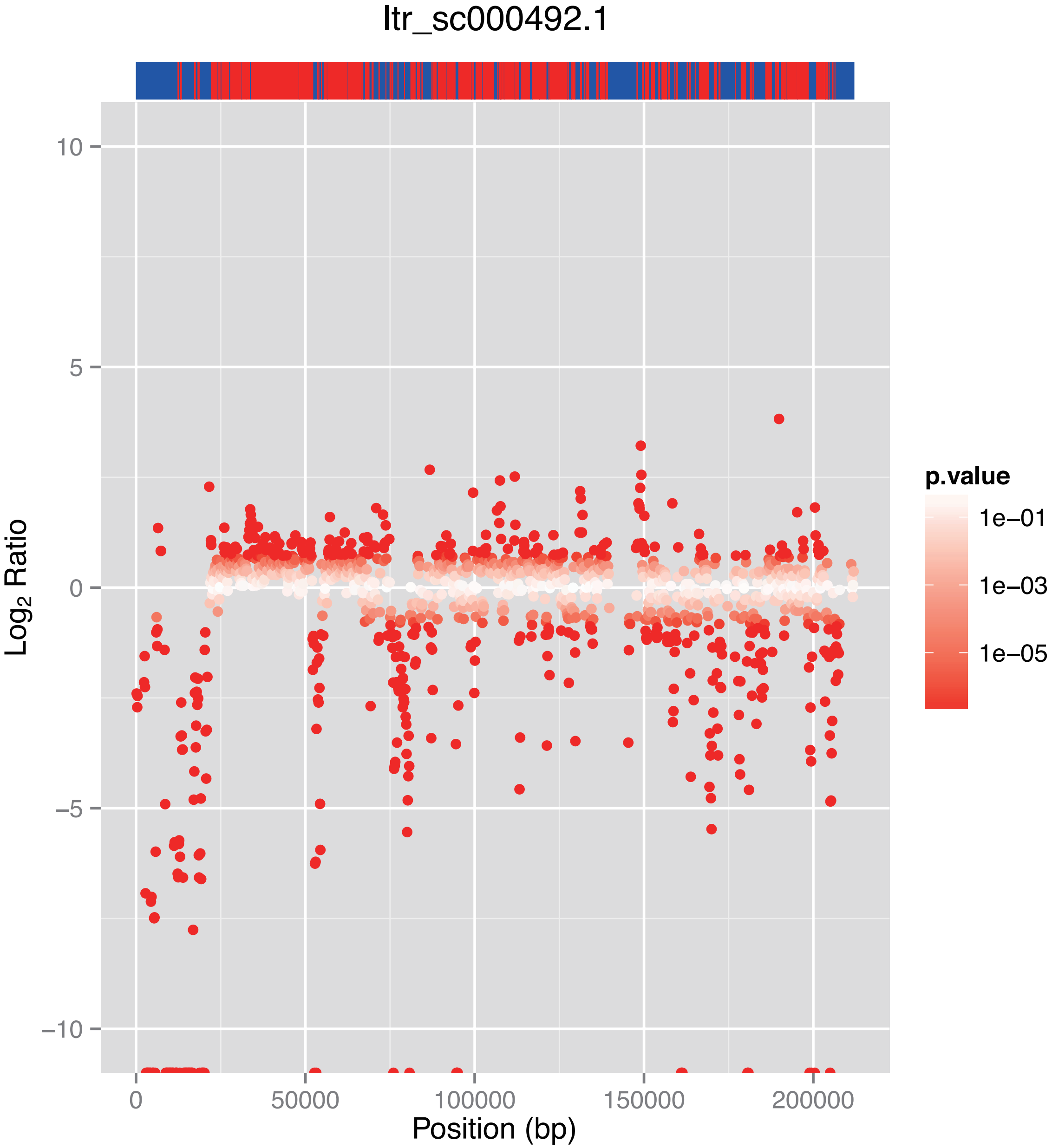

ltr_sc000492.1
10
5
0
−5
−10
Log_2 Ratio
0
50000
100000
150000
200000
Position (bp)
p.value
1e−01
1e−03
1e−05

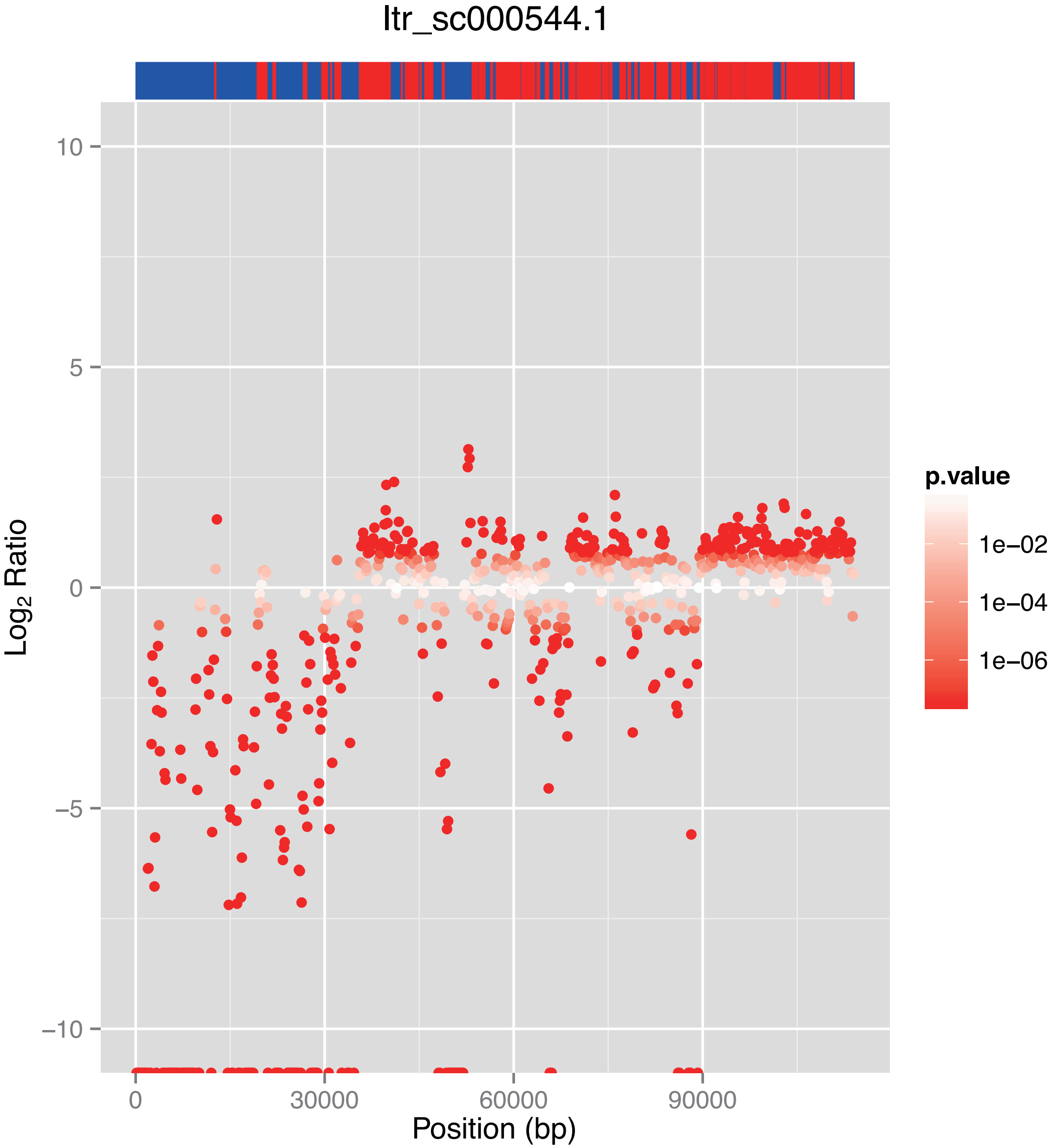

ltr_sc000544.1
10
5
0
−5
−10
Log2 Ratio
0
30000
60000
90000
Position (bp)
p.value
1e−02
1e−04
1e−06

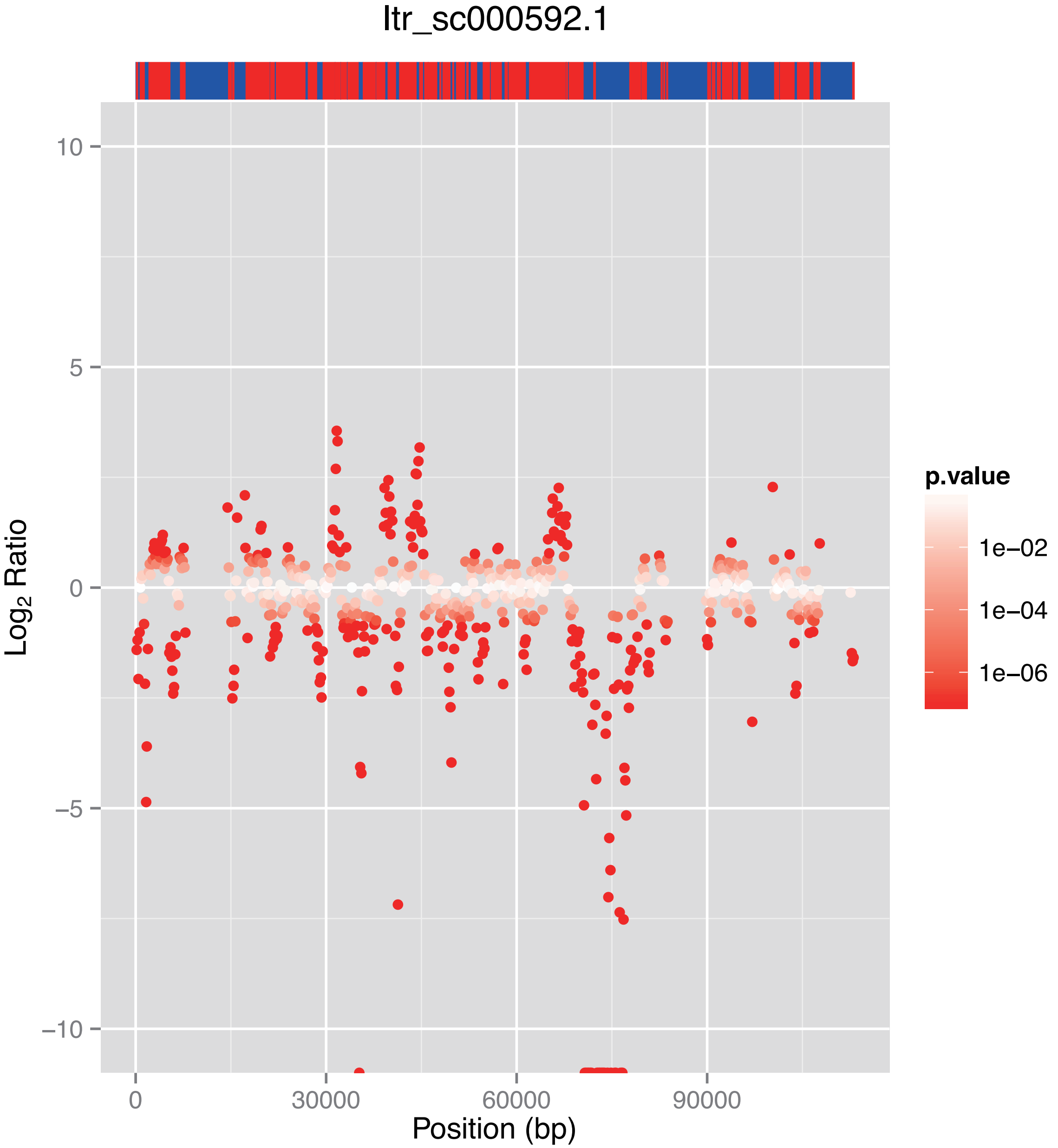

ltr_sc000592.1
10
5
0
−5
−10
Log2 Ratio
0
30000
60000
90000
Position (bp)
p.value
1e−02
1e−04
1e−06

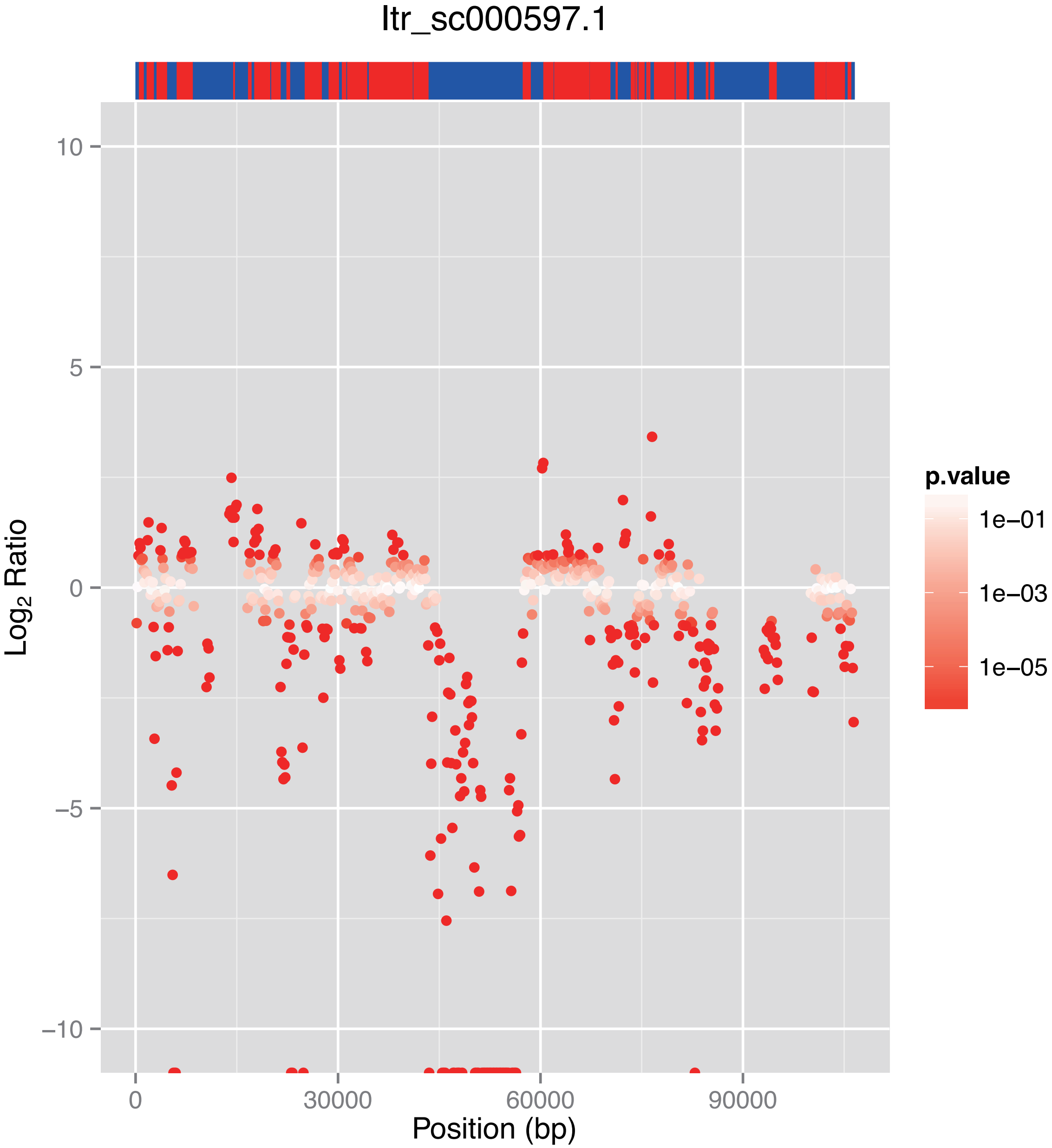

ltr_sc000597.1
p.value
1e−01
1e−03
1e−05
10
5
0
−5
−10
Log2 Ratio
0
30000
60000
90000
Position (bp)

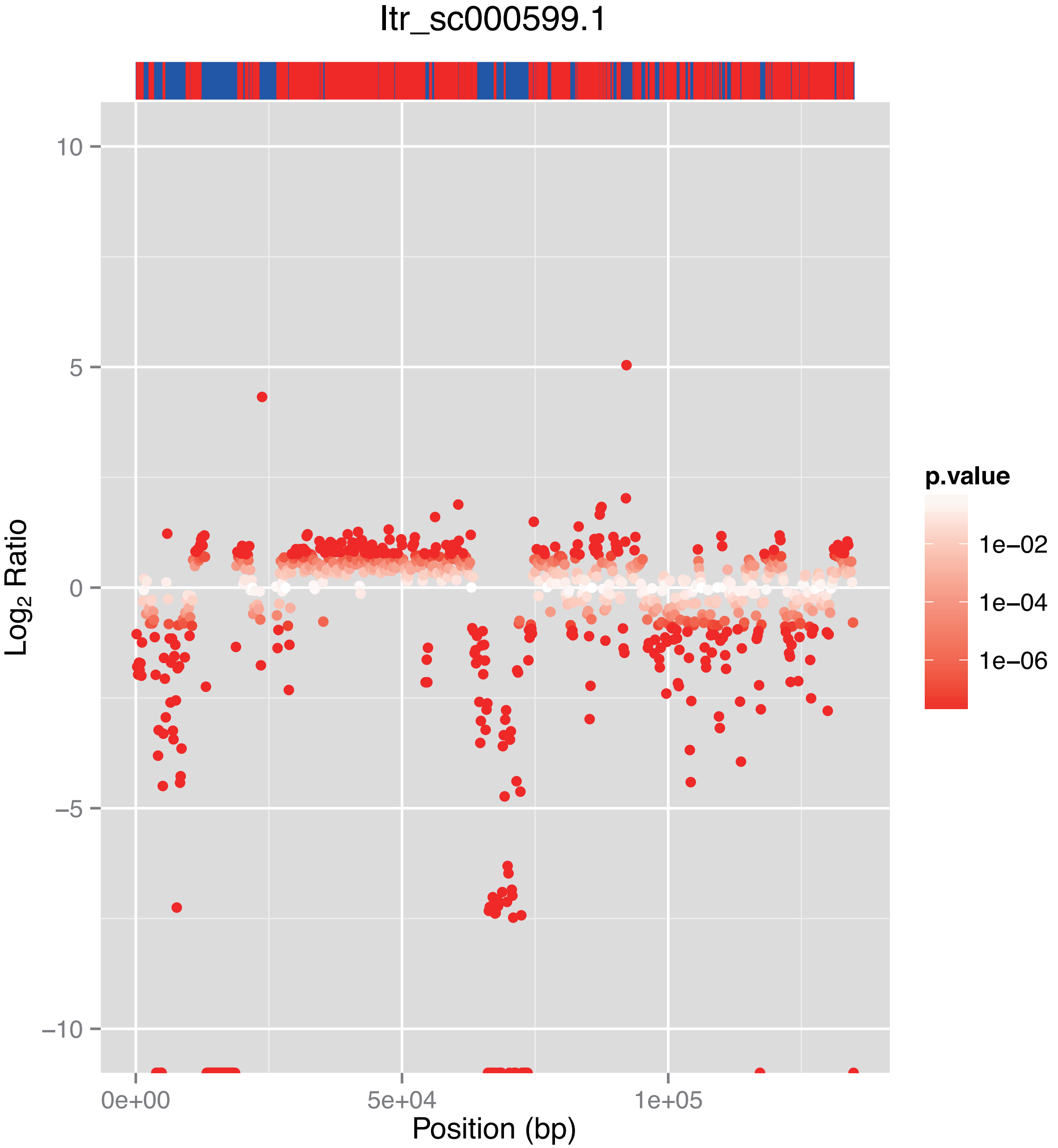

ltr_sc000599.1
10
5
0
−5
−10
Log2 Ratio
0e+00
5e+04
1e+05
Position (bp)
p.value
1e−02
1e−04
1e−06

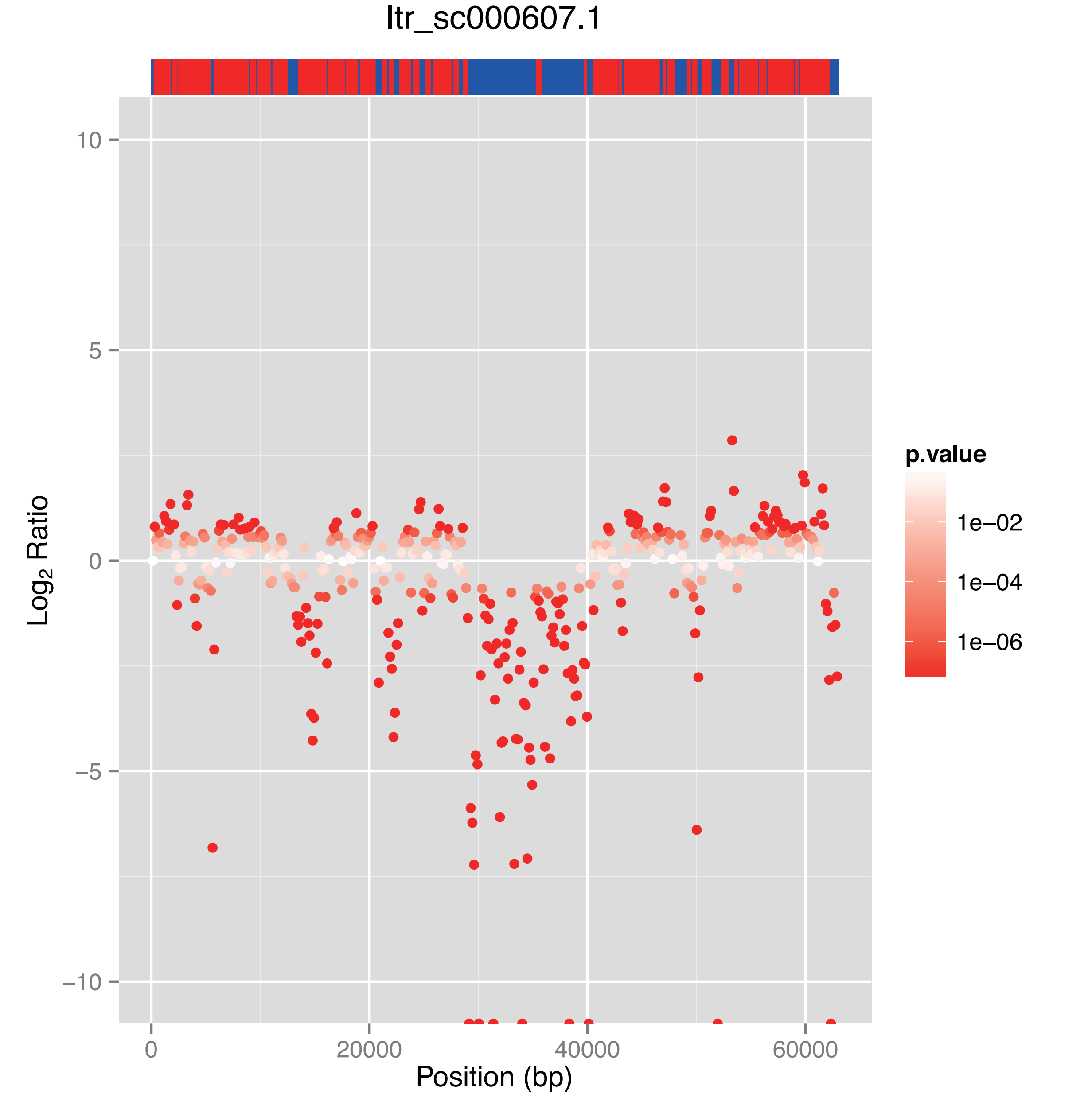

ltr_sc000607.1
10
5
0
−5
−10
Log2 Ratio
0
20000
40000
60000
Position (bp)
p.value
1e−02
1e−04
1e−06

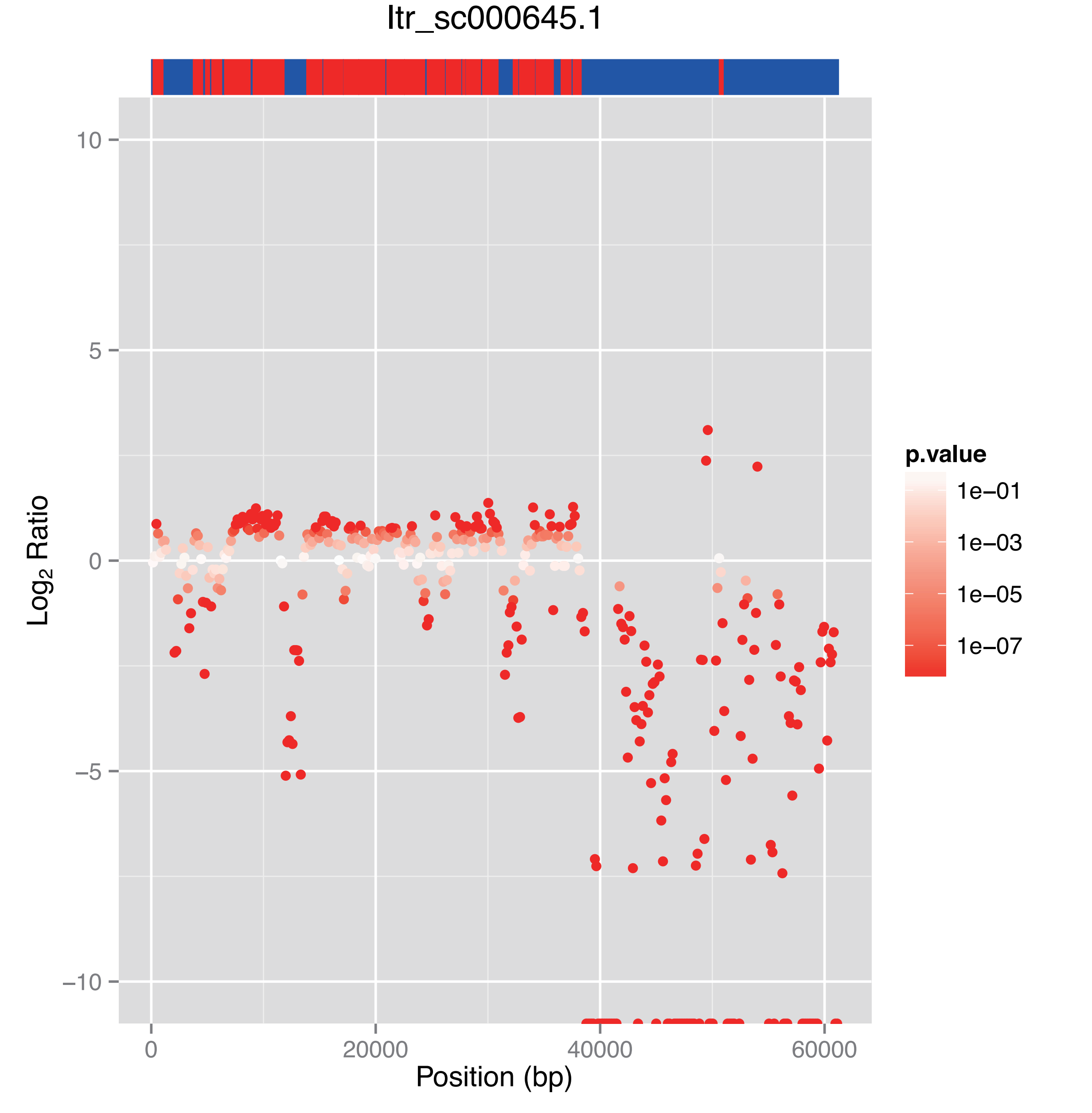
ltr_sc000645.1
10
5
0
−5
−10
Log2 Ratio
0
20000
40000
60000
Position (bp)
p.value
1e−01
1e−03
1e−05
1e−07

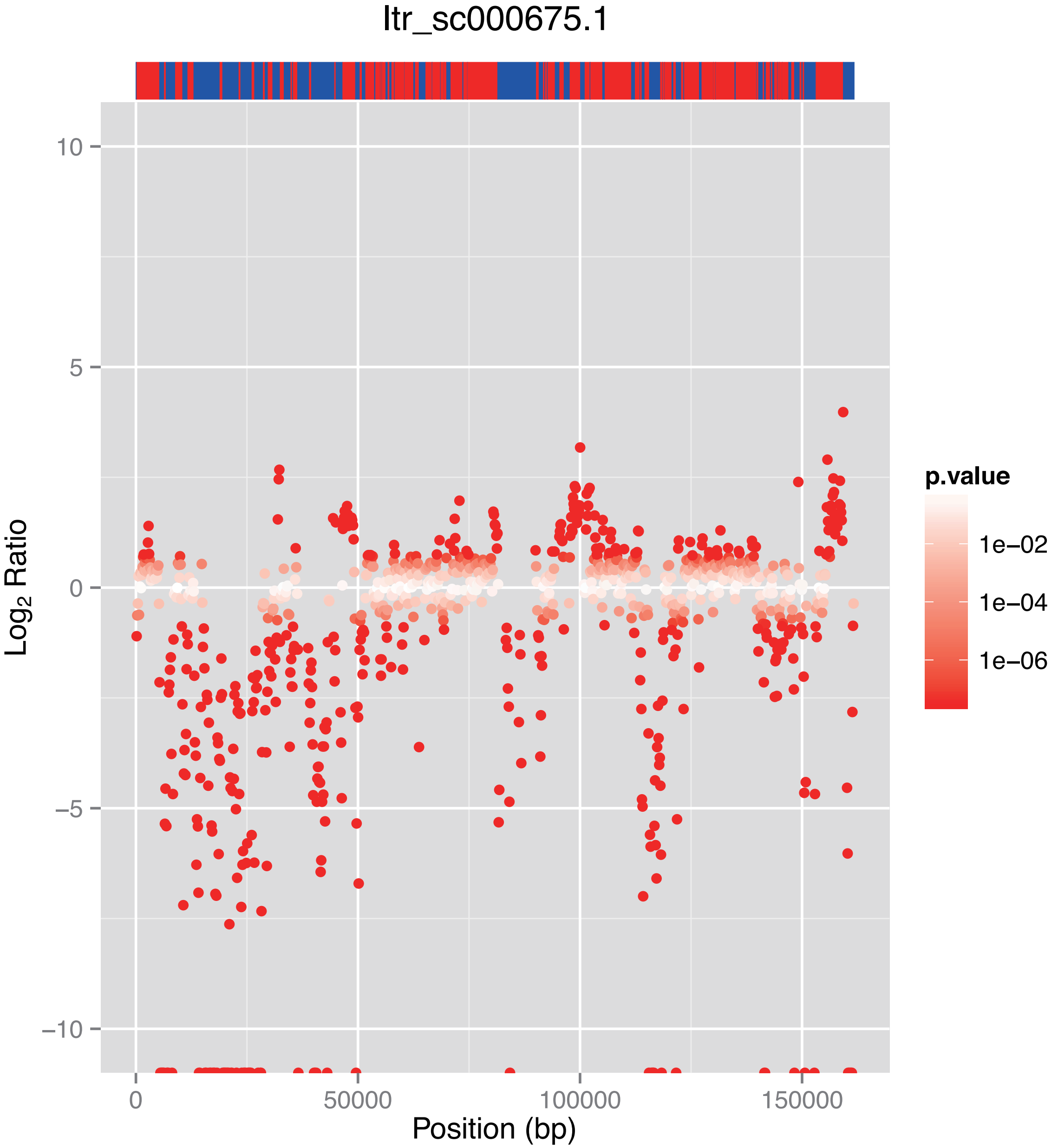
ltr_sc000675.1
Log2 Ratio
Position (bp)
p.value
1e−02
1e−04
1e−06
10
5
0
−5
−10
0
50000
100000
150000

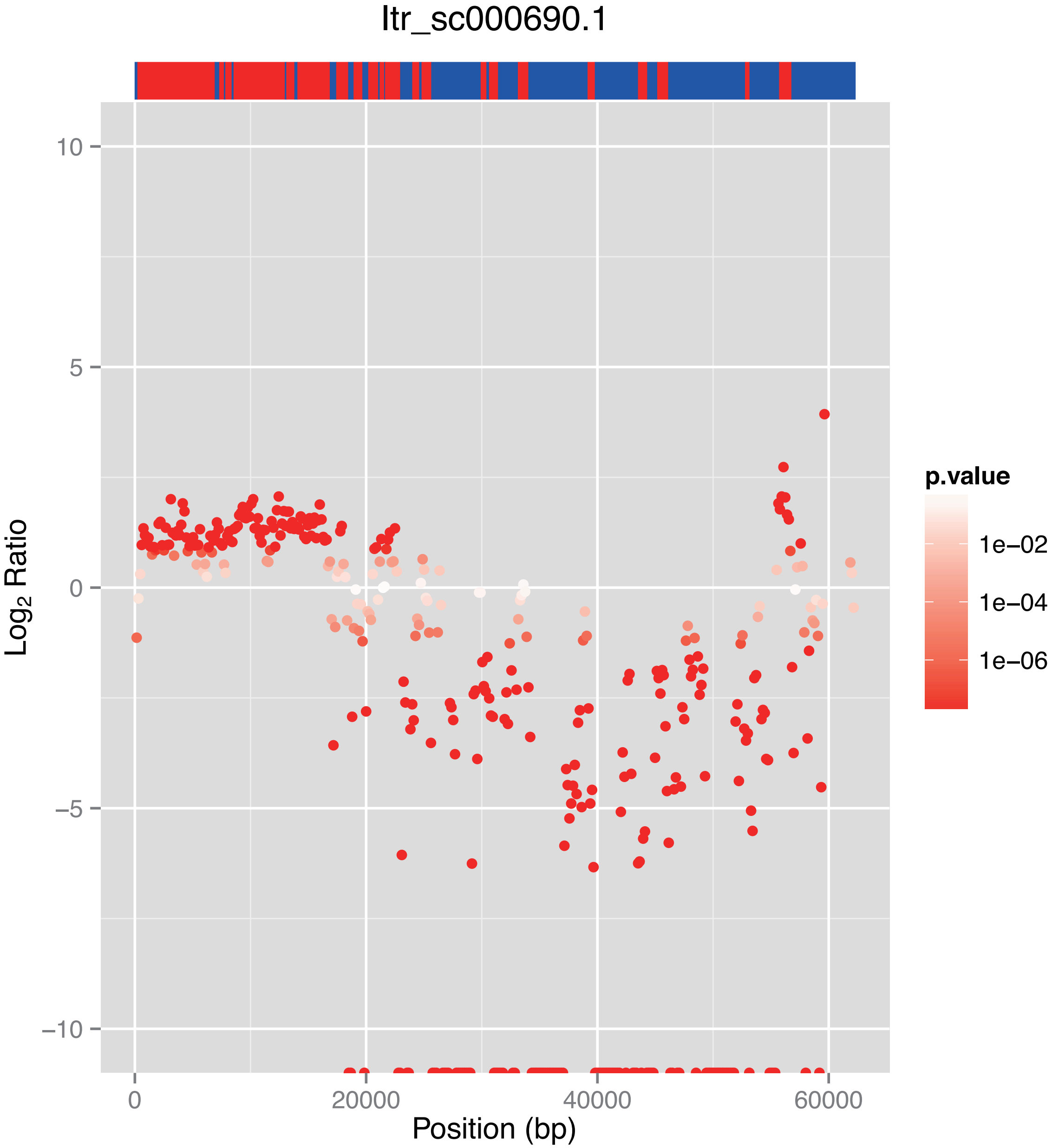

ltr_sc000690.1
10
5
0
−5
−10
Log_2 Ratio
0
20000
40000
60000
Position (bp)
p.value
1e−02
1e−04
1e−06

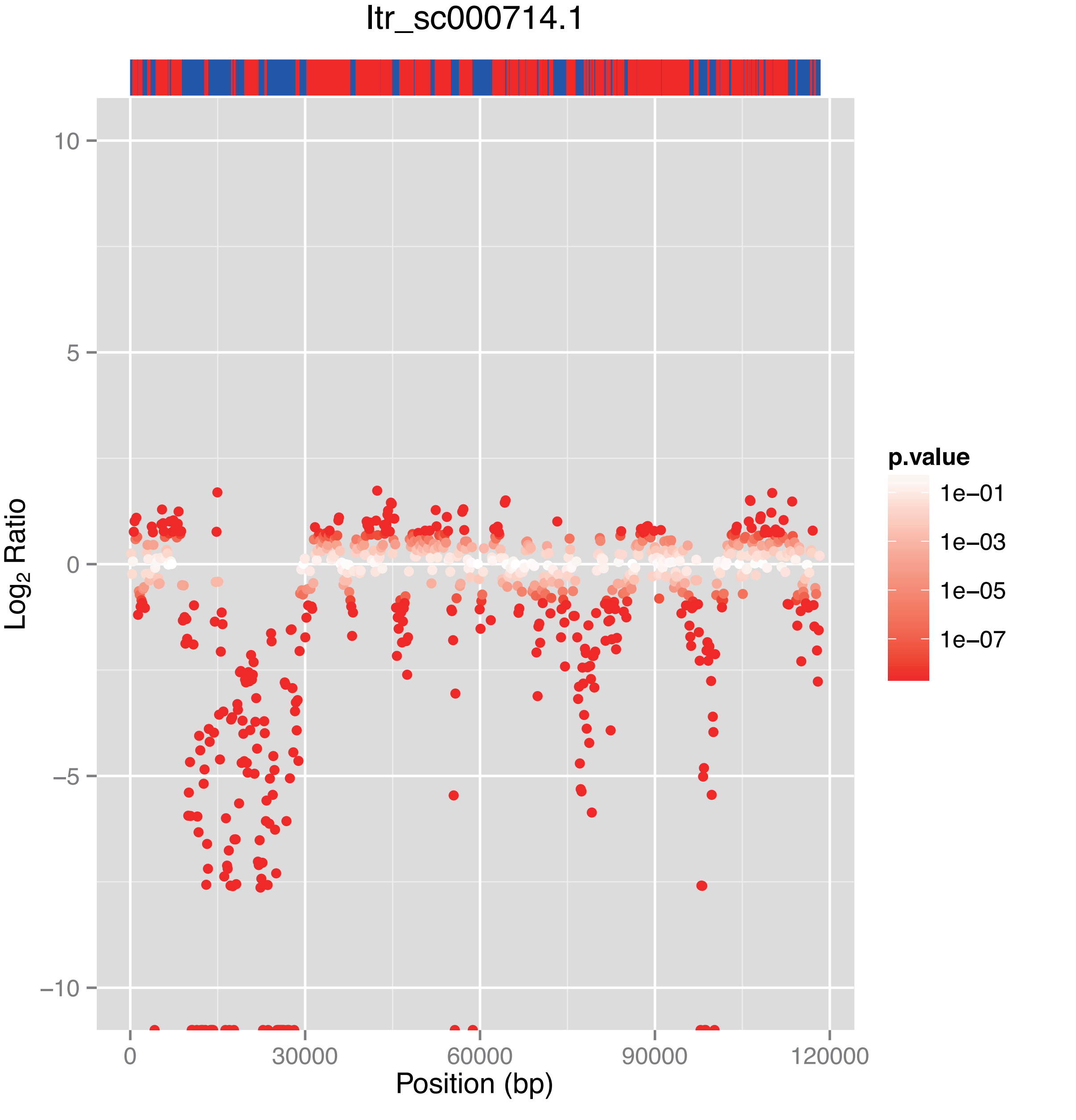
ltr_sc000714.1
Log2 Ratio
Position (bp)
10
5
0
−5
−10
0
30000
60000
90000
120000
p.value
1e−01
1e−03
1e−05
1e−07

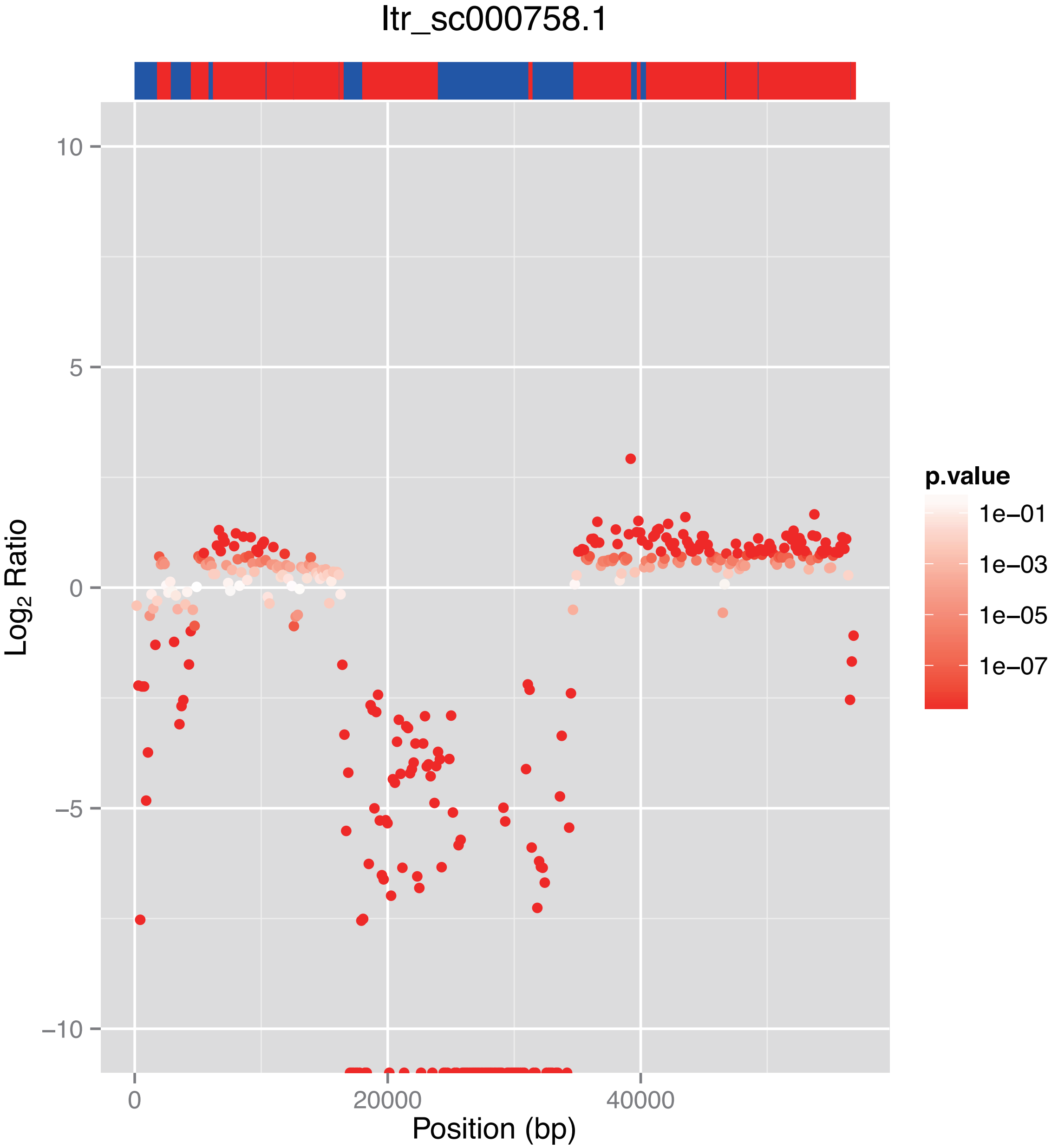
ltr_sc000758.1
10
5
0
−5
−10
Log2 Ratio
0
20000
40000
Position (bp)
p.value
1e−01
1e−03
1e−05
1e−07

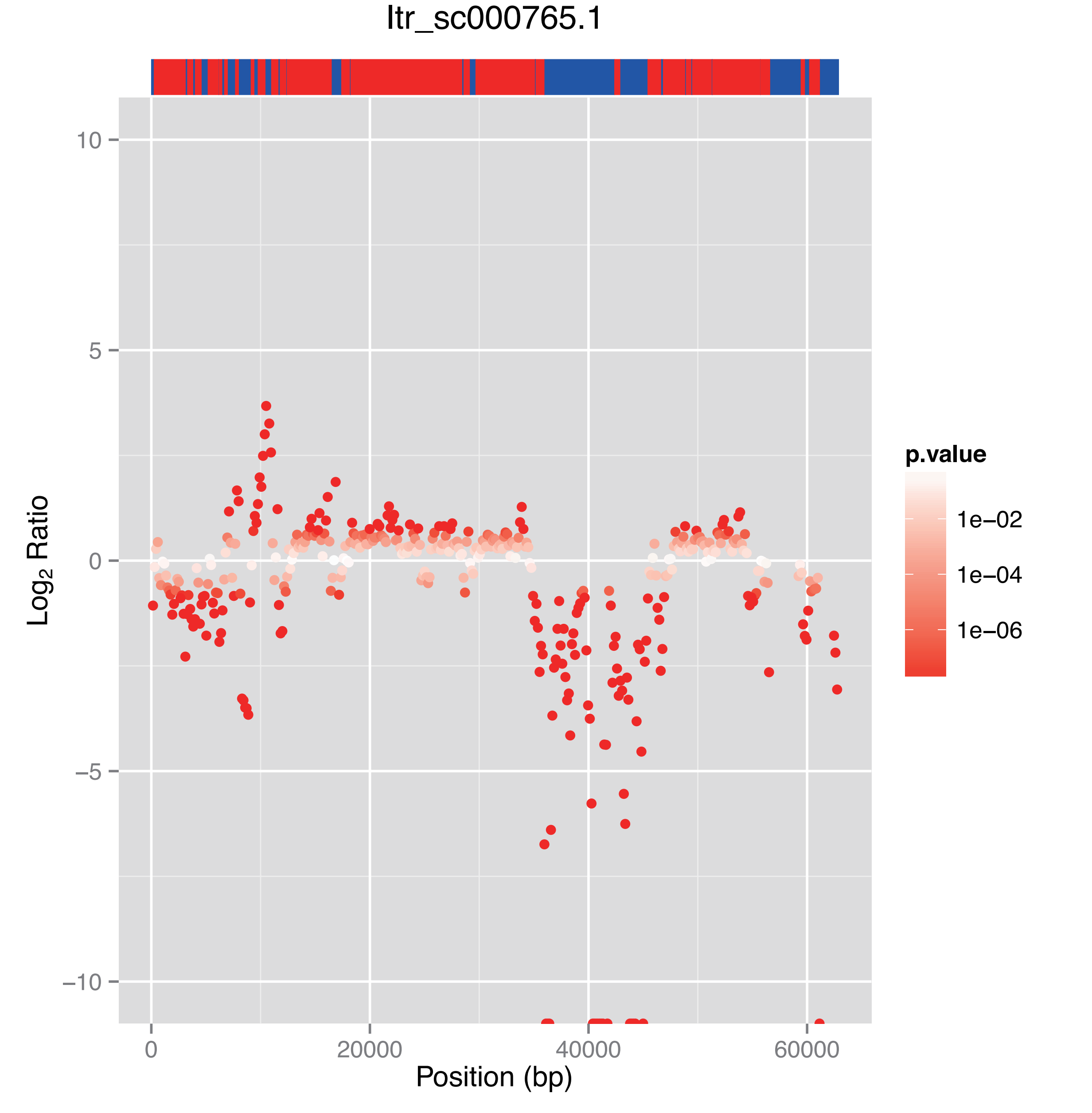

ltr_sc000765.1
10
5
0
−5
−10
Log2 Ratio
0
20000
40000
60000
Position (bp)
p.value
1e−02
1e−04
1e−06

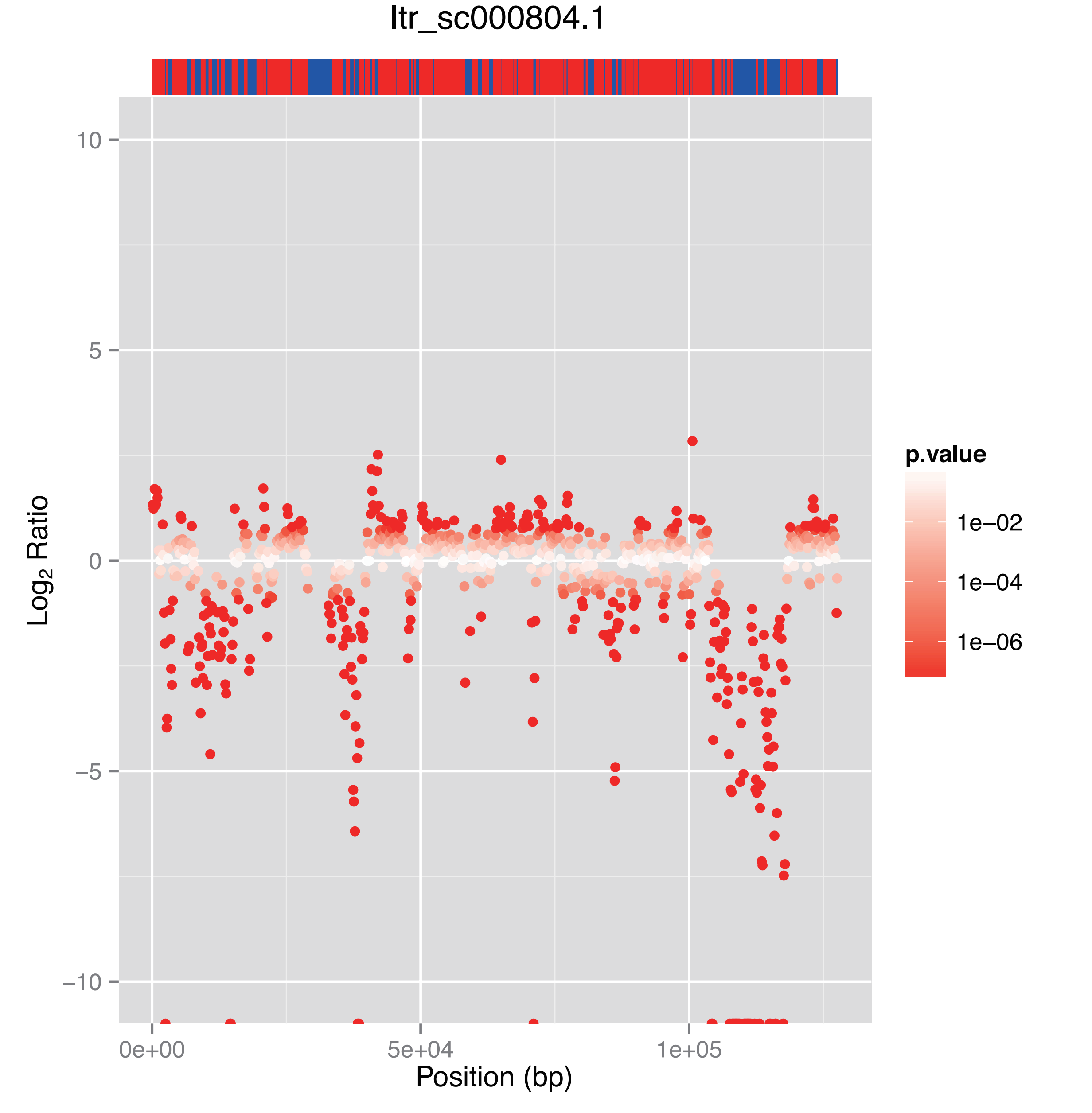
ltr_sc000804.1
10
5
0
−5
−10
Log₂ Ratio
0e+00
5e+04
1e+05
Position (bp)
p.value
1e−02
1e−04
1e−06

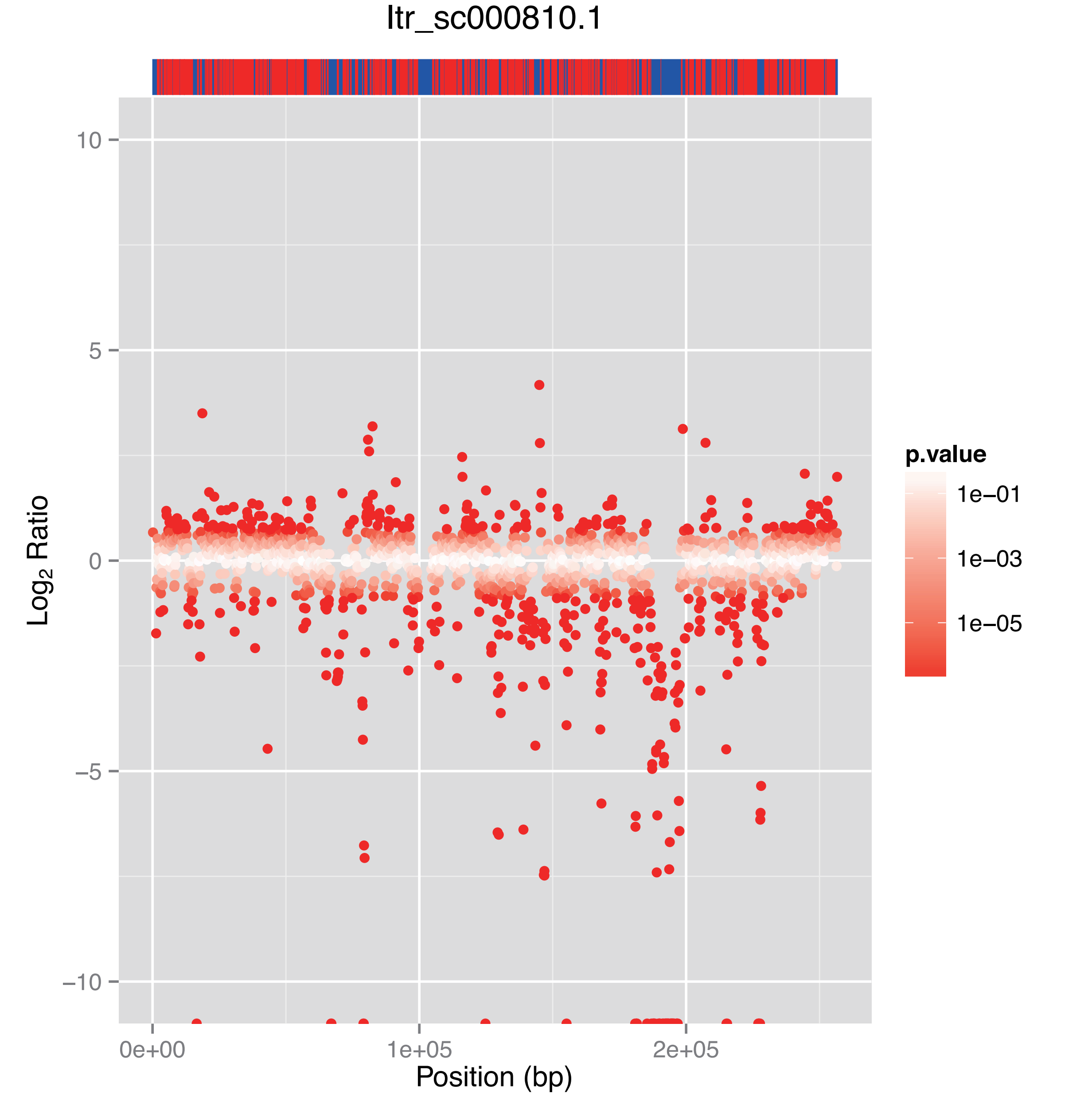

ltr_sc000810.1
10
5
0
−5
−10
Log2 Ratio
0e+00
1e+05
2e+05
Position (bp)
p.value
1e−01
1e−03
1e−05

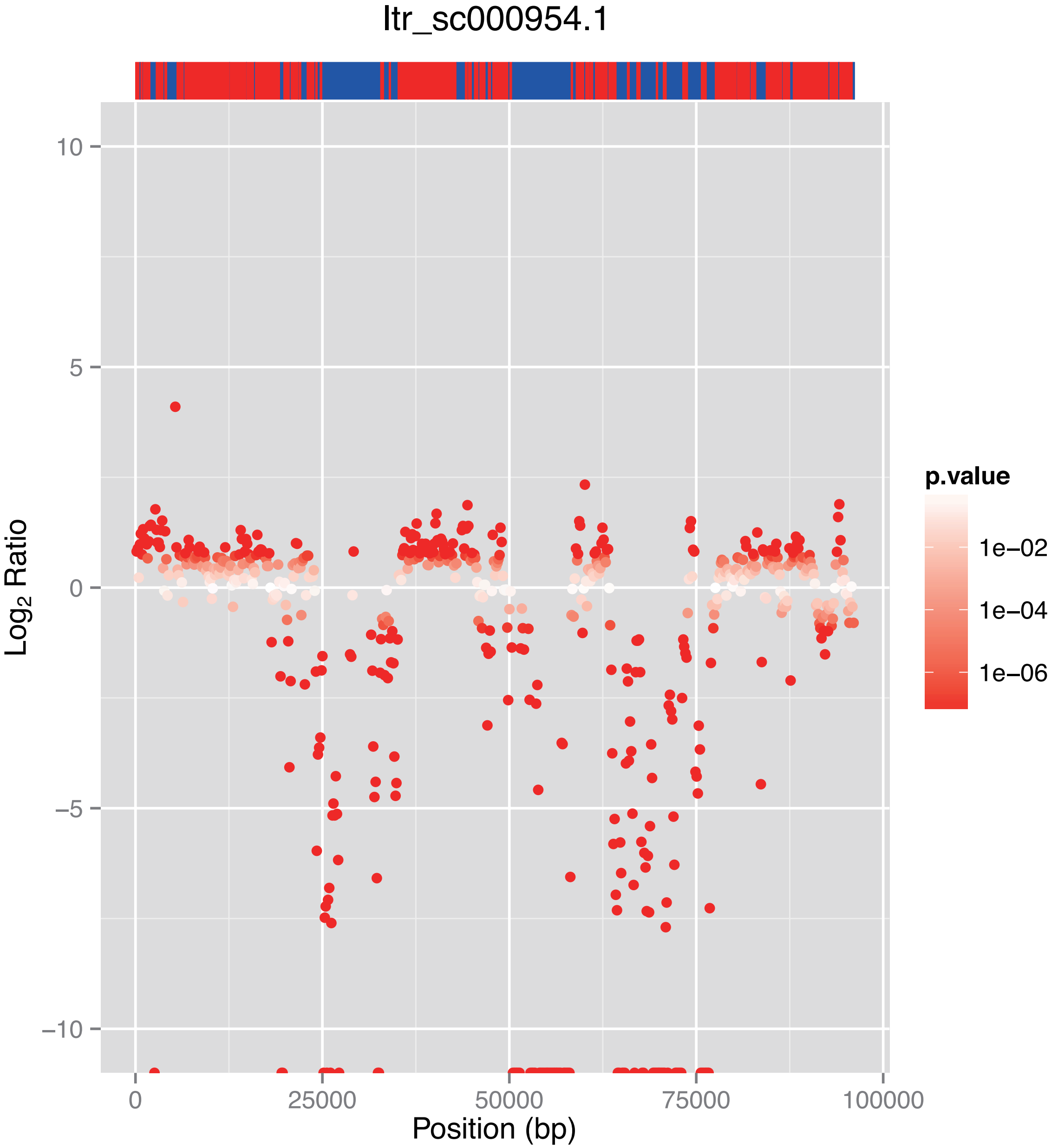
ltr_sc000954.1
10
5
0
−5
−10
Log$_2$ Ratio
0
25000
50000
75000
100000
Position (bp)
p.value
1e−02
1e−04
1e−06

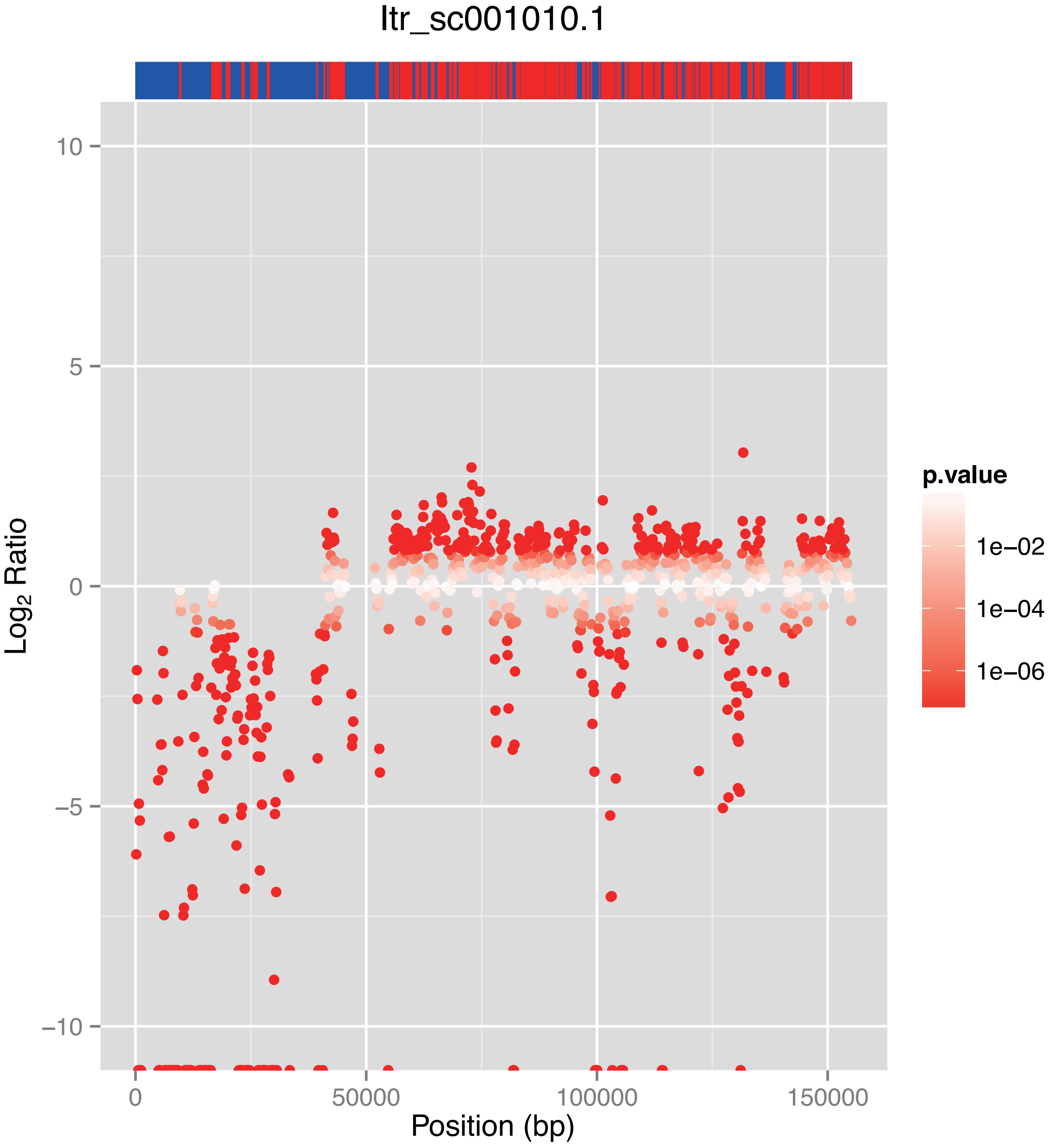

ltr_sc001010.1
Log$_2$ Ratio
10
5
0
−5
−10
0
50000
100000
150000
Position (bp)
p.value
1e−02
1e−04
1e−06

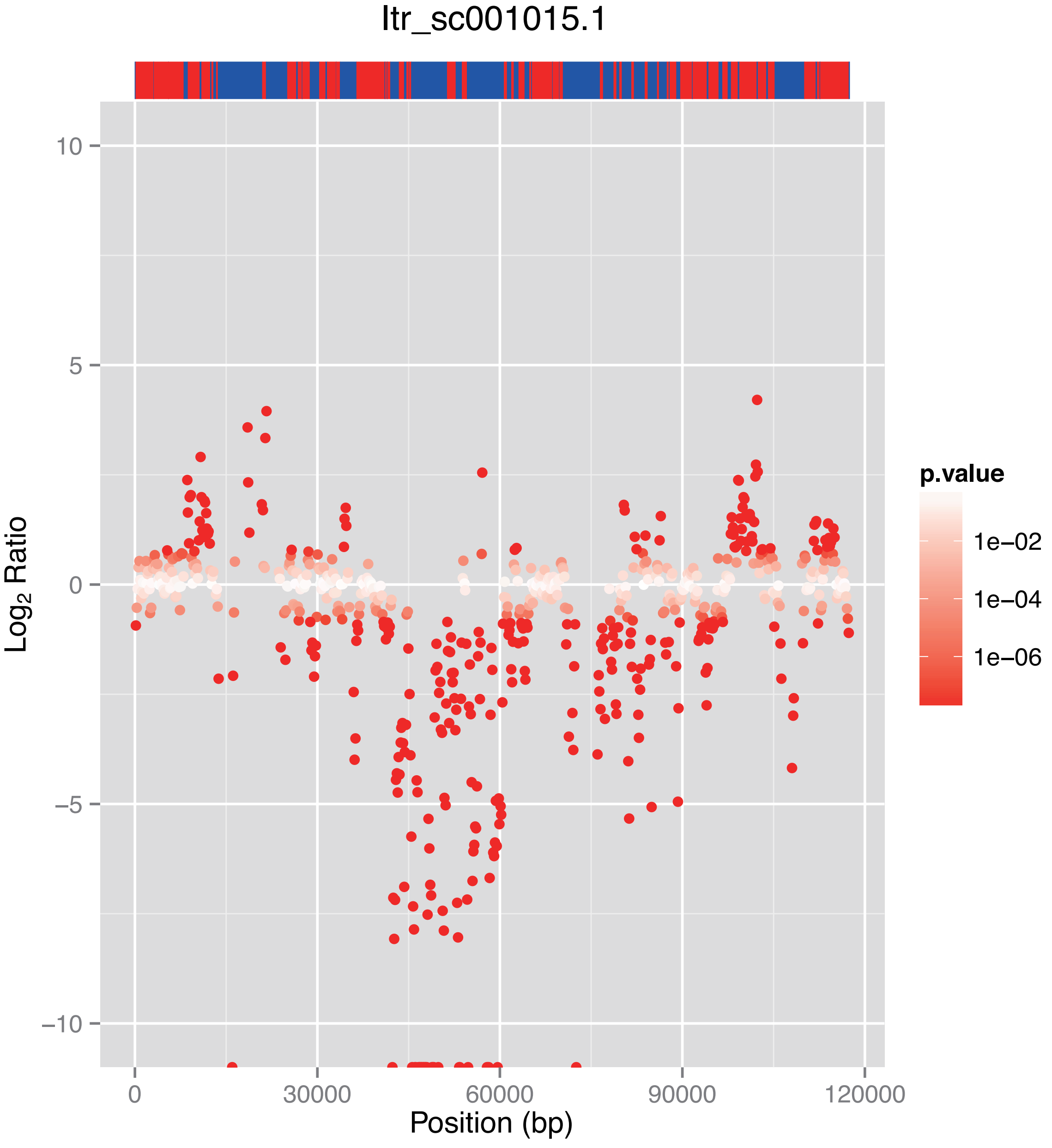

ltr_sc001015.1
10
5
0
−5
−10
Log2 Ratio
0
30000
60000
90000
120000
Position (bp)
p.value
1e−02
1e−04
1e−06

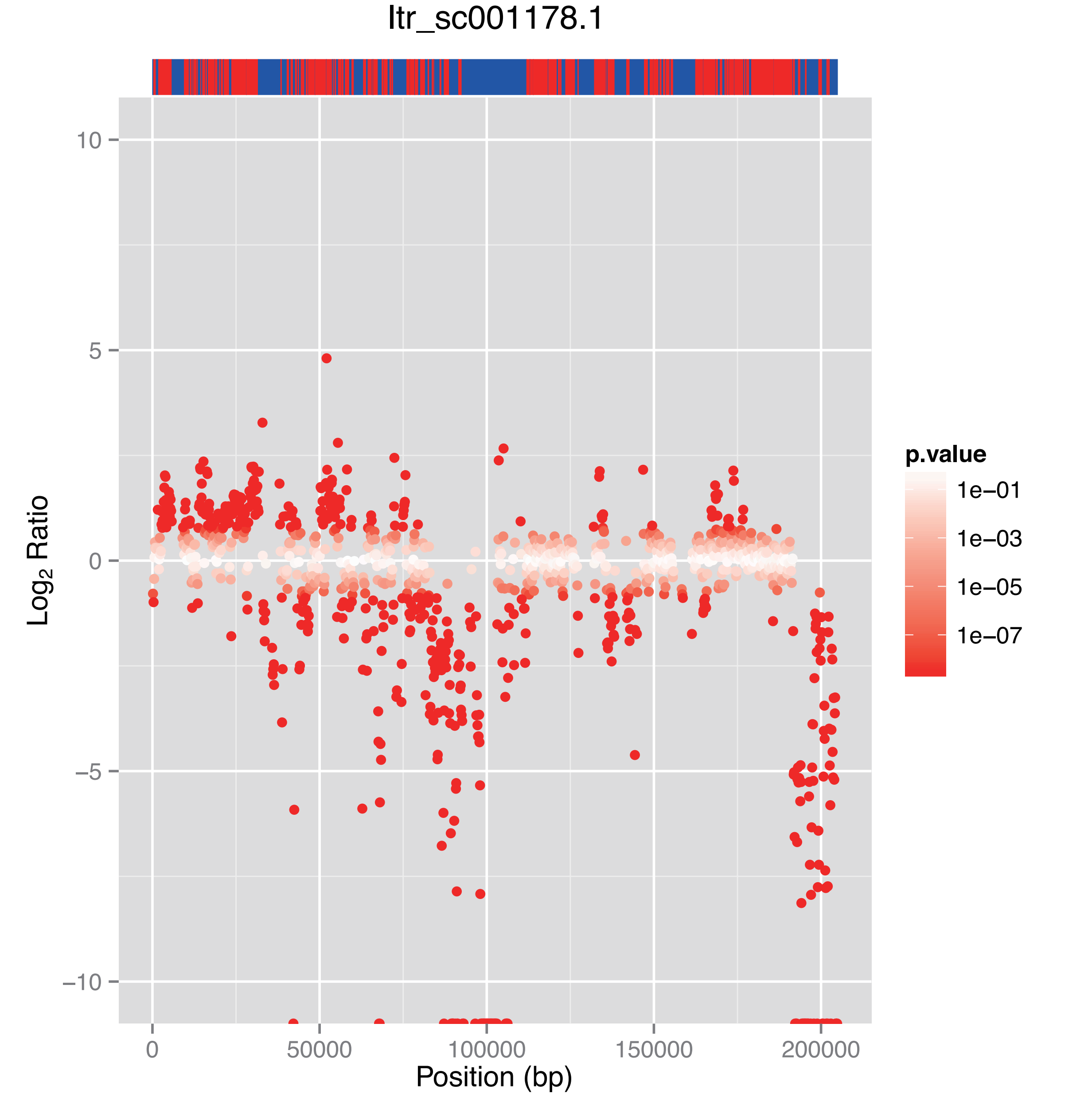

ltr_sc001178.1
10
5
0
−5
−10
Log_2 Ratio
0
50000
100000
150000
200000
Position (bp)
p.value
1e−01
1e−03
1e−05
1e−07

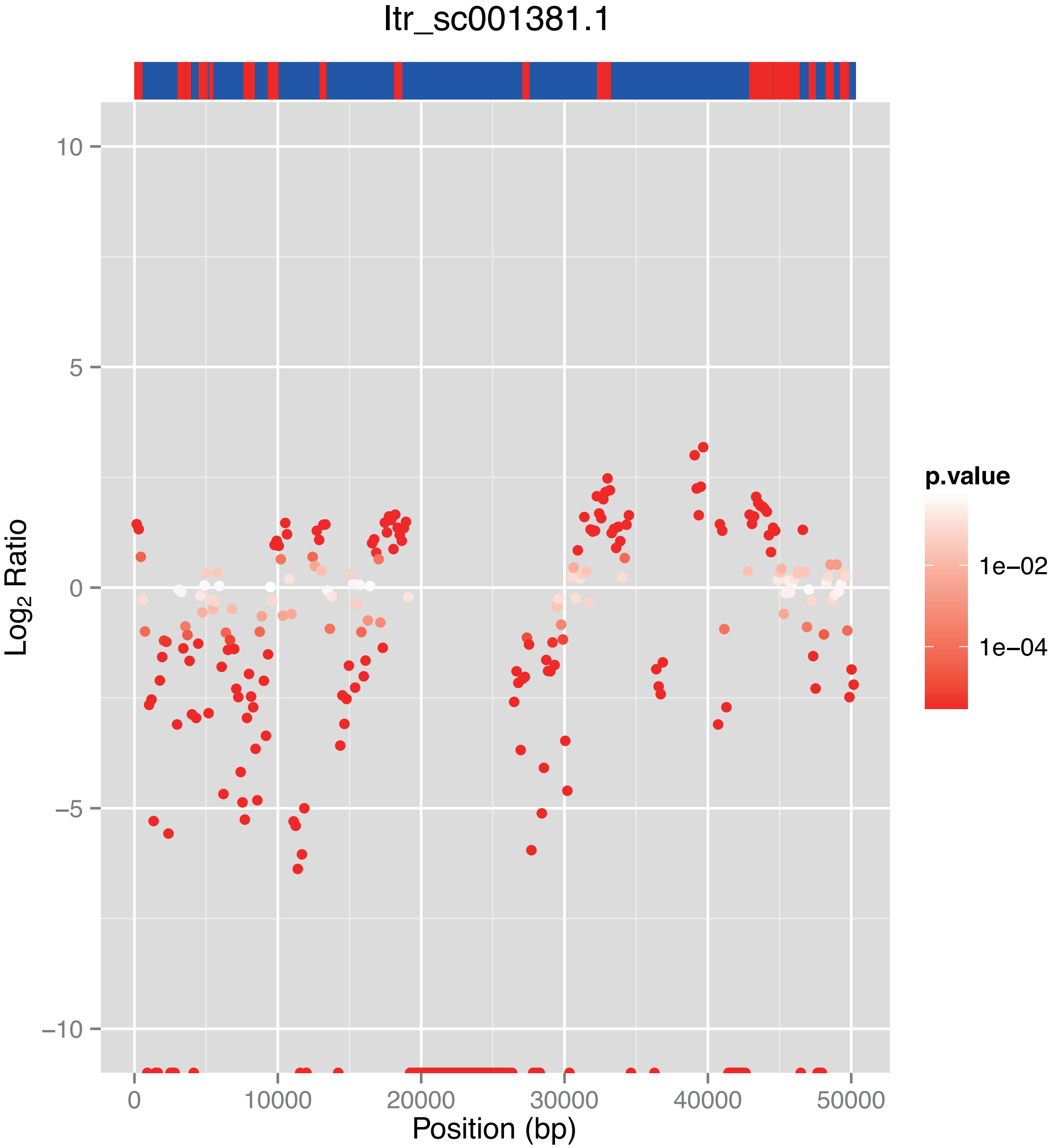
ltr_sc001381.1
Log2 Ratio
Position (bp)
p.value
1e−02
1e−04
10
5
0
−5
−10
0
10000
20000
30000
40000
50000

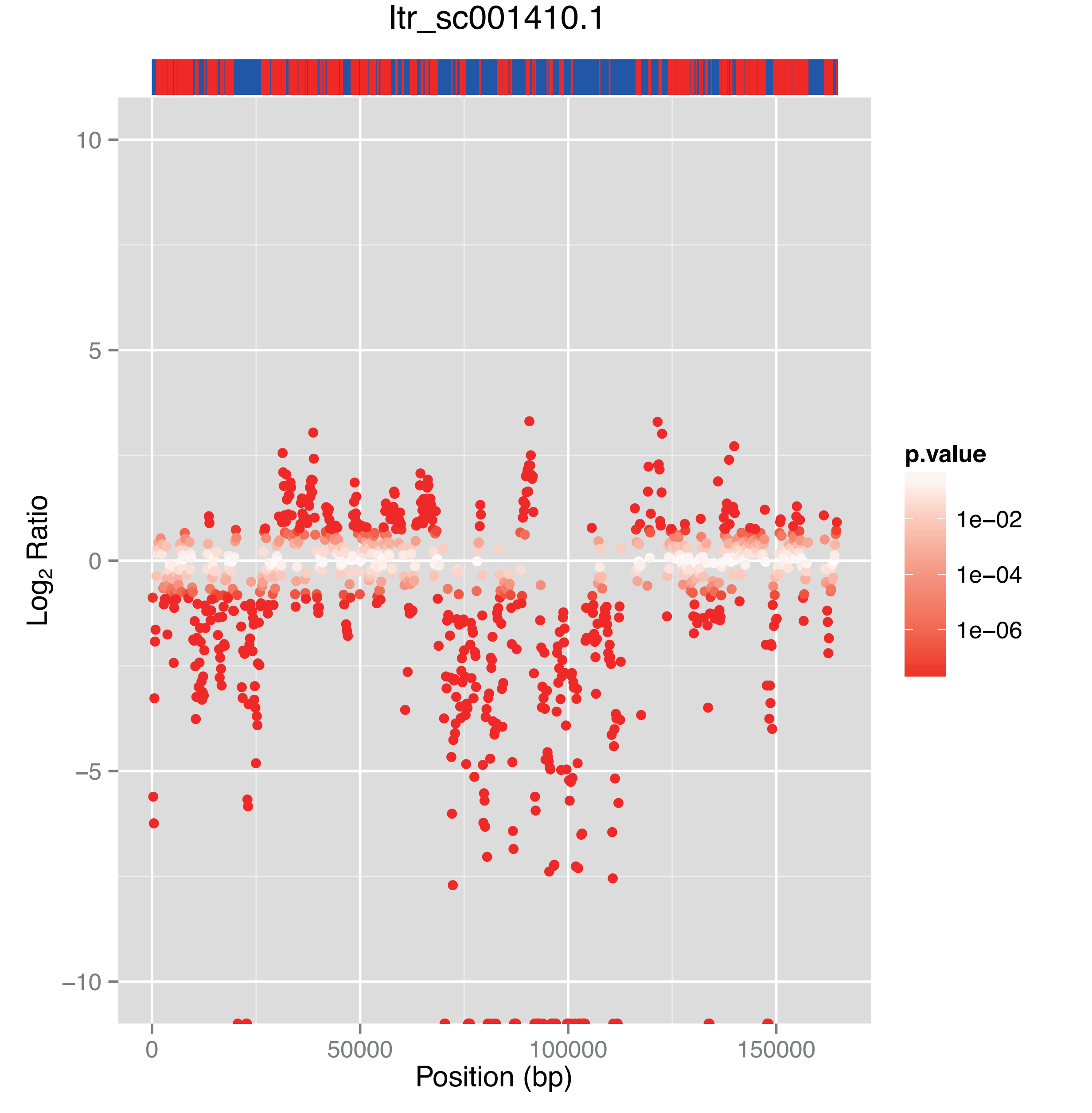
ltr_sc001410.1
10
5
0
−5
−10
0
50000
100000
150000
Position (bp)
Log2 Ratio
p.value
1e−02
1e−04
1e−06

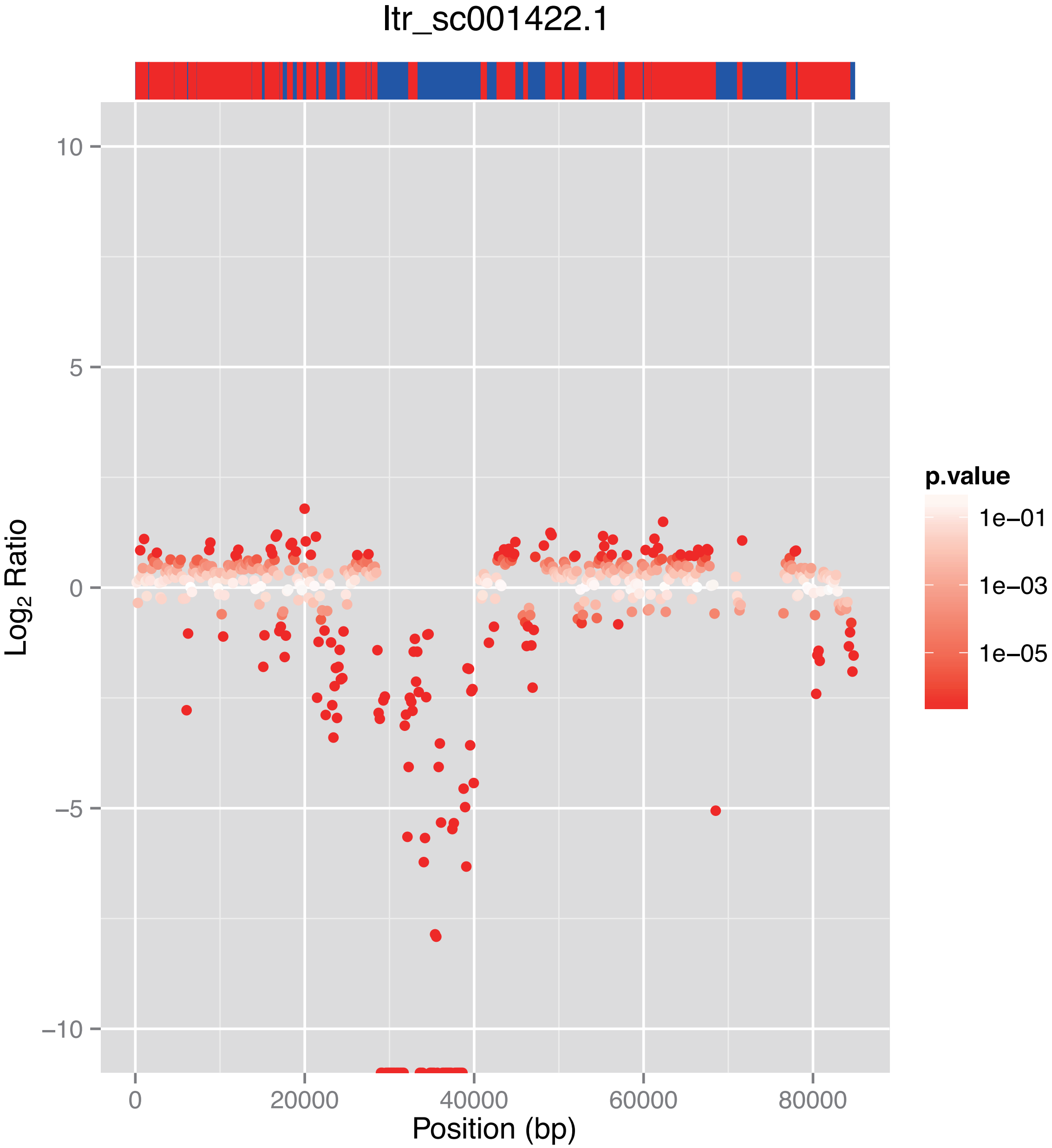
ltr_sc001422.1
10
5
0
−5
−10
Log2 Ratio
0
20000
40000
60000
80000
Position (bp)
p.value
1e−01
1e−03
1e−05

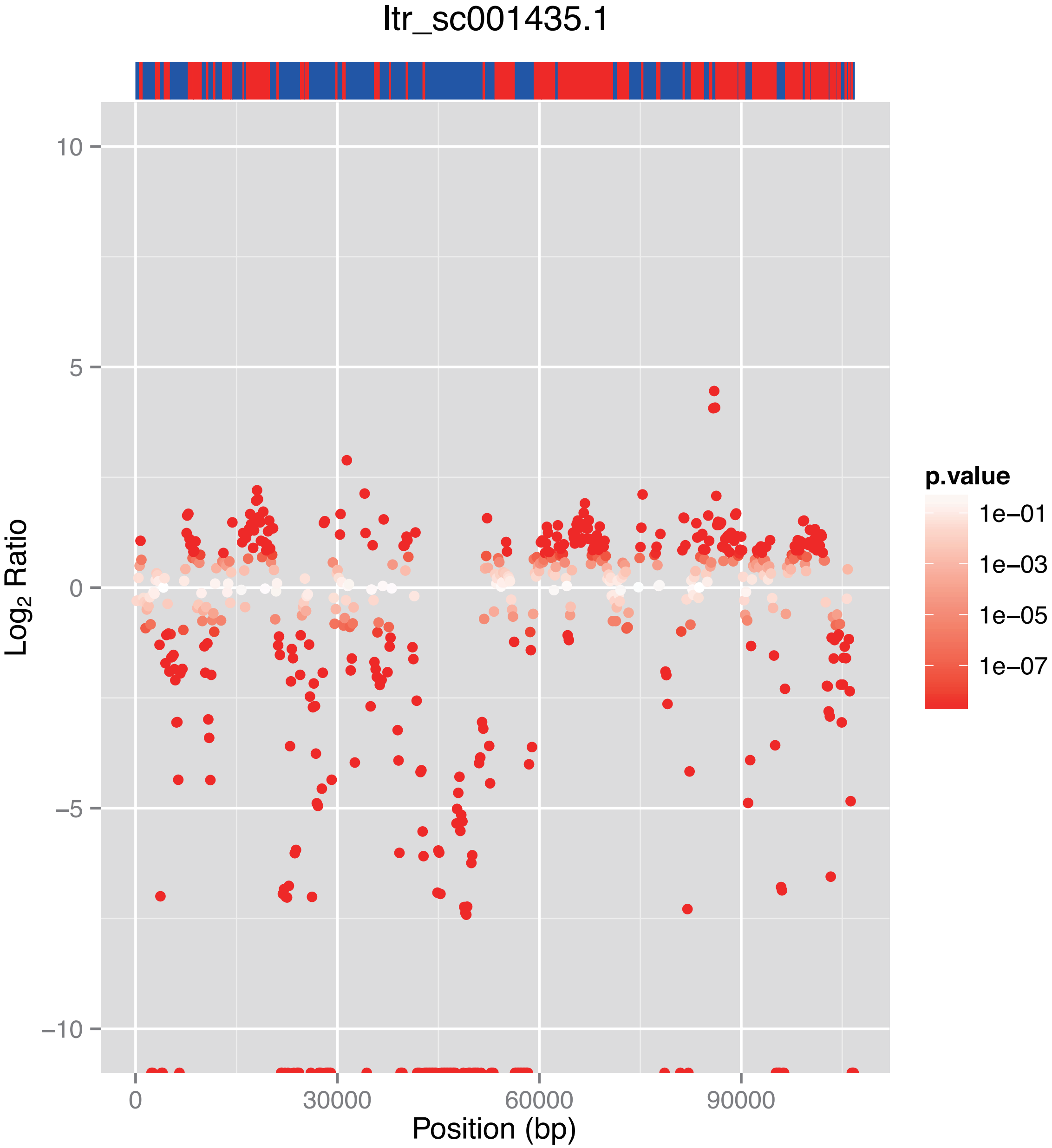
ltr_sc001435.1
Log2 Ratio
Position (bp)
p.value
1e−01
1e−03
1e−05
1e−07
10
5
0
−5
−10
0
30000
60000
90000

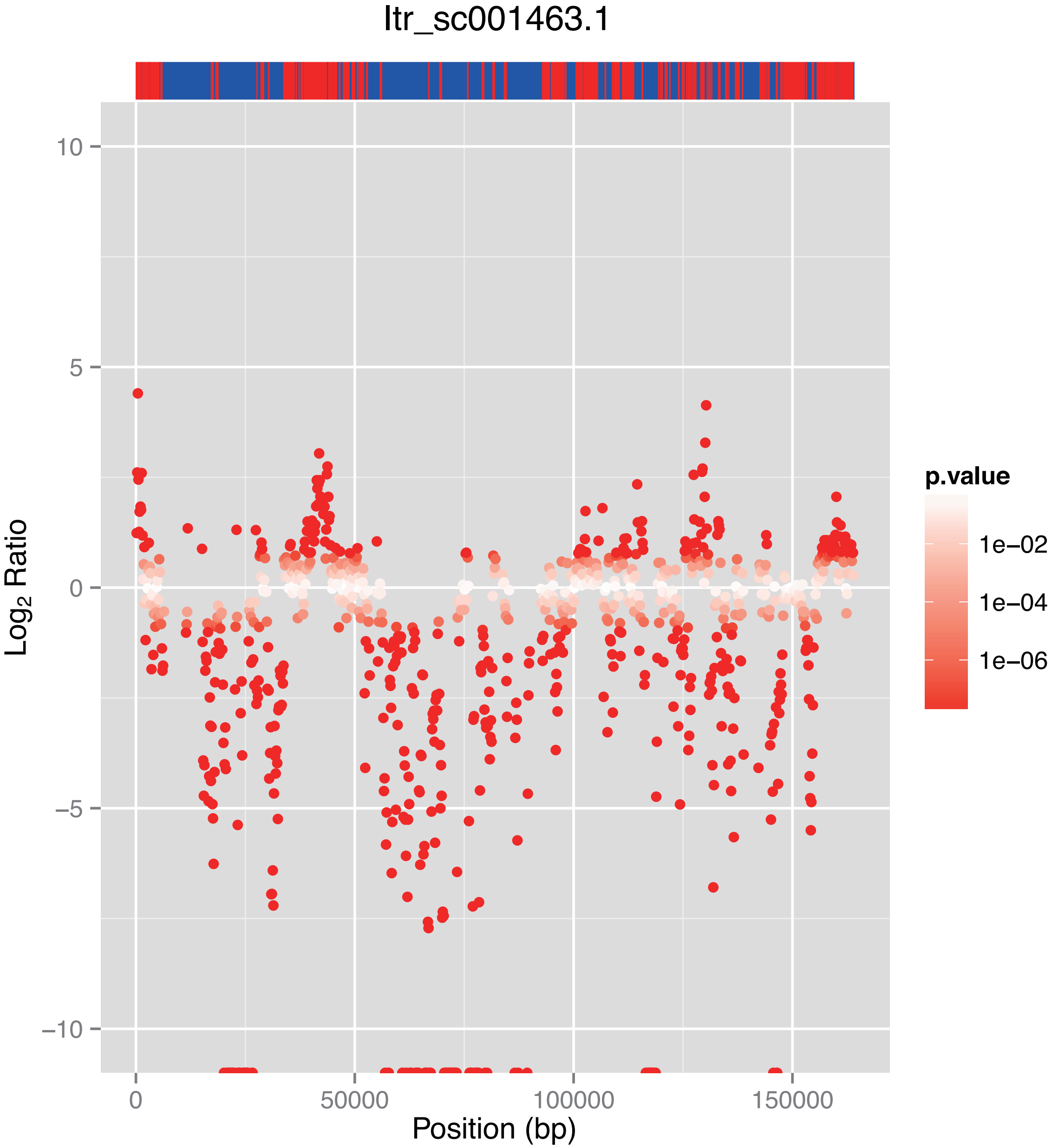
ltr_sc001463.1
10
5
0
−5
−10
Log2 Ratio
0
50000
100000
150000
Position (bp)
p.value
1e−02
1e−04
1e−06

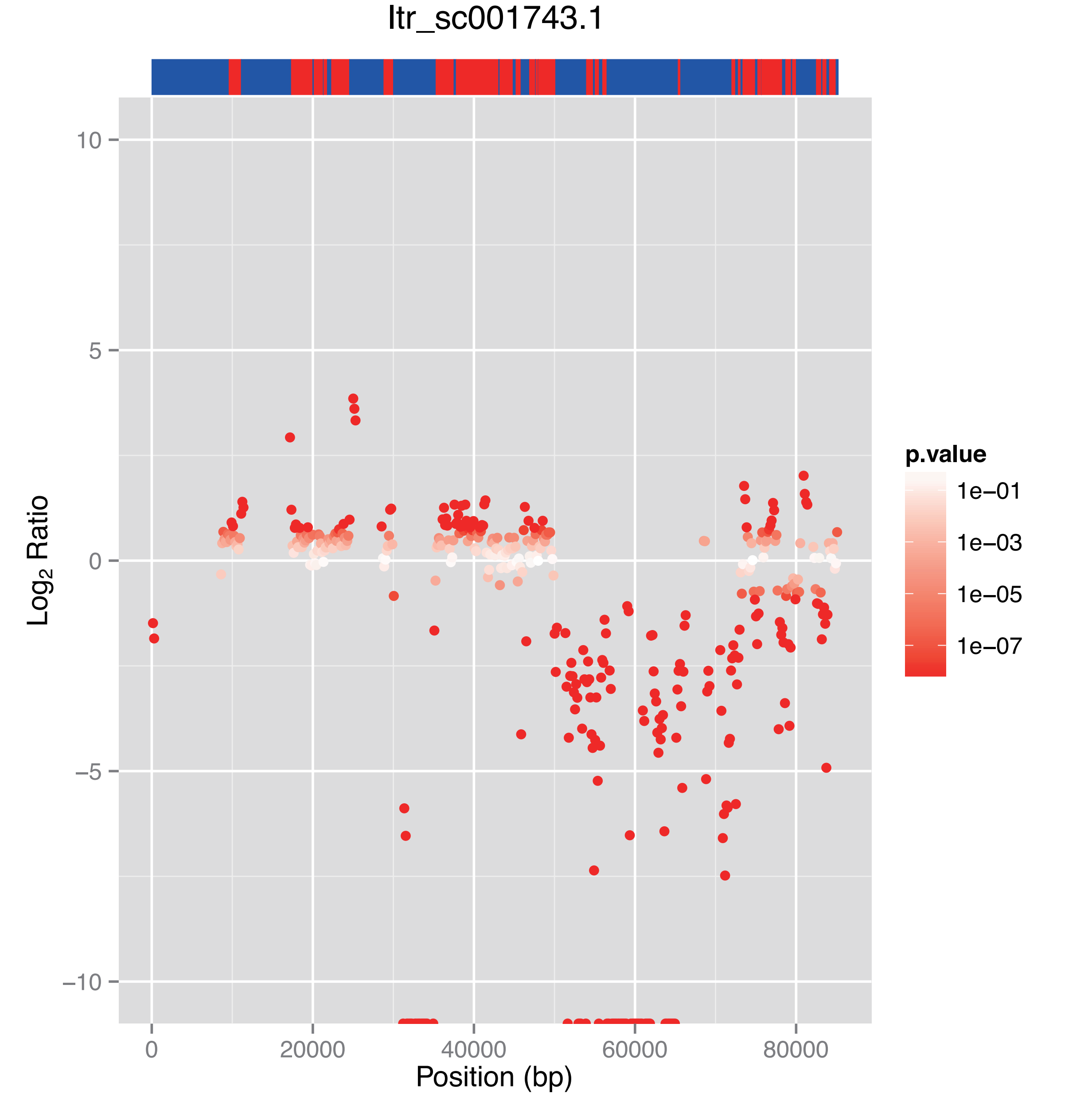
ltr_sc001743.1
10
5
0
−5
−10
Log2 Ratio
0
20000
40000
60000
80000
Position (bp)
p.value
1e−01
1e−03
1e−05
1e−07

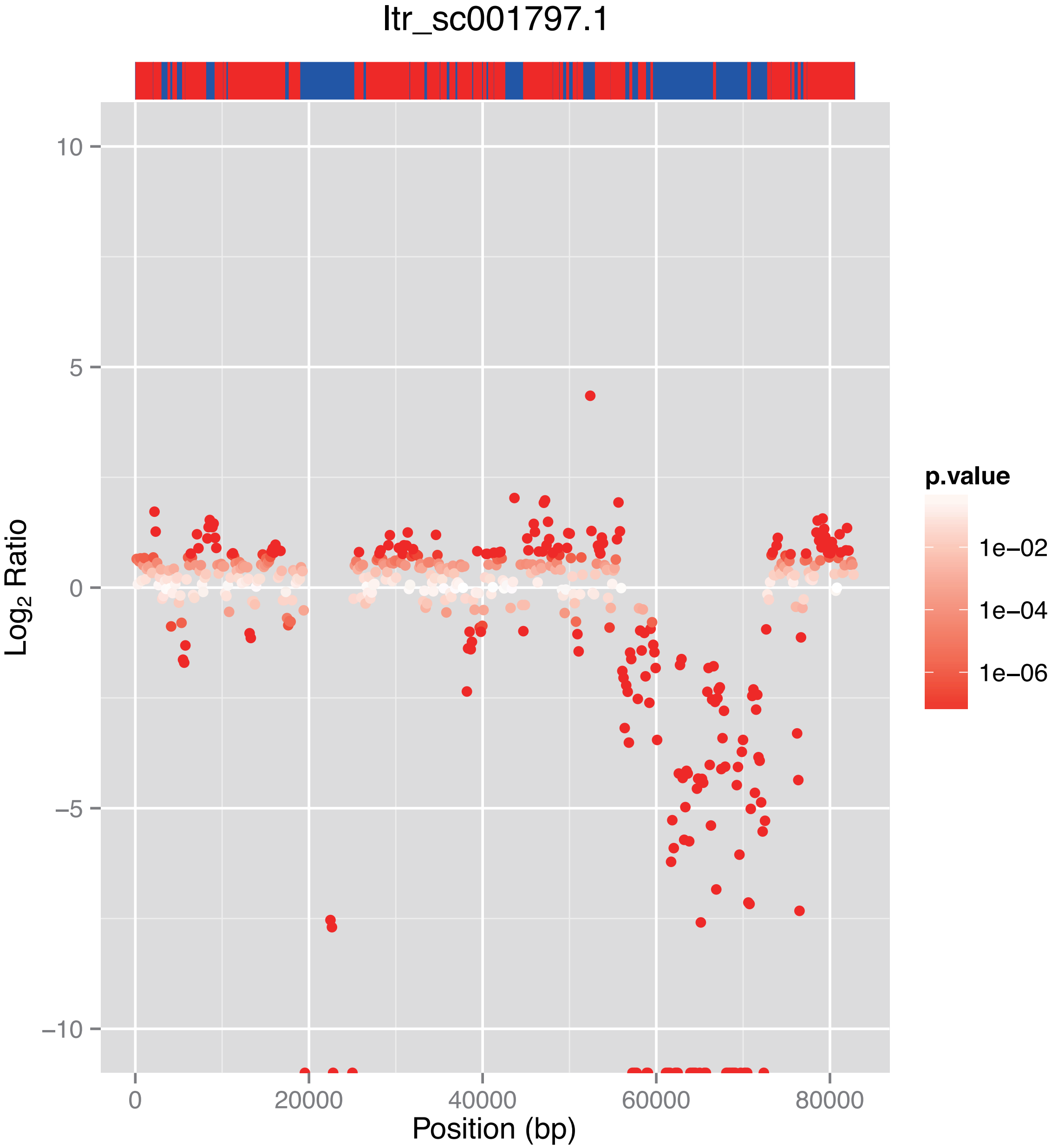

ltr_sc001797.1
Log2 Ratio
10
5
0
−5
−10
0
20000
40000
60000
80000
Position (bp)
p.value
1e−02
1e−04
1e−06

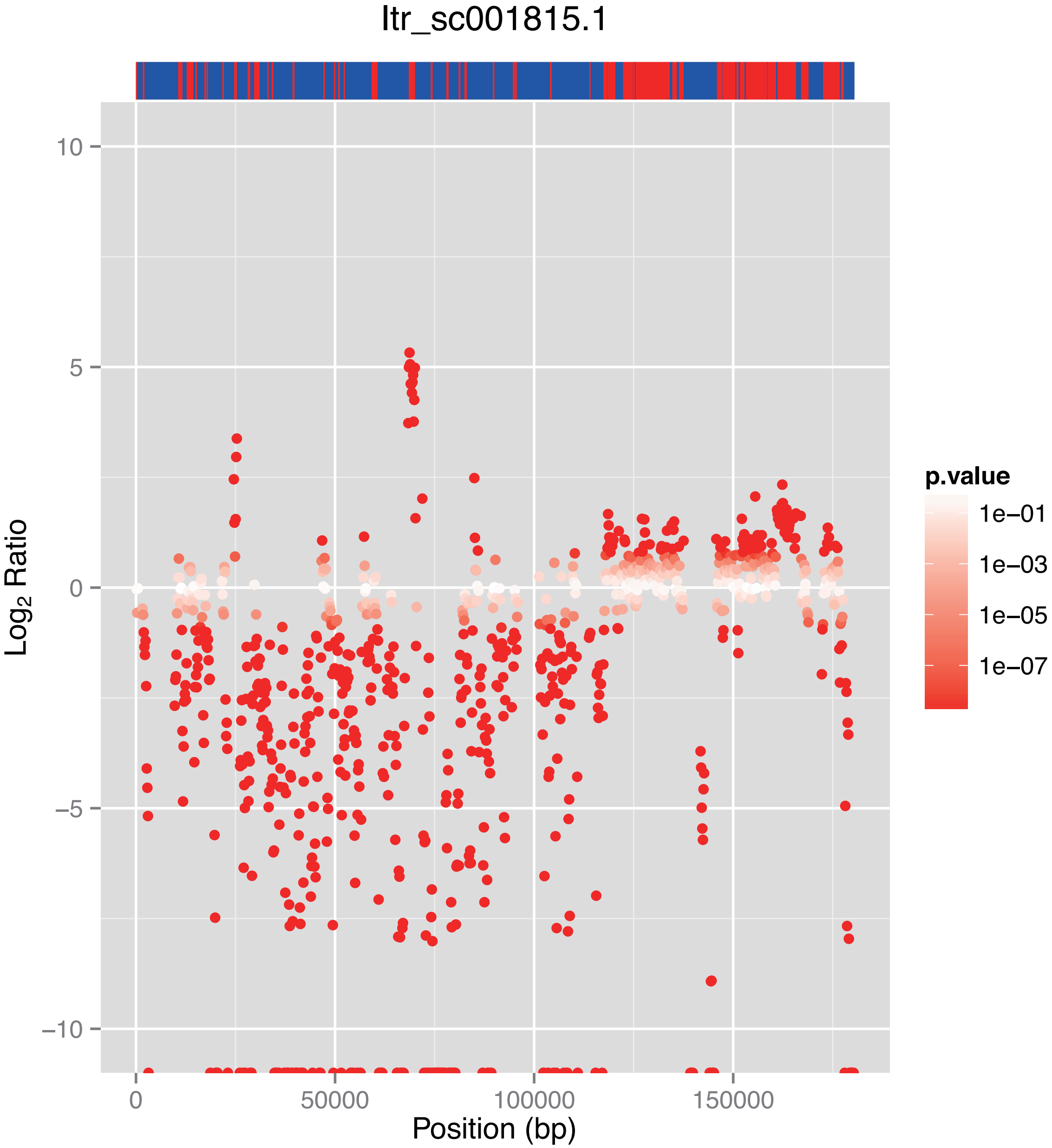
ltr_sc001815.1
Log2 Ratio
10
5
0
−5
−10
0
50000
100000
150000
Position (bp)
p.value
1e−01
1e−03
1e−05
1e−07

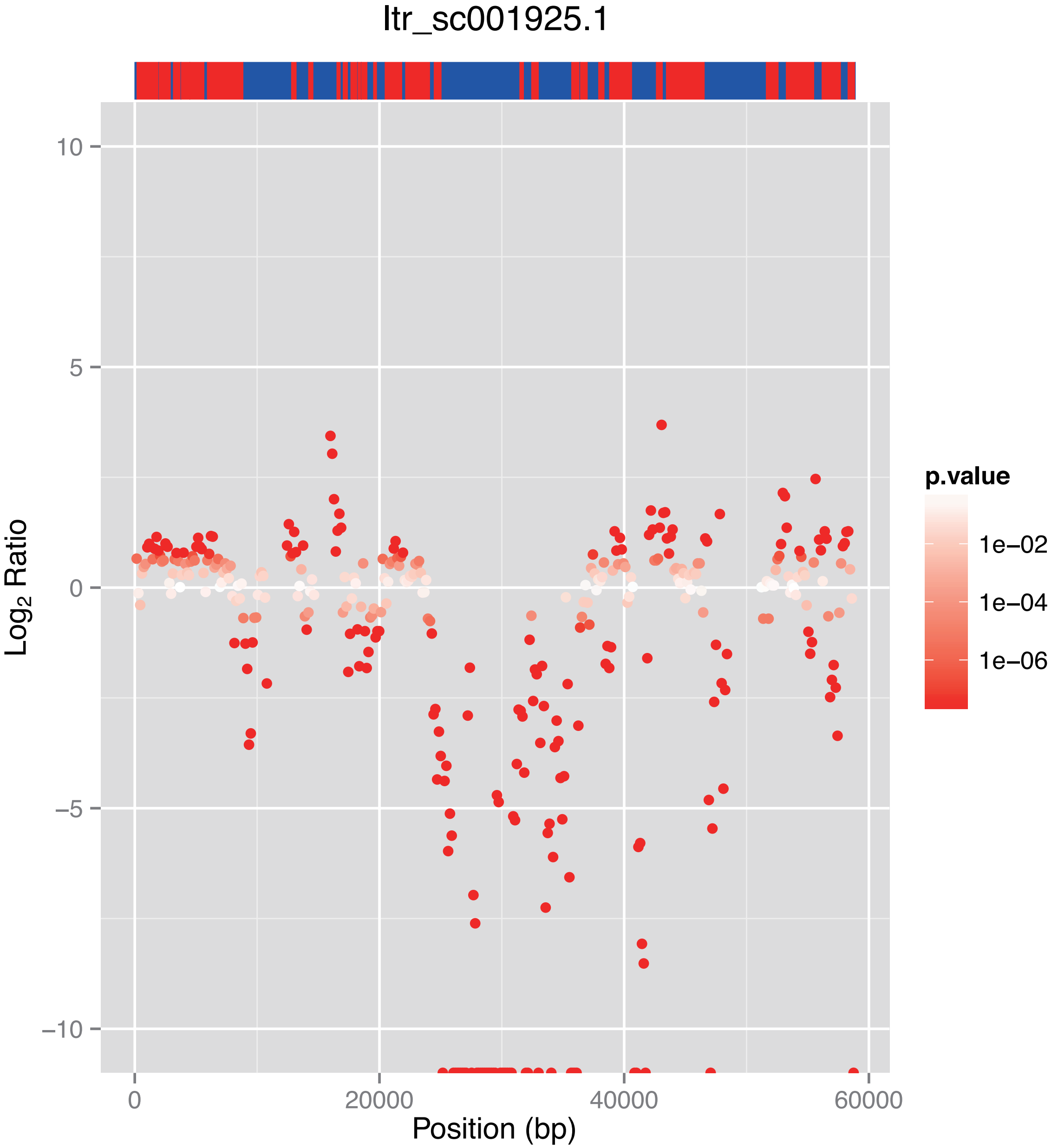
ltr_sc001925.1
Log2 Ratio
Position (bp)
p.value
1e−02
1e−04
1e−06
10
5
0
−5
−10
0
20000
40000
60000

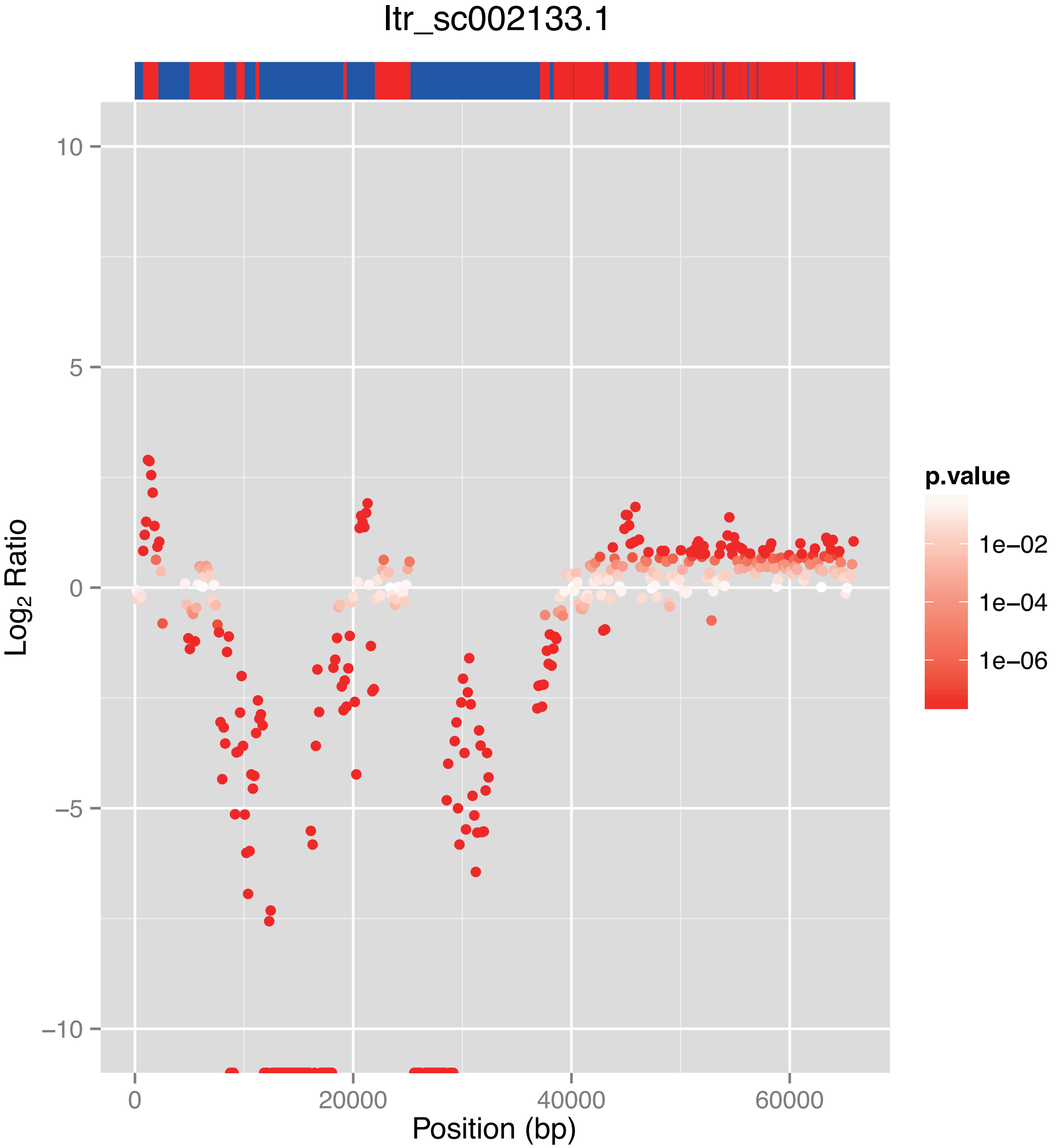
ltr_sc002133.1
10
5
0
−5
−10
Log2 Ratio
0
20000
40000
60000
Position (bp)
p.value
1e−02
1e−04
1e−06

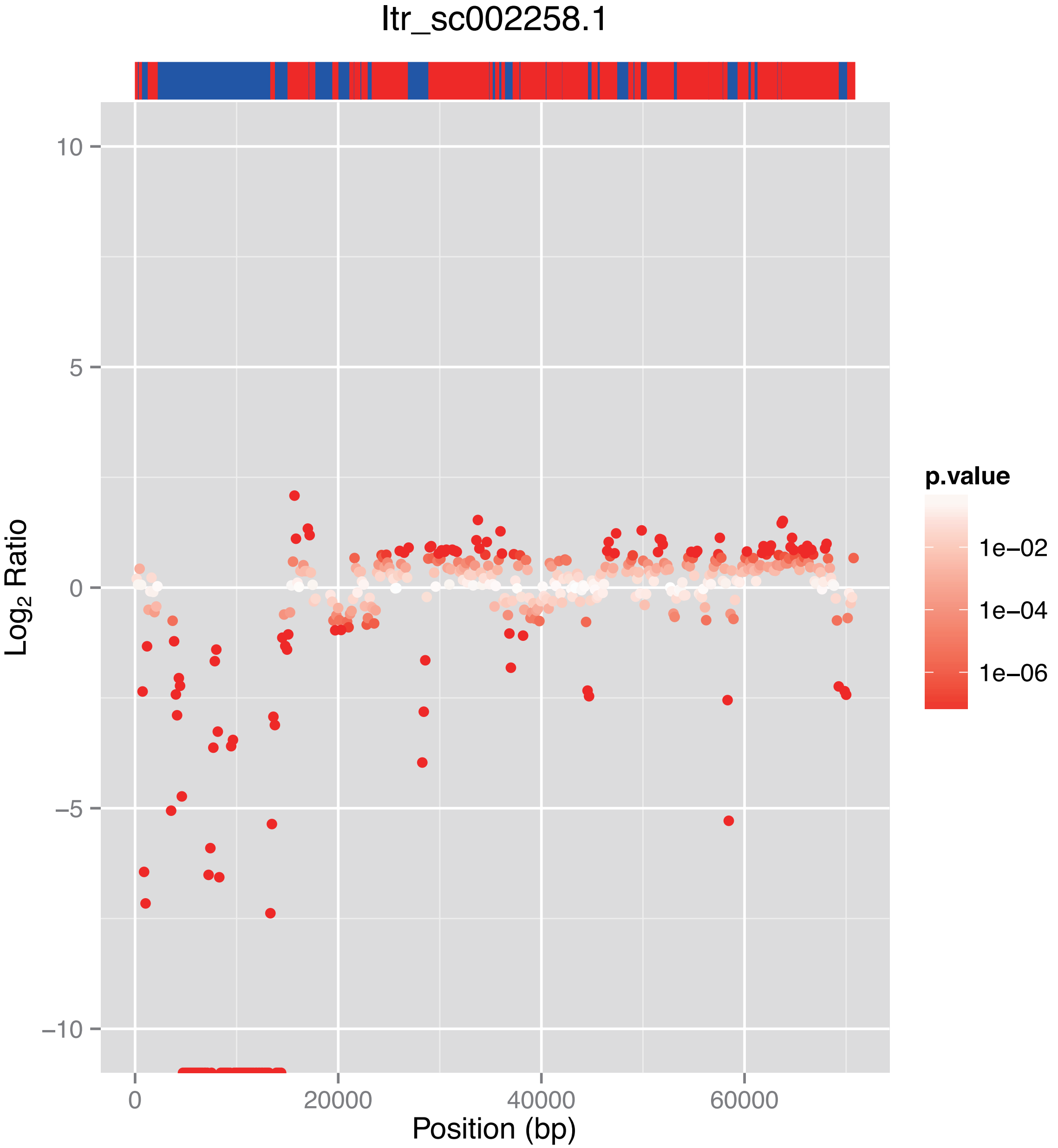

ltr_sc002258.1
Log2 Ratio
10
5
0
−5
−10
0
20000
40000
60000
Position (bp)
p.value
1e−02
1e−04
1e−06

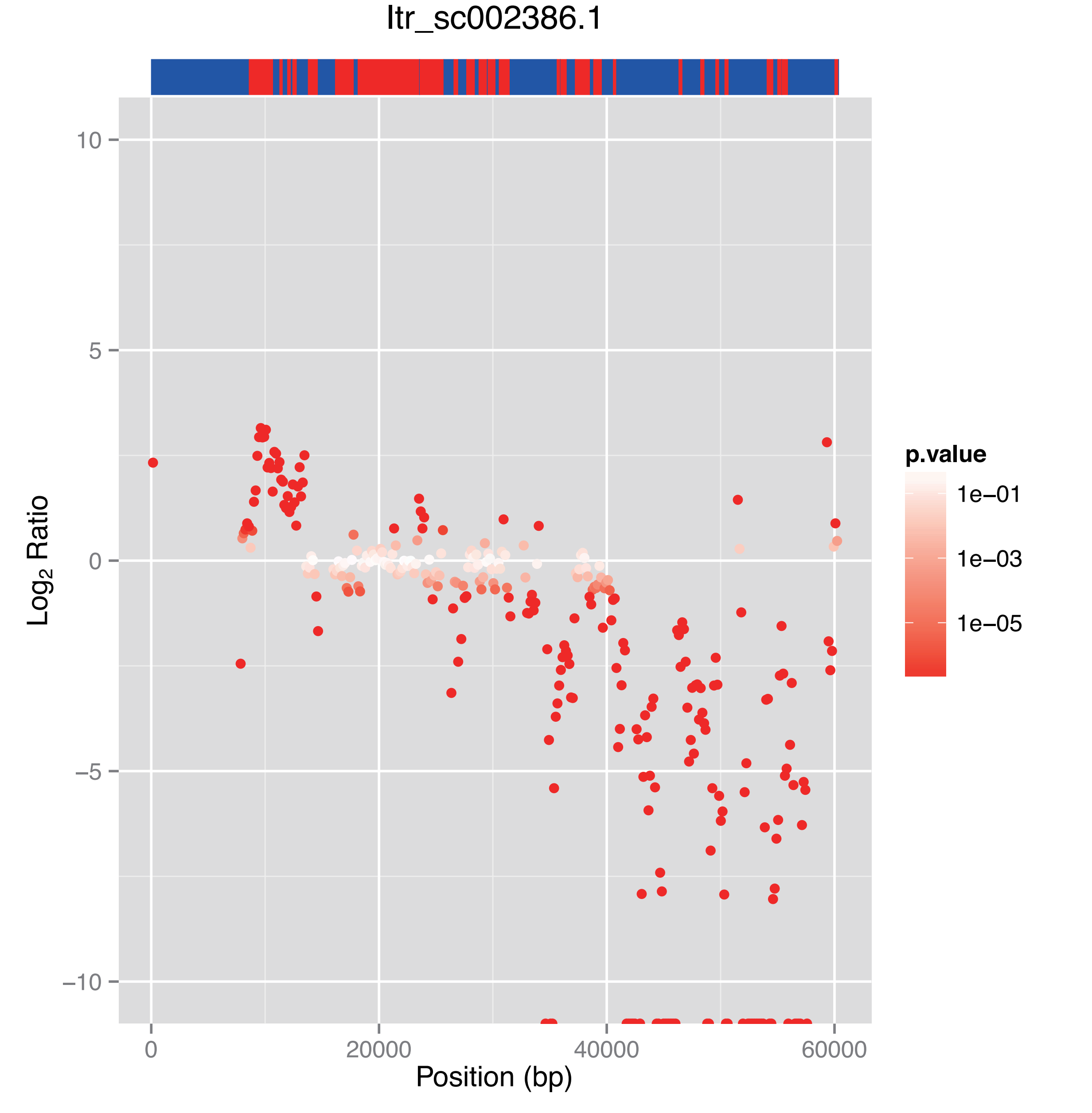

ltr_sc002386.1
Log2 Ratio
10
5
0
−5
−10
0
20000
40000
60000
Position (bp)
p.value
1e−01
1e−03
1e−05

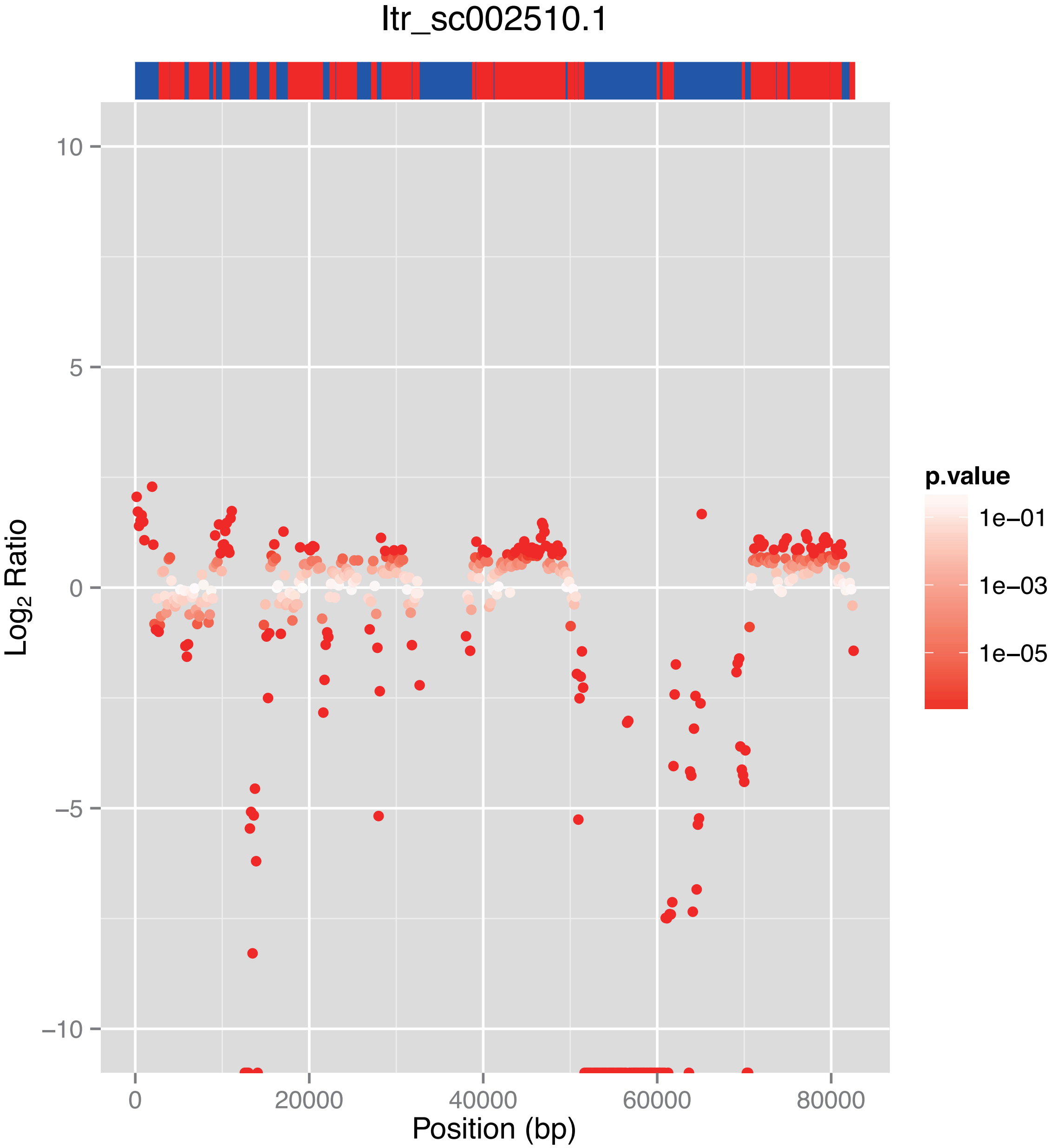
ltr_sc002510.1
Log$_2$ Ratio
10
5
0
−5
−10
0
20000
40000
60000
80000
Position (bp)
p.value
1e−01
1e−03
1e−05

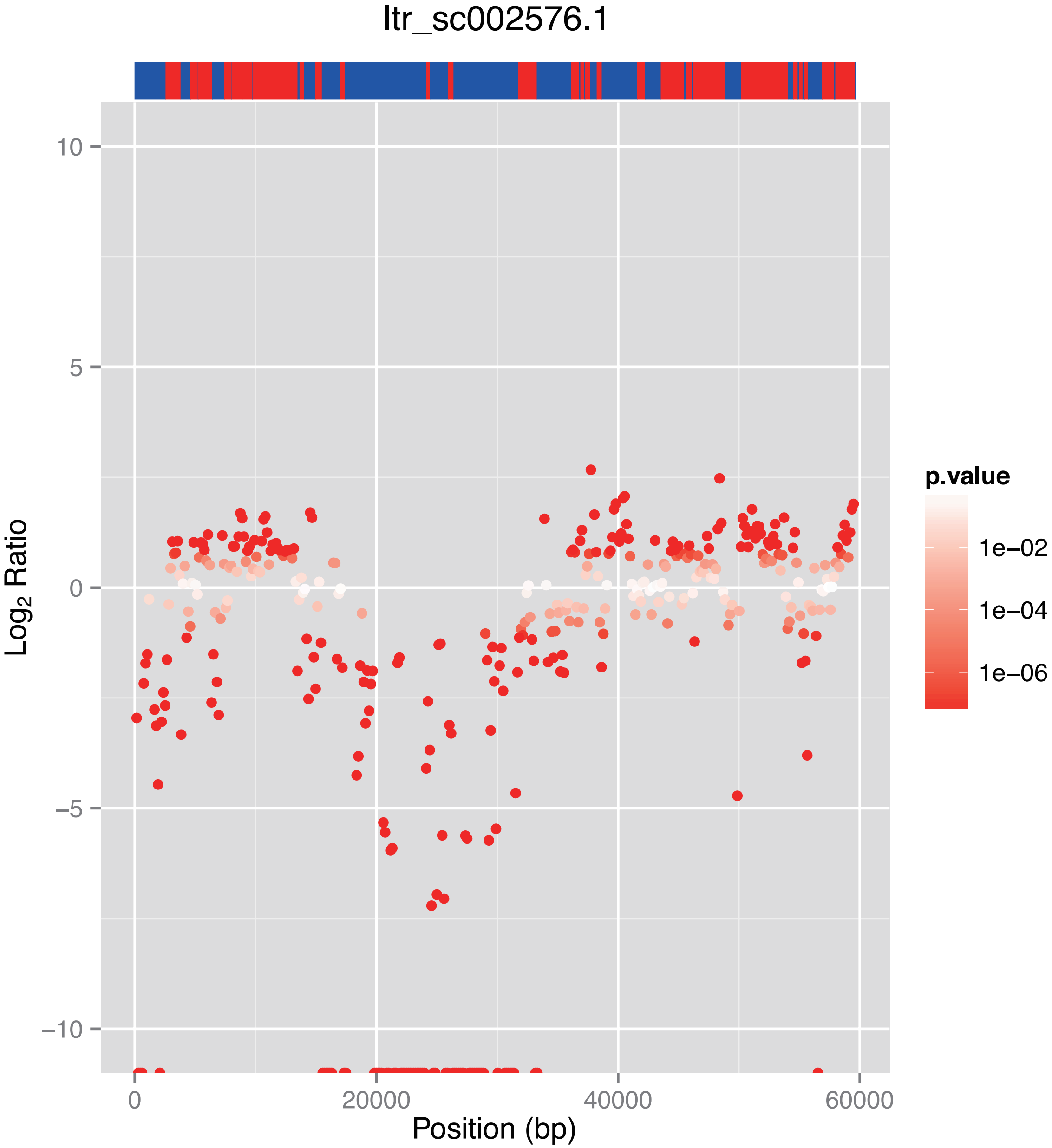
ltr_sc002576.1
10
5
0
−5
−10
Log$_2$ Ratio
0
20000
40000
60000
Position (bp)
p.value
1e−02
1e−04
1e−06

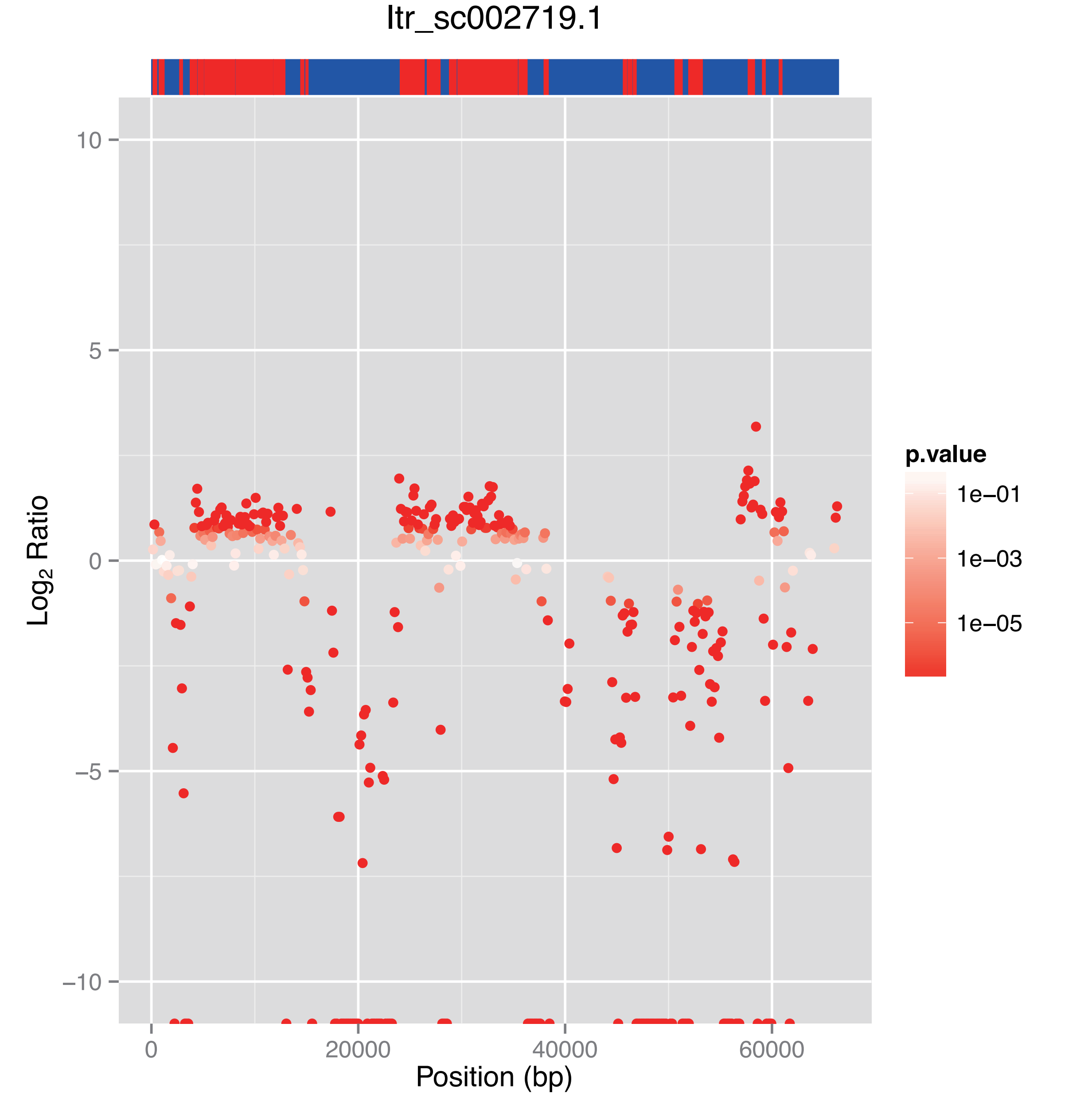
ltr_sc002719.1
10
5
0
−5
−10
Log2 Ratio
0
20000
40000
60000
Position (bp)
p.value
1e−01
1e−03
1e−05
