# Supplementary material for: Survey of genome sequences in a wild sweet potato, Ipomoea trifida (H. B. K.) G. Don
Source: DNA Res. 2015 Mar 24;22(2):171–9. doi: 10.1093/dnares/dsv002 (PMC4401327; doi:10.1093/dnares/dsv002)

## Slide 1
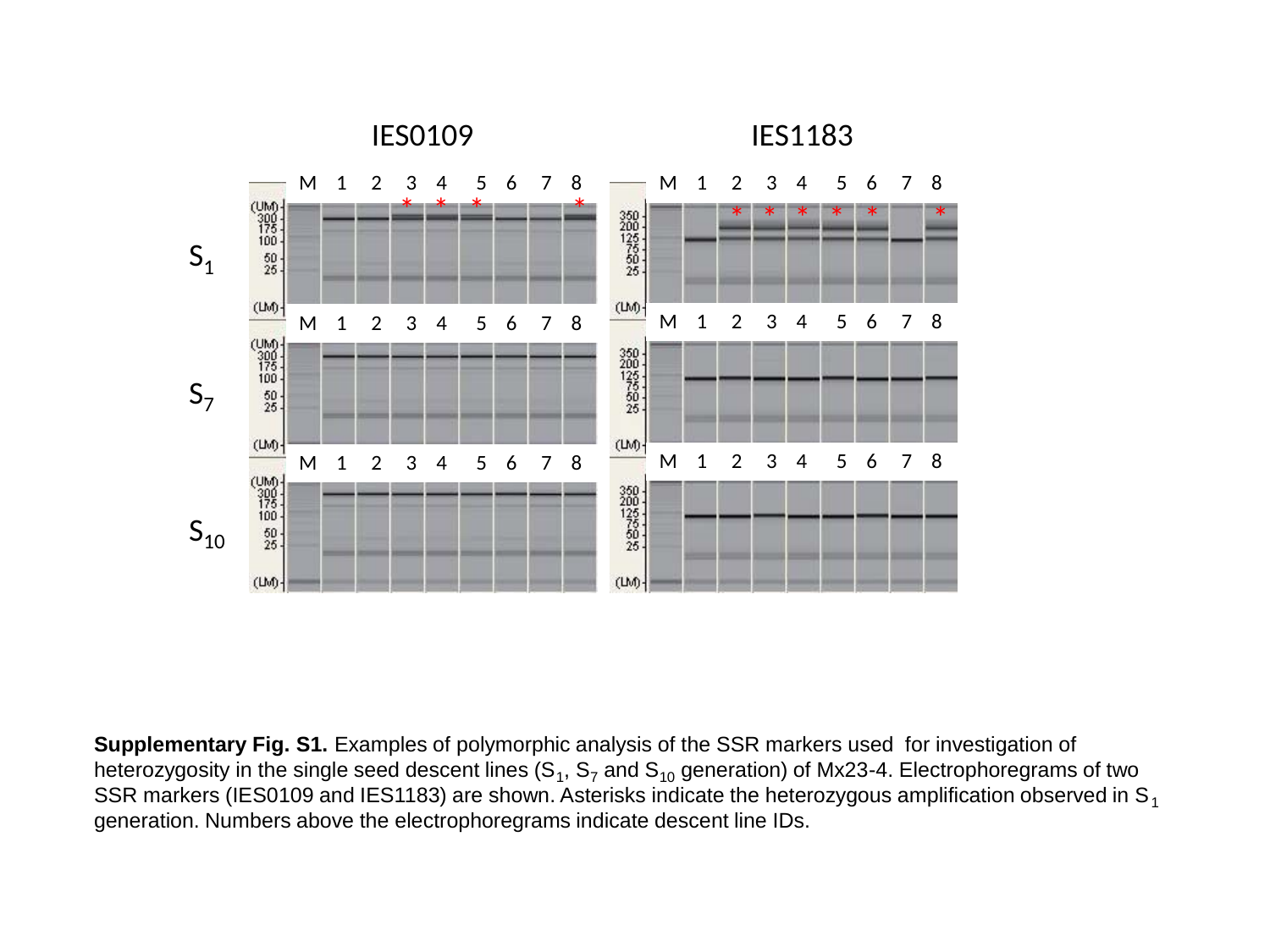

## Slide 2
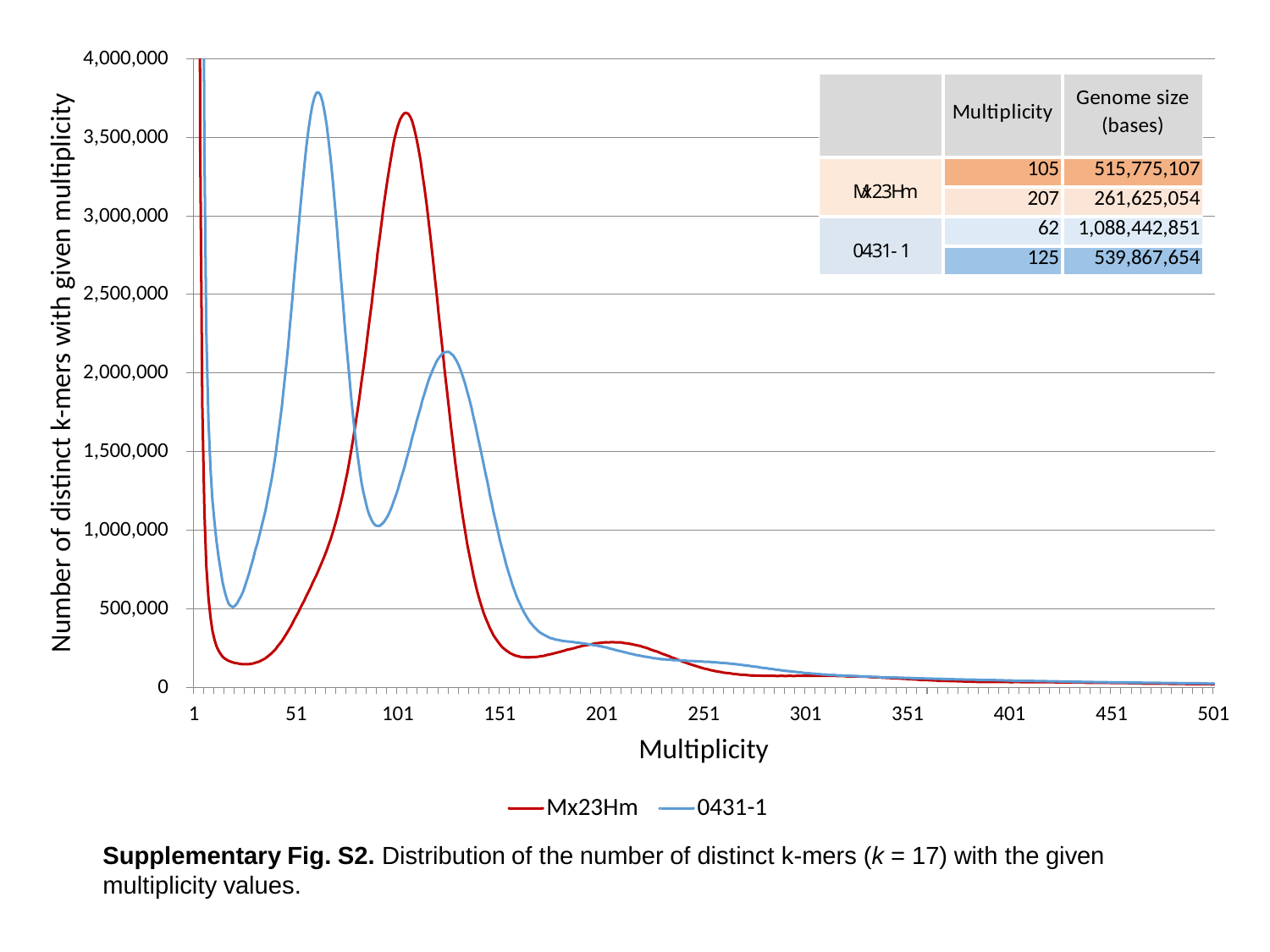

## Slide 3
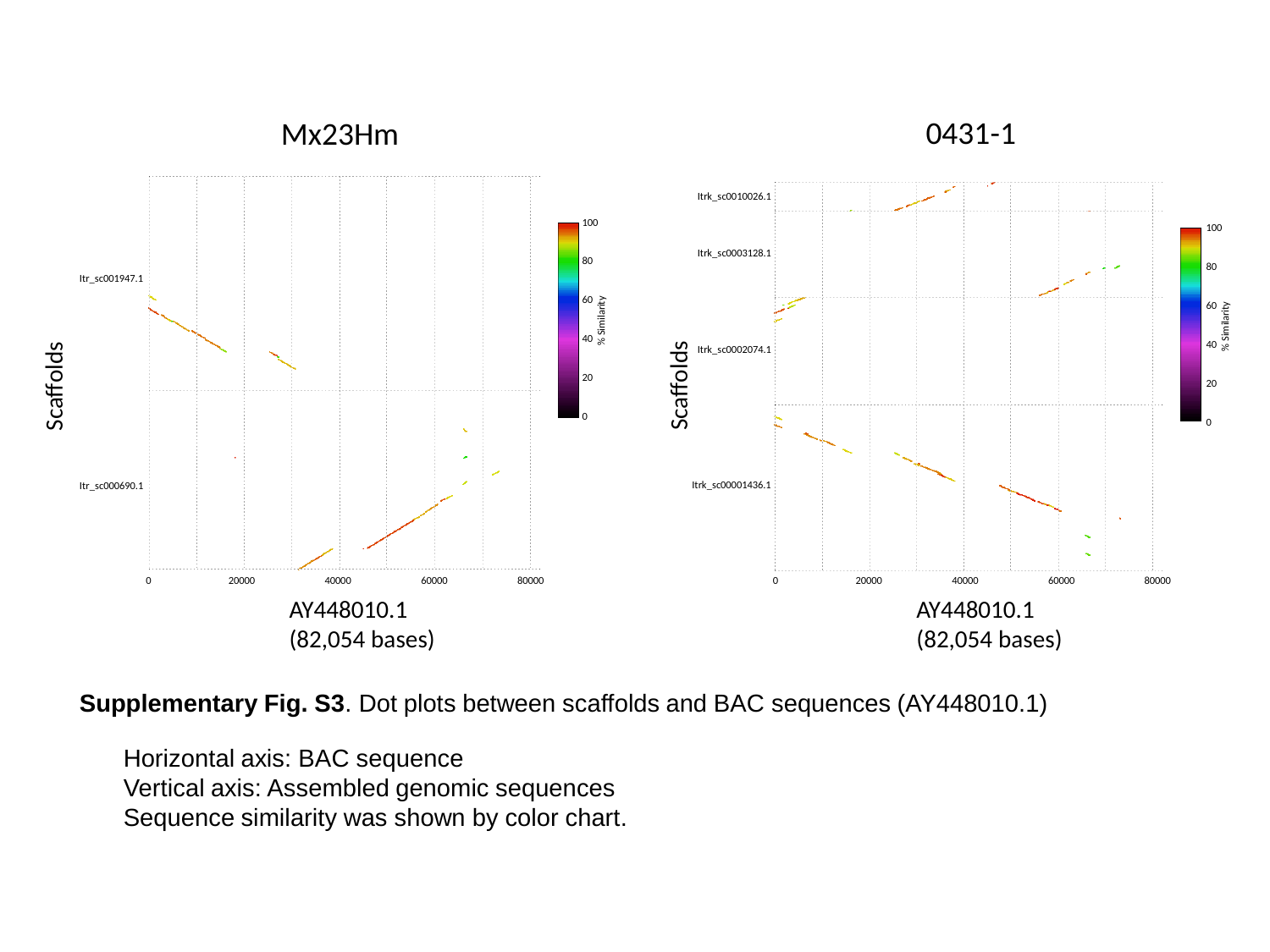

## Slide 4
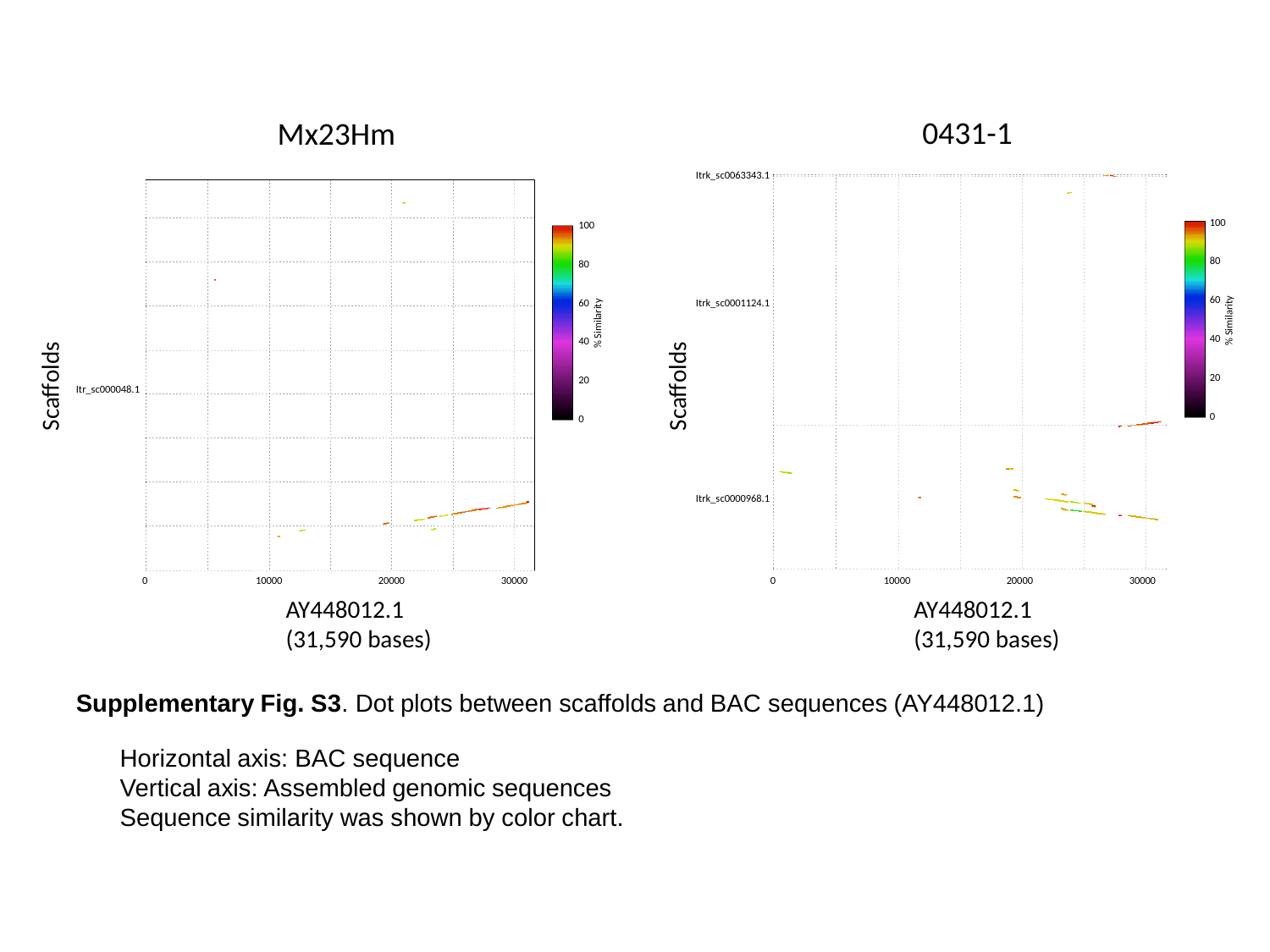

## Slide 5
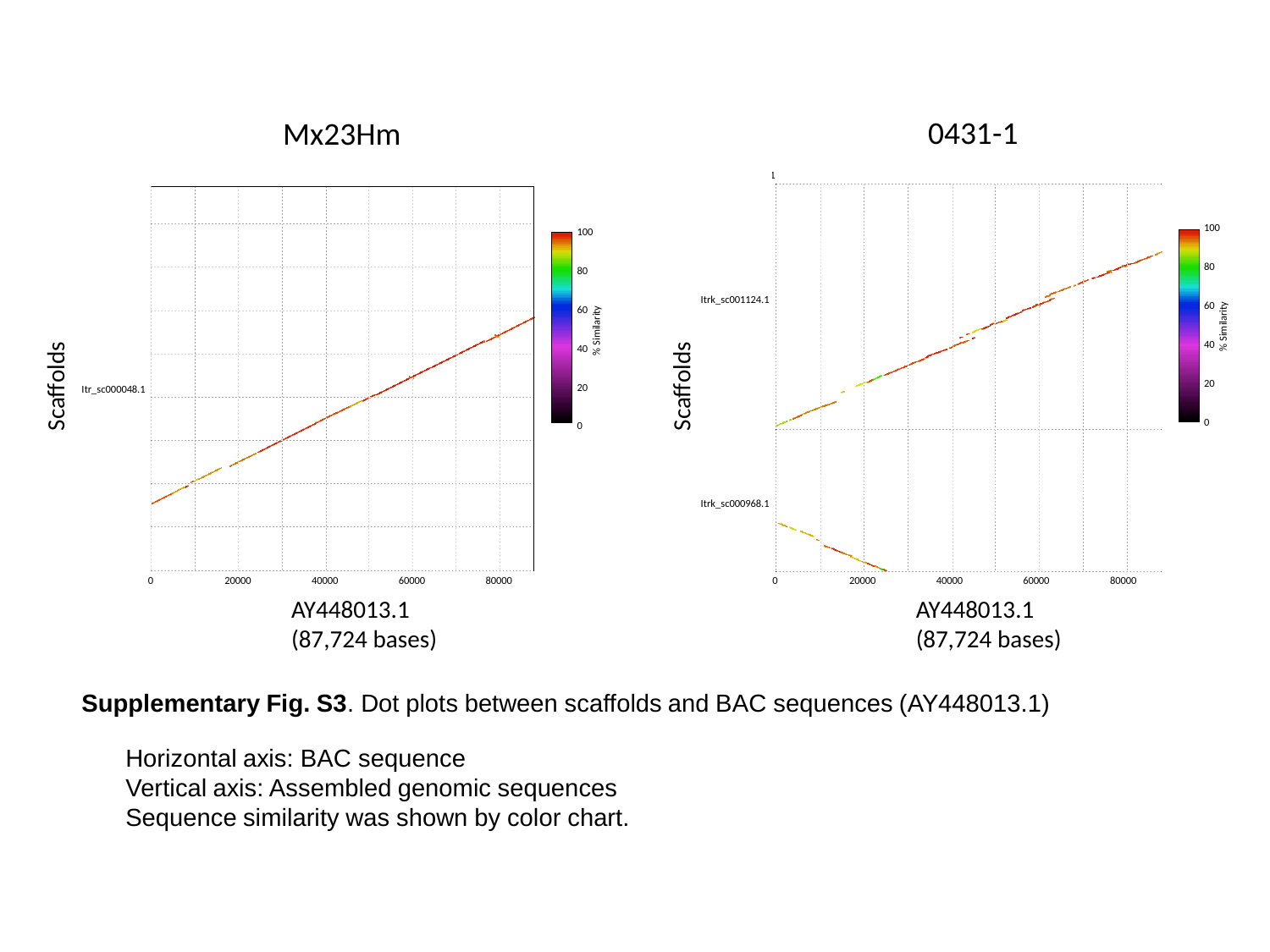

## Slide 6
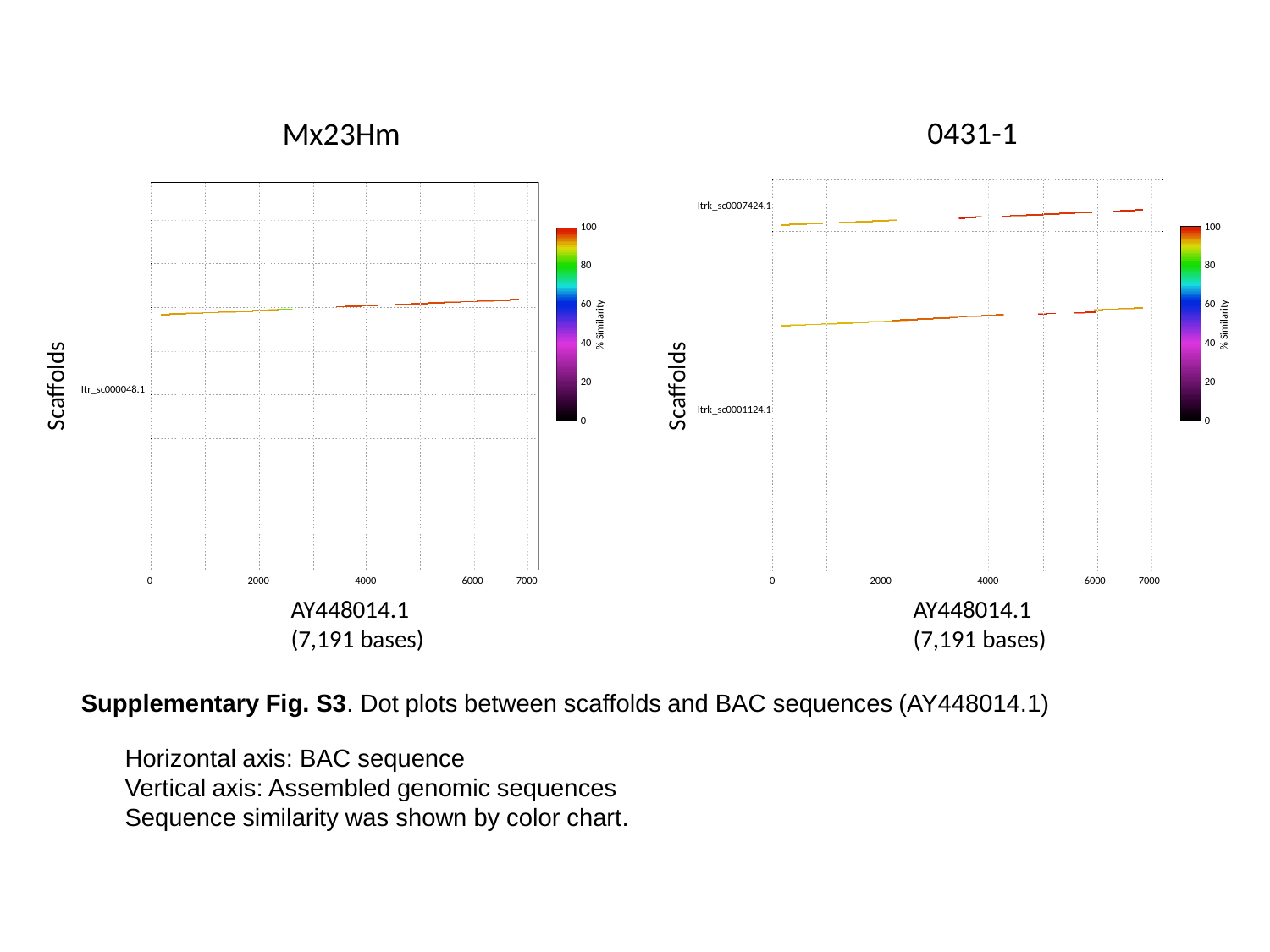

## Slide 7
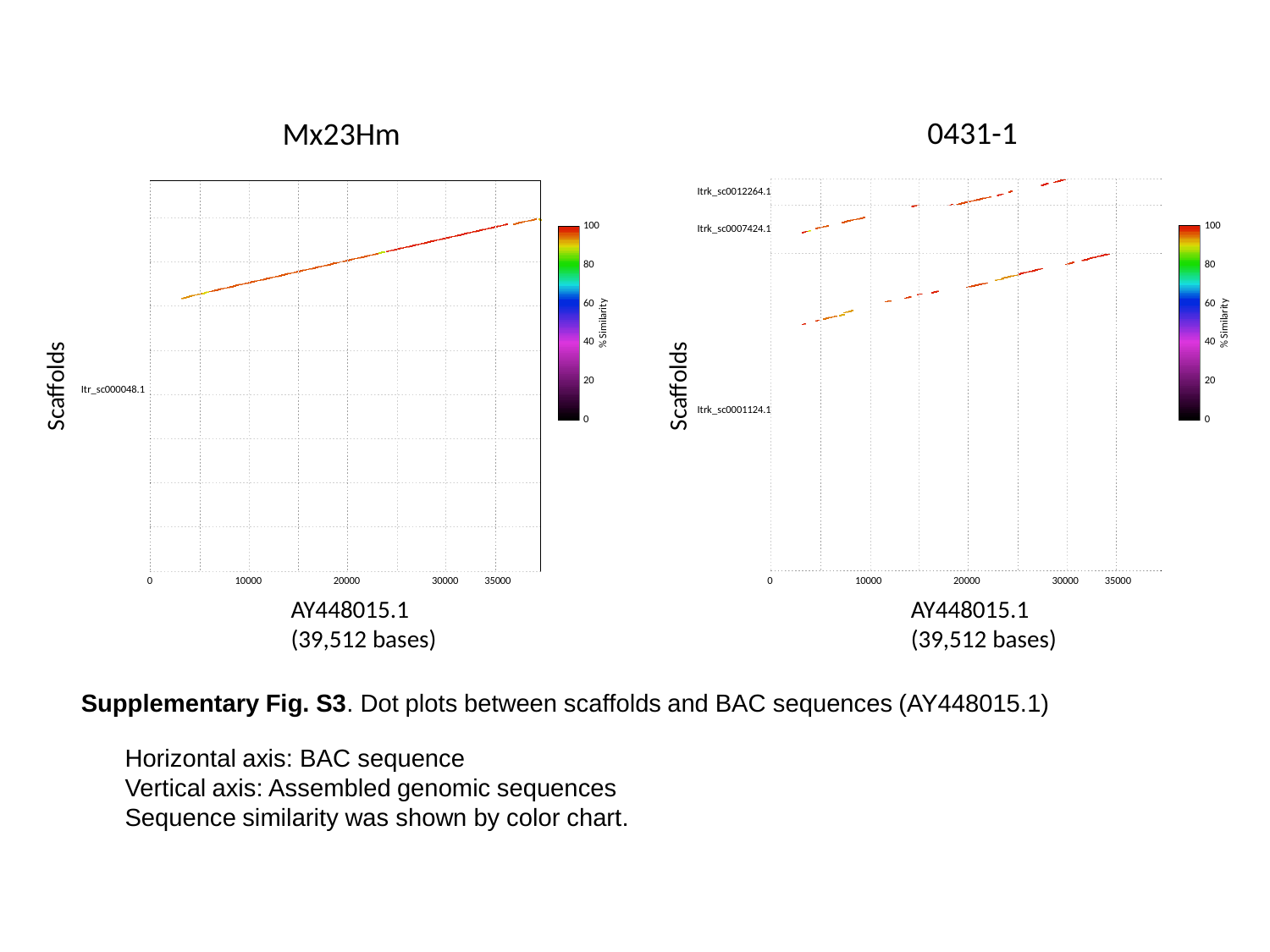

## Slide 8
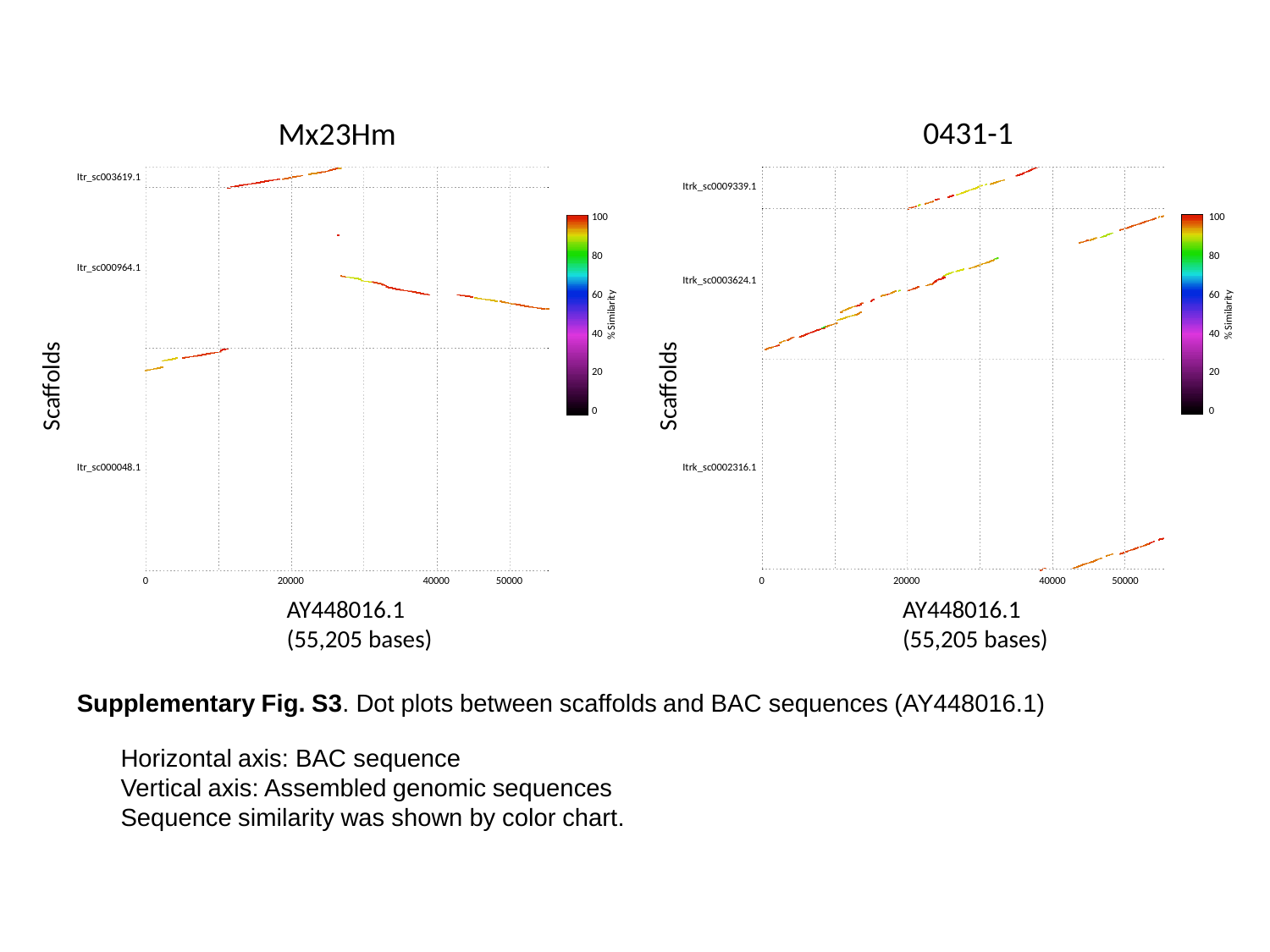

## Slide 9
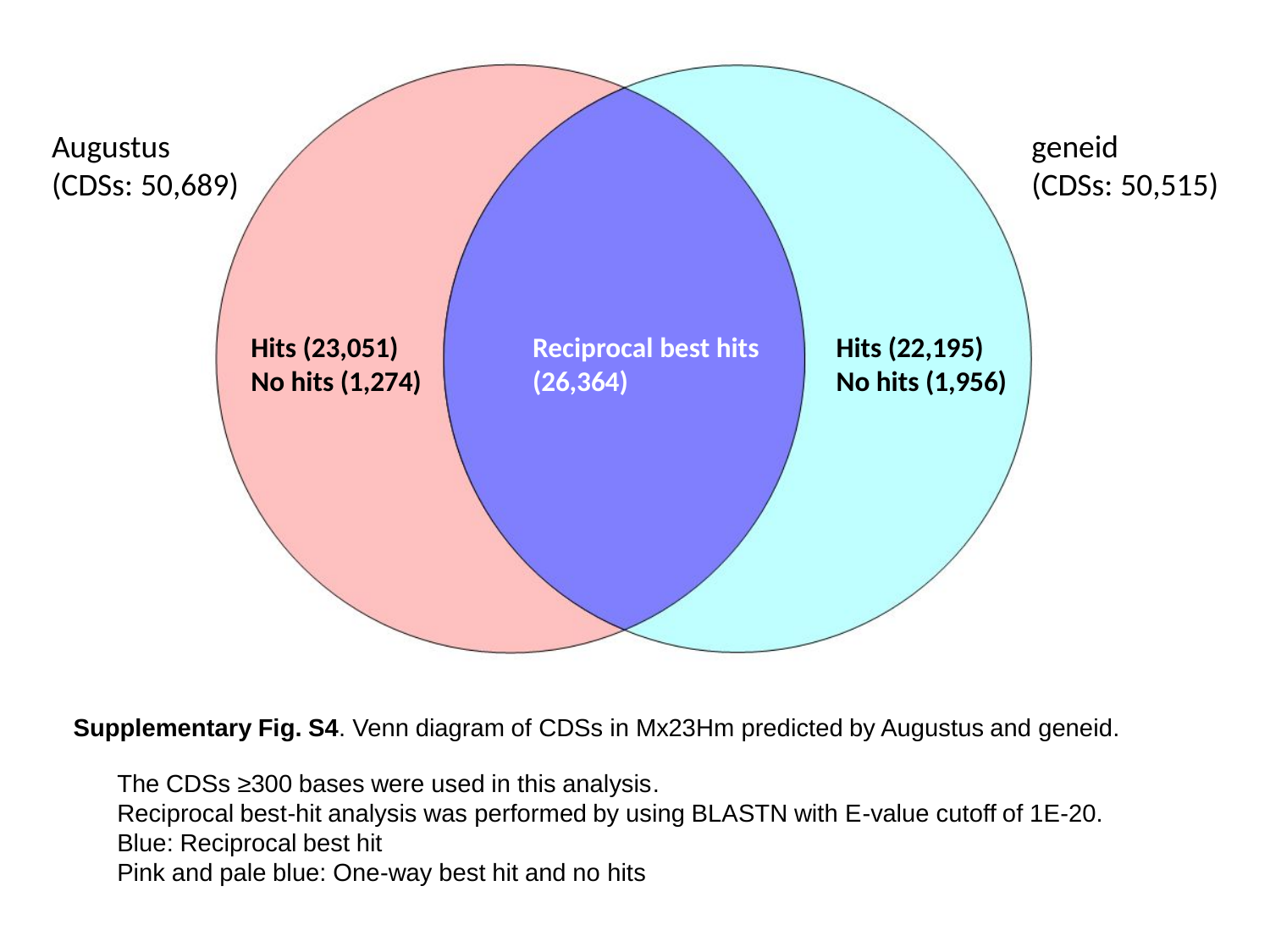

## Slide 10
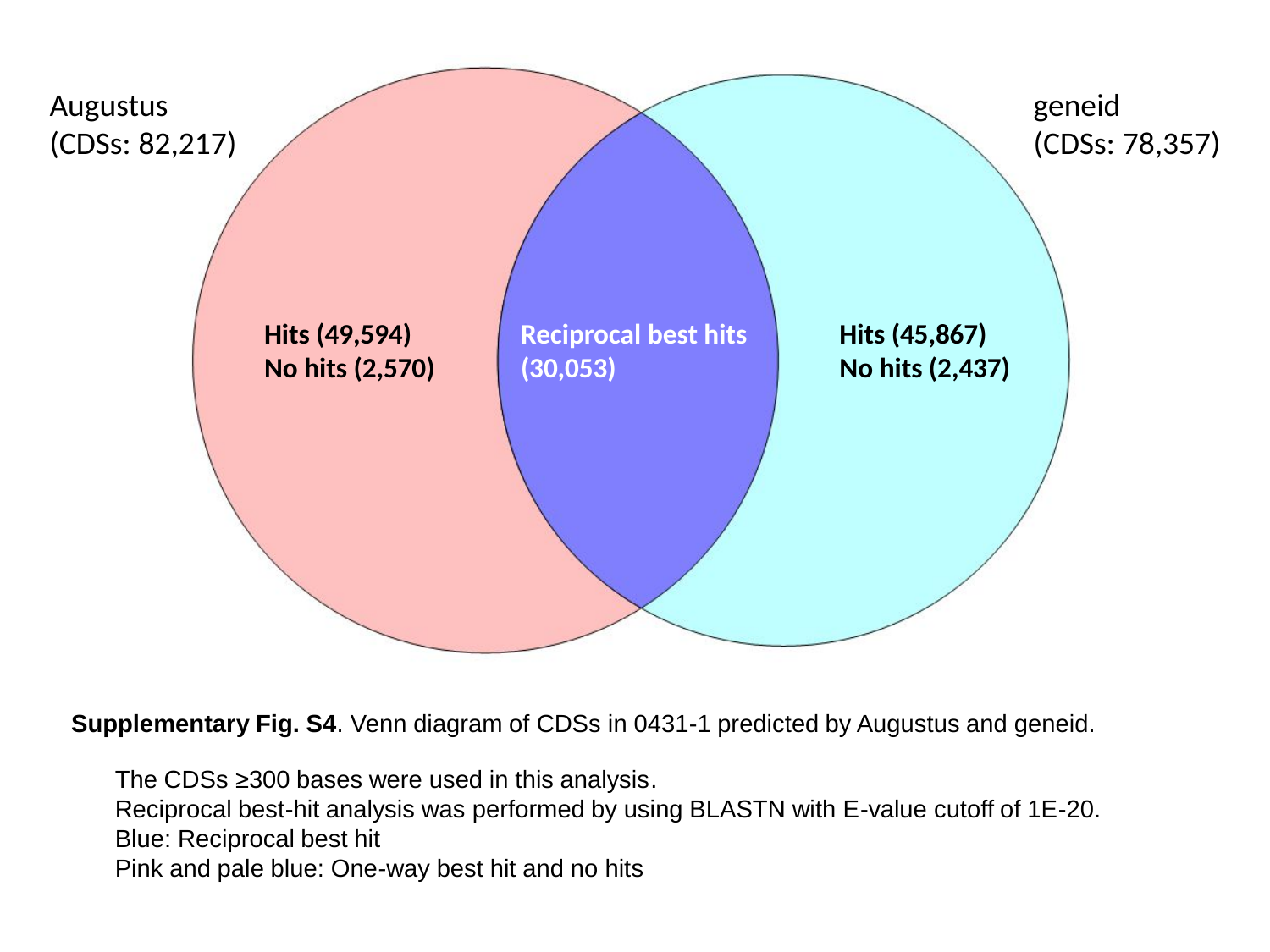

## Slide 11
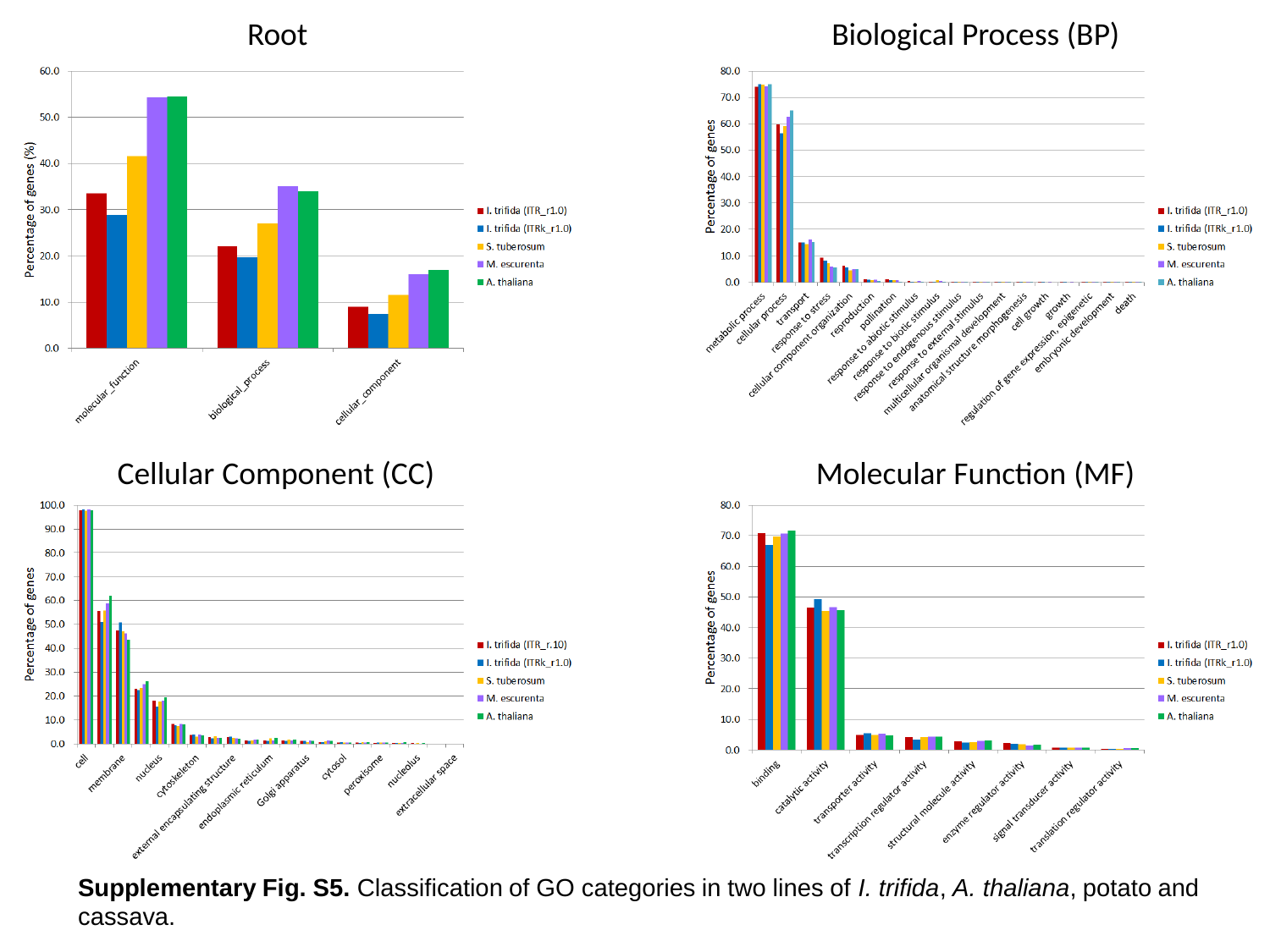

## Slide 12
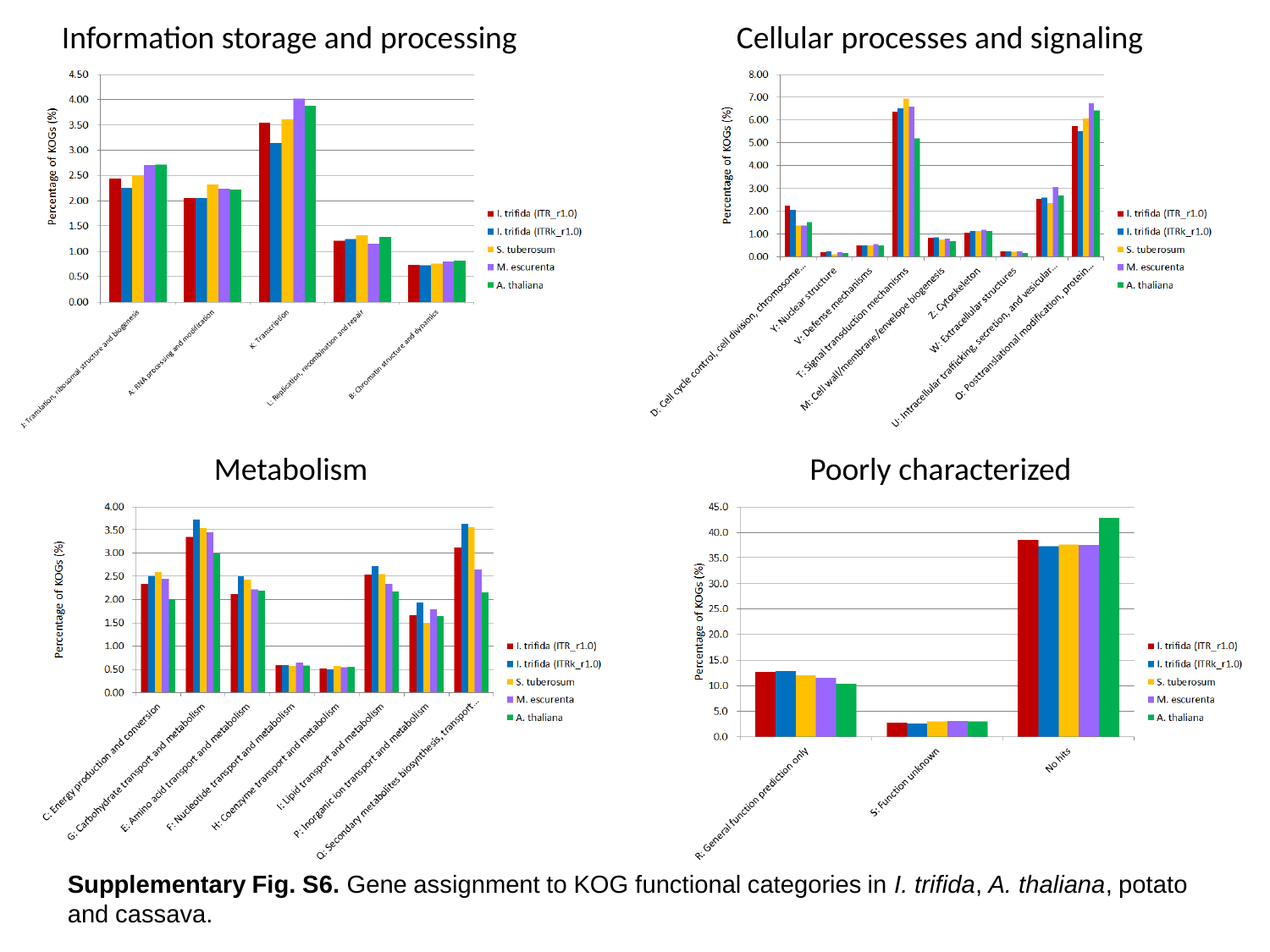

## Slide 13
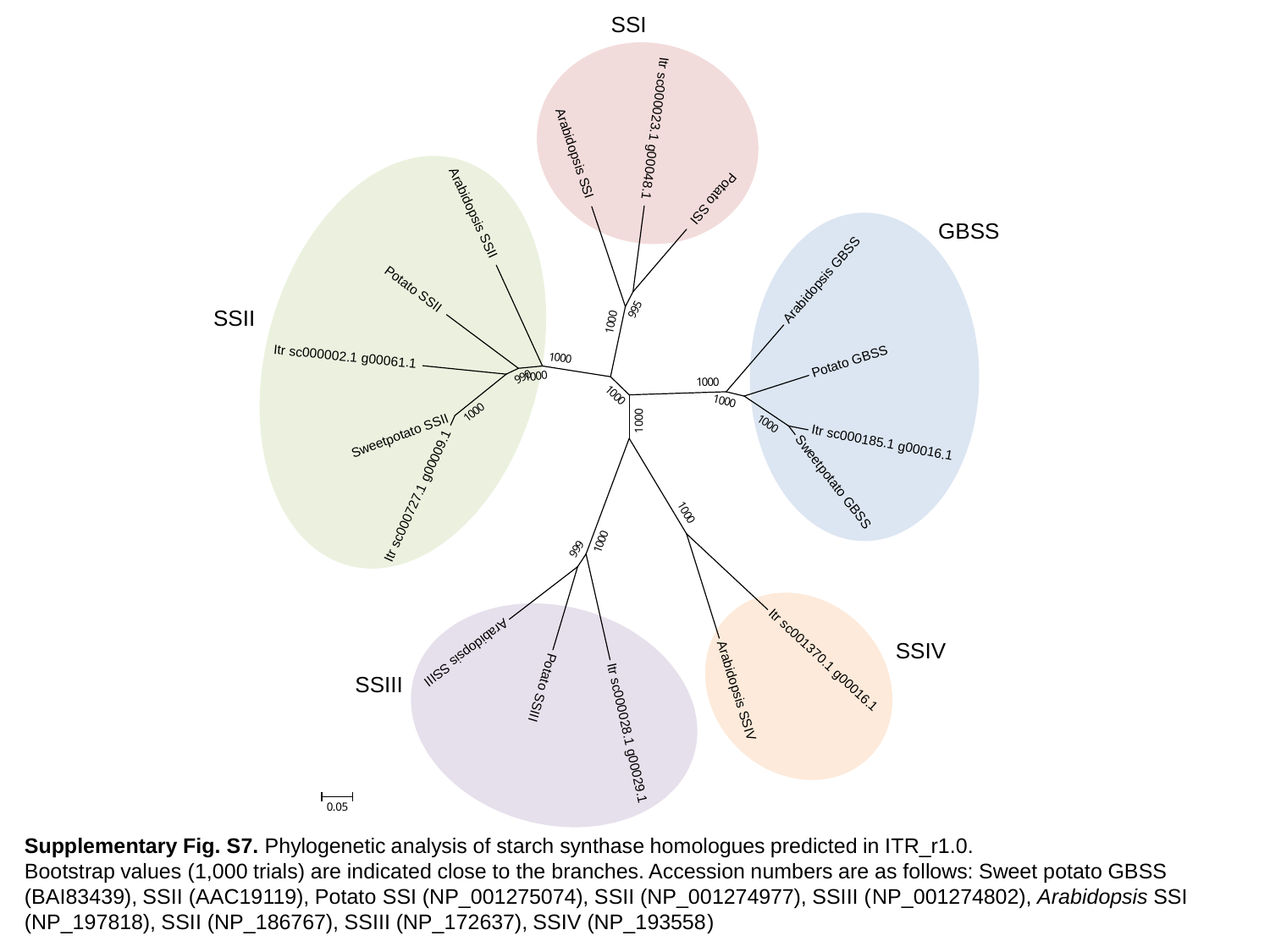

## Slide 14
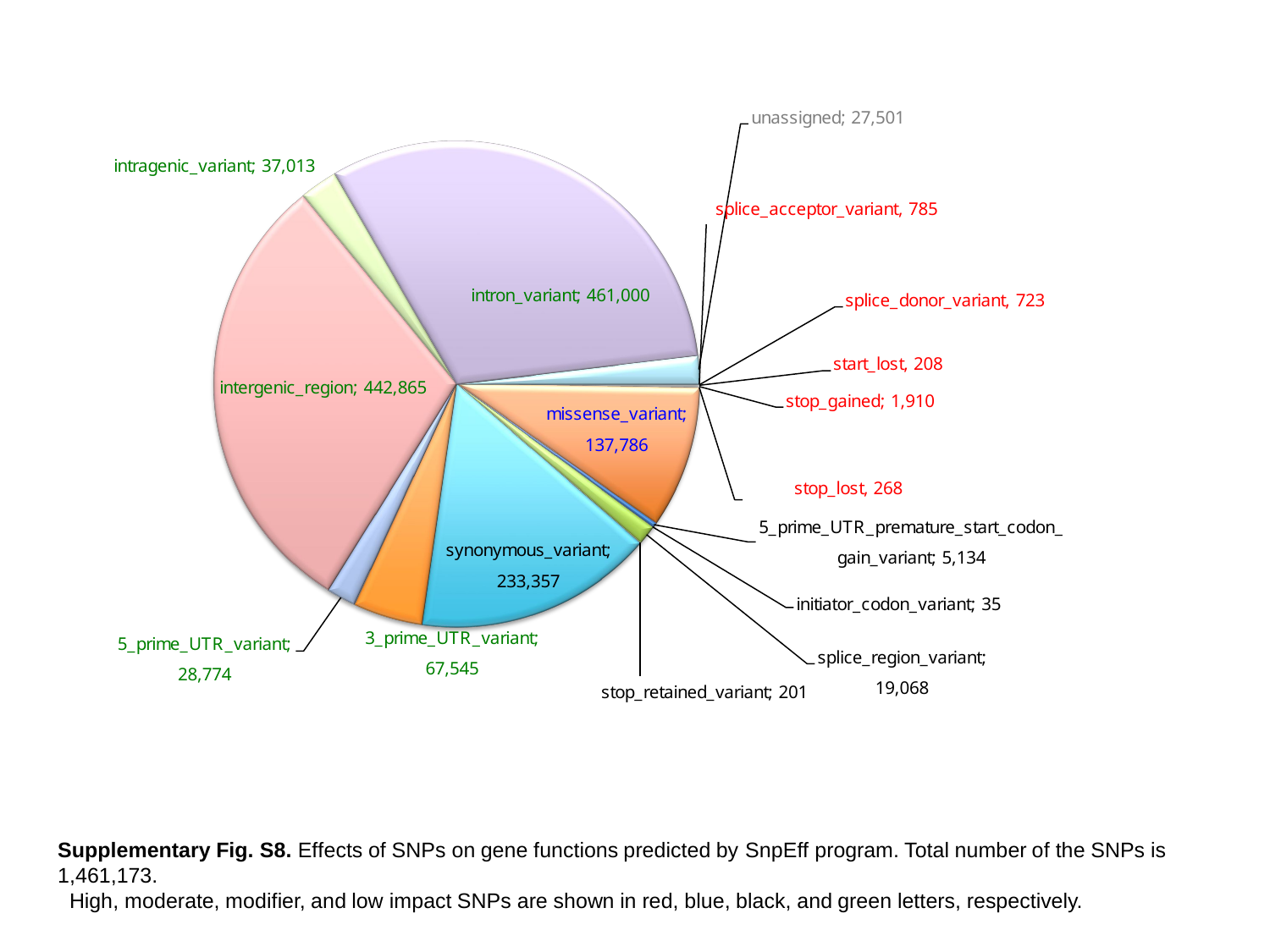

## Slide 15
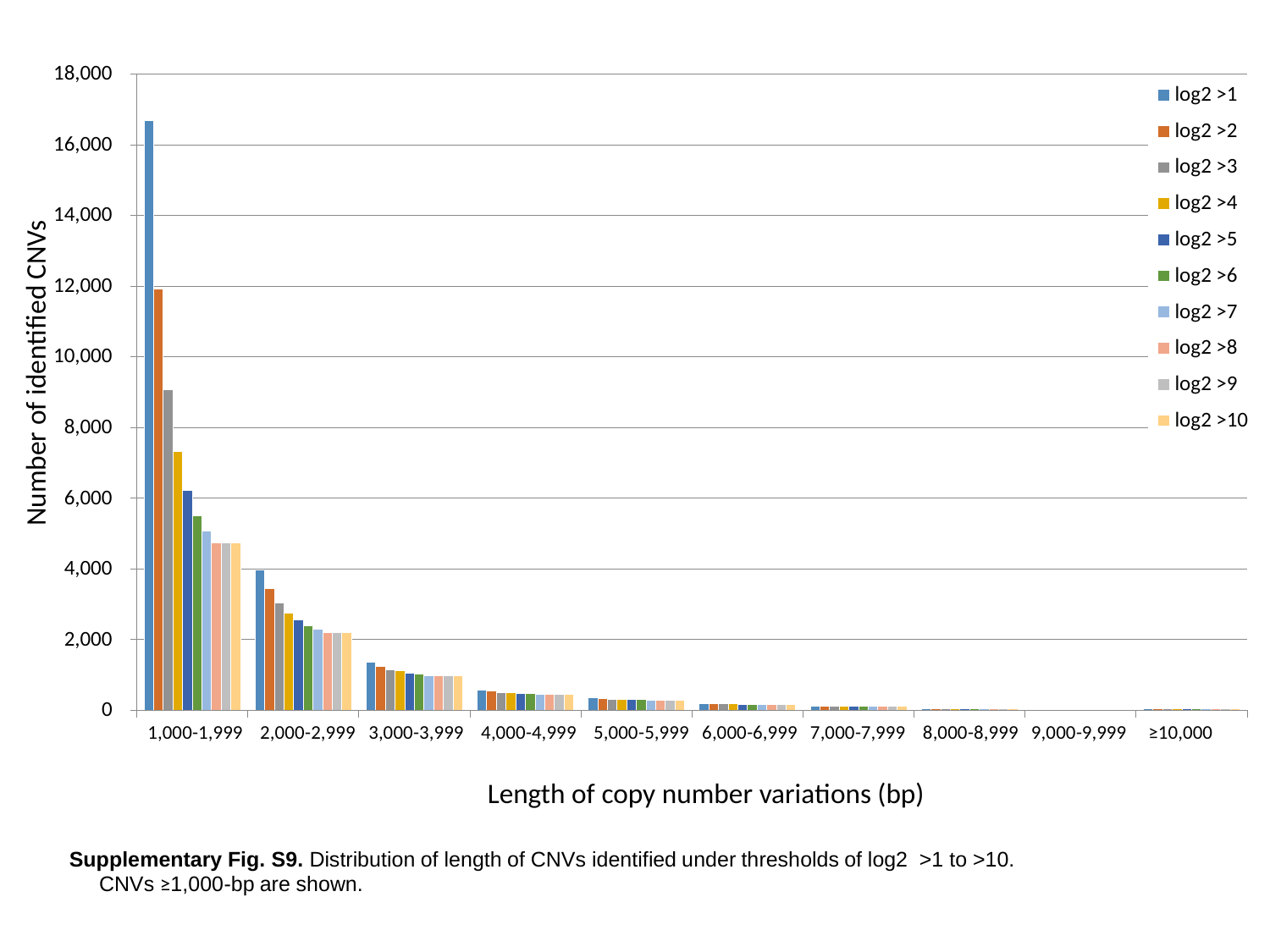

## Slide 16
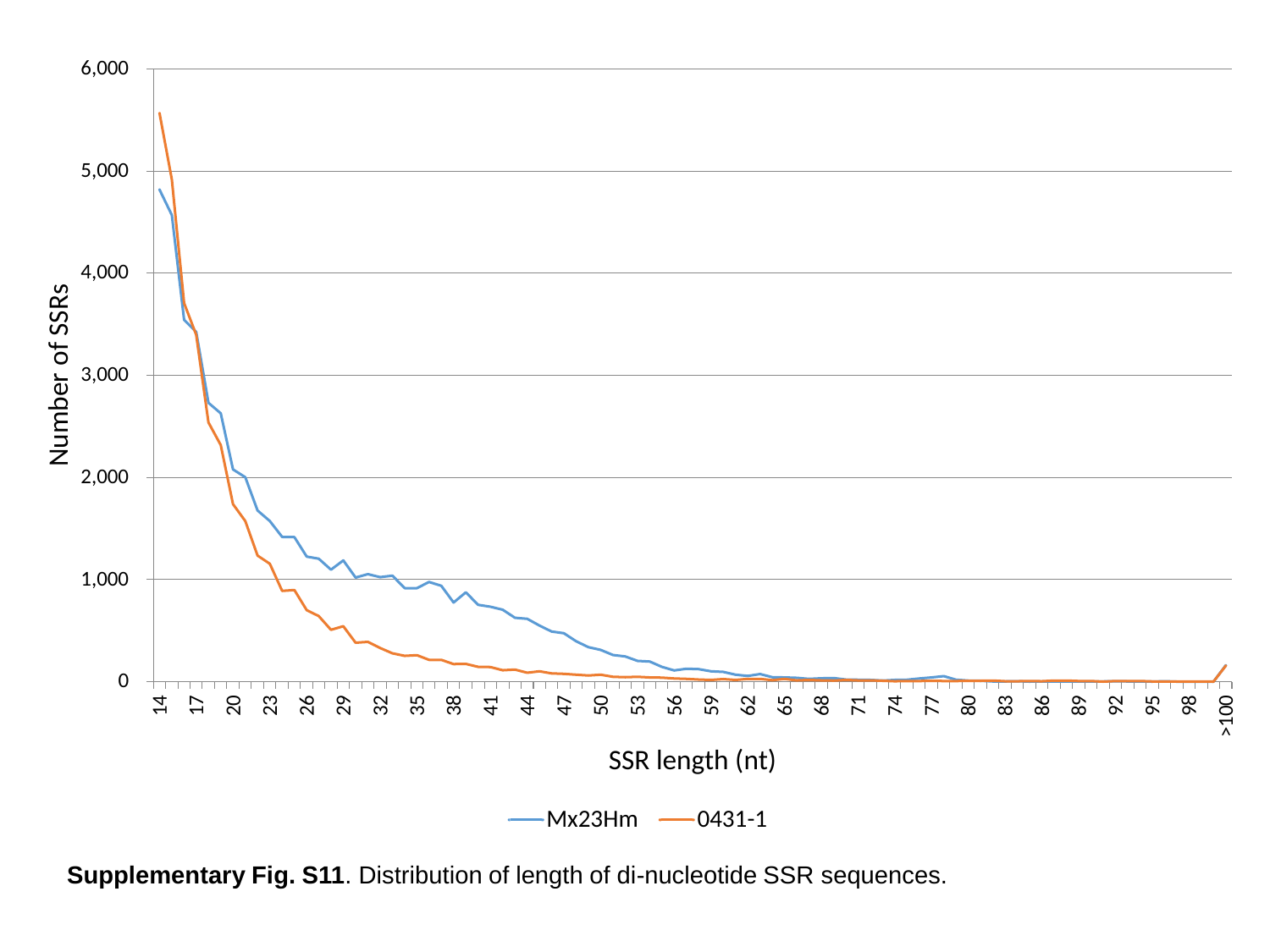

## Slide 17
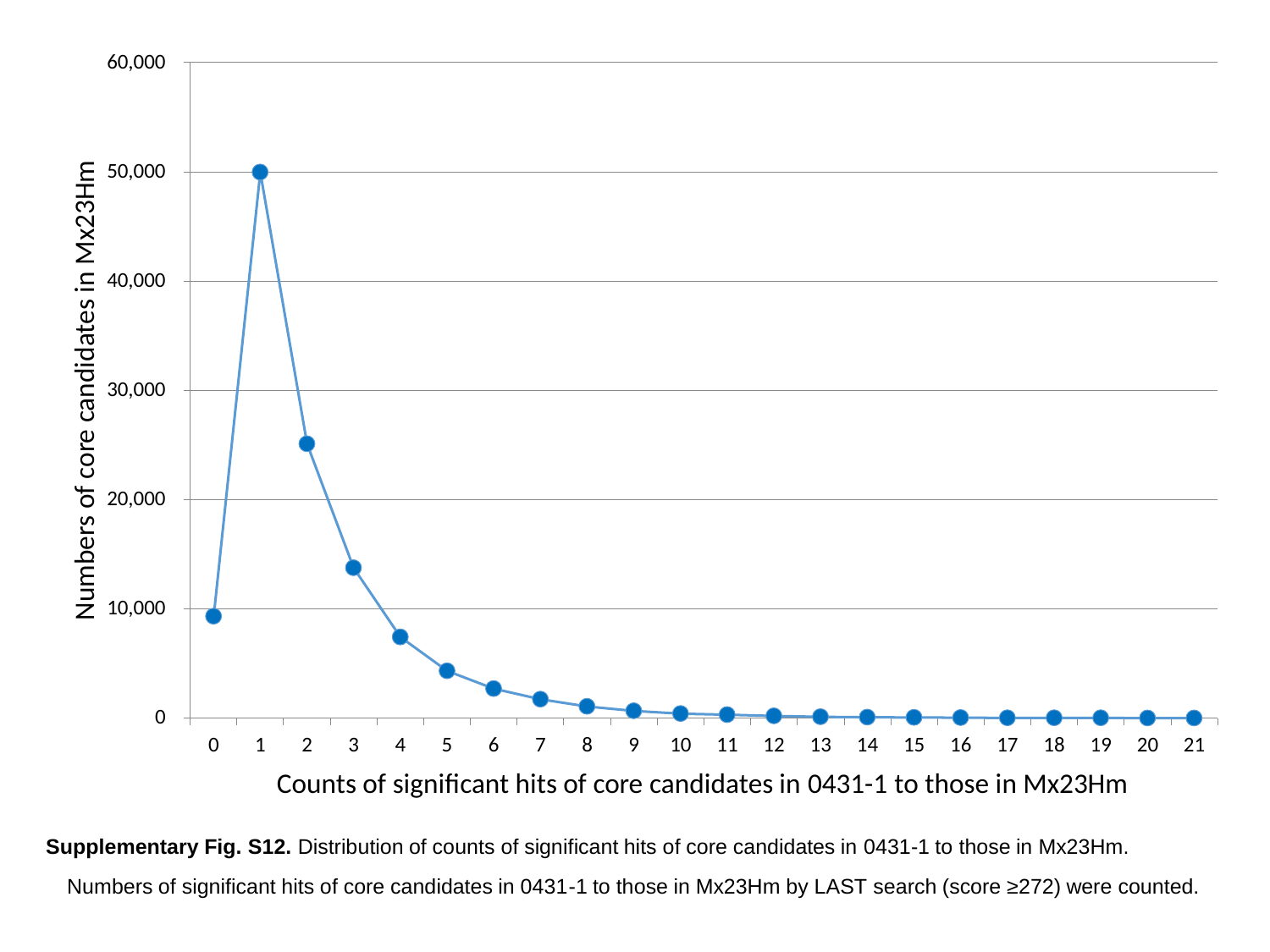

Supplement: Supplementary Data [file supp_dsv002_dsv002supp_figures.pptx]
